# Identification of genetic effects underlying type 2 diabetes in South Asian and European populations

**DOI:** 10.1038/s42003-022-03248-5

**Published:** 2022-04-07

**Authors:** Marie Loh, Weihua Zhang, Hong Kiat Ng, Katharina Schmid, Amel Lamri, Lin Tong, Meraj Ahmad, Jung-Jin Lee, Maggie C. Y. Ng, Lauren E. Petty, Cassandra N. Spracklen, Fumihiko Takeuchi, Md. Tariqul Islam, Farzana Jasmine, Anuradhani Kasturiratne, Muhammad Kibriya, Karen L. Mohlke, Guillaume Paré, Gauri Prasad, Mohammad Shahriar, Miao Ling Chee, H. Janaka de Silva, James C. Engert, Hertzel C. Gerstein, K. Radha Mani, Charumathi Sabanayagam, Marijana Vujkovic, Ananda R. Wickremasinghe, Tien Yin Wong, Chittaranjan S. Yajnik, Salim Yusuf, Habibul Ahsan, Dwaipayan Bharadwaj, Sonia S. Anand, Jennifer E. Below, Michael Boehnke, Donald W. Bowden, Giriraj R. Chandak, Ching-Yu Cheng, Norihiro Kato, Anubha Mahajan, Xueling Sim, Mark I. McCarthy, Andrew P. Morris, Jaspal S. Kooner, Danish Saleheen, John C. Chambers

**Affiliations:** 1 Lee Kong Chian School of Medicine, Nanyang Technological University, Singapore, 308232 Singapore; 2 Department of Epidemiology and Biostatistics, Imperial College London, London, W2 1PG UK; 3 Department of Cardiology, Ealing Hospital, London North West Healthcare NHS Trust, Middlesex, UB1 3HW UK; 4 Institute of Computational Biology, Deutsches Forschungszentrum für Gesundheit und Umwelt, Helmholtz Zentrum München, 85764 Neuherberg, Germany; 5 Department of Informatics, Technical University of Munich, 85748 Garching bei München, Neuherberg, Germany; 6 Department of Medicine, McMaster University, Hamilton, ON Canada; 7 Population Health Research Institute, Hamilton Health Sciences and McMaster University, Hamilton, ON Canada; 8 The University of Chicago, Biological Sciences Division, Public Health Sciences, 5841 South Maryland Avenue, MC2000, Chicago, IL 60637 USA; 9 Genomic Research on Complex diseases, CSIR-Centre for Cellular and Molecular Biology (CSIR-CCMB), Hyderabad, India; 10 Translational Medicine and Human Genetics, Department of Medicine, University of Pennsylvania, Philadelphia, Pennsylvania USA; 11 Department of Medicine, Mayo Hospital, Lahore, Pakistan; 12 Center for Genomics and Personalized Medicine Research, Center for Diabetes Research, Wake Forest School of Medicine, Winston-Salem, NC 37215 USA; 13 Vanderbilt Genetics Institute, Division of Genetic Medicine, Vanderbilt University Medical Center, Nashville, TN 37232 USA; 14 Human Genetics Center, School of Public Health, The University of Texas Health Science Center at Houston, Houston, TX 77030 USA; 15 Department of Genetics, University of North Carolina at Chapel Hill, Chapel Hill, NC 27599 USA; 16 Department of Biostatistics and Epidemiology, University of Massachusetts, Amherst, MA 01003 USA; 17 Department of Gene Diagnostics and Therapeutics, Research Institute, National Center for Global Health and Medicine, Tokyo, Japan; 18 U Chicago Research Bangladesh, House#4, Road#2b, Sector#4, Uttara, Dhaka, 1230 Bangladesh; 19 Department of Public Health, Faculty of Medicine, University of Kelaniya, Kelaniya, Sri Lanka; 20 Department of Pathology and Molecular Medicine, McMaster University, Hamilton, ON Canada; 21 Academy of Scientific and Innovative Research, CSIR-Institute of Genomics and Integrative Biology Campus, New Delhi, 110020 India; 22 Systems Genomics Laboratory, School of Biotechnology, Jawaharlal Nehru University, New Delhi, 110067 India; 23 Singapore Eye Research Institute, Singapore National Eye Centre, Singapore, Singapore; 24 Department of Medicine, Faculty of Medicine, University of Kelaniya, Kelaniya, Sri Lanka; 25 Department of Medicine, McGill University, Montreal, QC Canada; 26 Department of Human Genetics, McGill University, Montreal, QC Canada; 27 Department of Health Research Methods, Evidence, and Impact, McMaster University, Hamilton, ON Canada; 28 Ophthalmology & Visual Sciences Academic Clinical Program (Eye ACP), Duke-NUS Medical School, Singapore, Singapore; 29 Department of Biostatistics and Epidemiology, University of Pennsylvania, Philadelphia, Pennsylvania 19104 USA; 30 Department of Ophthalmology, Yong Loo Lin School of Medicine, National University of Singapore, Singapore, Singapore; 31 Diabetes Unit, King Edward Memorial Hospital and Research Centre, Pune, India; 32 Department of Biostatistics and Center for Statistical Genetics, University of Michigan, Ann Arbor, Michigan 48109 USA; 33 Department of Biochemistry, Wake Forest School of Medicine, Winston-Salem, NC 37215 USA; 34 JSS Academy of Health Education of Research, Mysuru, India; 35 Science and Engineering Research Board, Department of Science and Technology, Ministry of Science and technology, Government of India, New Delhi, India; 36 Oxford Centre for Diabetes, Endocrinology and Metabolism, Radcliffe Department of Medicine, University of Oxford, Oxford, OX3 7BN UK; 37 Wellcome Centre for Human Genetics, Nuffield Department of Medicine, University of Oxford, Oxford, OX3 7BN UK; 38 Saw Swee Hock School of Public Health, National University of Singapore, Singapore, Singapore; 39 Oxford NIHR Biomedical Research Centre, Churchill Hosptial, Oxford University Hospitals NHS Foundation Trust, Oxford, OX3 7LE UK; 40 Department of Biostatistics, University of Liverpool, Liverpool, L69 3GL UK; 41 Estonian Genome Center, Institute of Genomics, University of Tartu, Tartu, Estonia; 42 Wellcome Trust Centre for Human Genetics, University of Oxford, Oxford, OX3 7BN UK; 43 Imperial College Healthcare NHS Trust, Imperial College London, London, W12 0HS UK; 44 MRC-PHE Centre for Enviroment and Health, Imperial College London, London, W2 1PG UK; 45 National Heart and Lung Institute, Imperial College London, London, W12 0NN UK; 46 Center for Non-Communicable Diseases, Karachi, Pakistan; 47 Department of Medicine, Columbia University Irving Medical Center, New York, NY 10032 USA; 48 Department of Cardiology, Columbia University Irving Medical Center, New York, NY 10032 USA

**Keywords:** Genetic variation, Diabetes

## Abstract

South Asians are at high risk of developing type 2 diabetes (T2D). We carried out a genome-wide association meta-analysis with South Asian T2D cases (*n* = 16,677) and controls (*n* = 33,856), followed by combined analyses with Europeans (n_eff_ = 231,420). We identify 21 novel genetic loci for significant association with T2D (*P* = 4.7 × 10^−8^ to 5.2 × 10^−12^), to the best of our knowledge at the point of analysis. The loci are enriched for regulatory features, including DNA methylation and gene expression in relevant tissues, and highlight *CHMP4B*, *PDHB*, *LRIG1* and other genes linked to adiposity and glucose metabolism. A polygenic risk score based on South Asian-derived summary statistics shows ~4-fold higher risk for T2D between the top and bottom quartile. Our results provide further insights into the genetic mechanisms underlying T2D, and highlight the opportunities for discovery from joint analysis of data from across ancestral populations.

## Abstract (web summary)

Marie Loh, Weihua Zhang et al. use a genome-wide association study meta-analysis to examine variants associated with Type 2 diabetes (T2D) in South Asian and European ancestry cohorts. Their results provide further insights into the genetic mechanisms underlying T2D across ancestral populations.

## Introduction

Type 2 diabetes (T2D) is a major public health problem that currently affects 425 million people worldwide^1^. The burden of diabetes is especially high in South Asia, the most densely populated region of the world (~1.9 billion people in 2019, ~24.9% of world population)^2^. India alone is home to 72 million people with T2D^1^. In urban settings, risk of T2D in South Asians is four-fold higher than Europeans^3^, with T2D developing 10 years earlier^4^. The increased risk of T2D in South Asians is not accounted for by traditional risk factors such as obesity, diet and physical inactivity, which has led to the hypothesis that South Asians may have increased genetic susceptibility^5^.

To date, sequence variants at more than 400 genetic loci have been implicated in the aetiology of T2D through genome-wide association studies (GWAS) that have been carried out predominantly amongst people of European ancestry^6–12^. Although many of these risk relationships are cosmopolitan, population-specific studies have revealed genetic variants associated with T2D that are common in their respective ethnic groups, but rare or absent elsewhere^3,13–19^. Recent studies have highlighted the extensive genetic diversity that exists in South Asia^20–26^, and which provides the basis for investigating the possibility of distinct disease relationships, in addition to cosmopolitan effects. There is a well recognised need for deeper investigation of the genetic basis for T2D and other chronic diseases in South Asian populations^27^.

## Results

### Genome-wide meta-analysis

As part of the DIAbetes Meta-ANalysis of Trans-Ethnic association studies (DIAMANTE) project, we combined data from 16 South Asian case-control and population-based cohorts, to complete the largest discovery genome-wide meta-analysis for T2D in this ethnic group to date (16,677 cases and 33,856 controls). Characteristics of participants, information on the genotyping arrays and imputation are detailed in Supplementary Data 1 and 2. We performed two primary association analyses in body mass index (BMI) unadjusted and BMI-adjusted models. At a *P*-value threshold of 5 × 10^−8^, a total of 372 and 440 SNPs, distributed across 10 and 14 gene loci, reached statistical significance in the BMI-unadjusted and adjusted models, respectively (Supplementary Data 3; Supplementary Figs. 1, 2). All SNPs identified at this stage were from genetic loci known to be associated with T2D (Supplementary Data 4), arguing against an important contribution of population-specific variants to the risk of T2D in South Asians.

Next, we carried out a combined analysis of GWAS data for South Asians and Europeans to identify genetic effects that are shared between the populations. To the best of our knowledge at the point of analysis, we selected all potentially novel SNP-phenotype associations at *P* < 1 × 10^−3^ in either the BMI unadjusted (*N* = 17,944) or the BMI-adjusted (*N* = 17,215) model amongst South Asians, for further testing. We limited the analysis to the SNPs with the lowest *P*-values for association with T2D in South Asians, to reduce the burden of multiple testing, and to help prioritise genetic variants that may make a more important contribution in South Asians. We performed fixed effects meta-analyses to combine results from South Asians with data from Europeans within the DIAMANTE consortium (effective sample size = 231,420 and 157,384 for BMI-unadjusted and adjusted models, respectively). We chose to combine data with Europeans, as the major global population group most closely genetically related to South Asians^10,16,28,29^. The genetic correlation between South Asians and Europeans for T2D is 0.89 (SE: 0.06), which is higher than reported between East Asians and Europeans at 0.62 (SE: 0.09)^30^, providing further support for this choice. We found that 218 and 185 novel SNPs across 14 and 11 loci reached genome-wide significance (*P* = 4.7 × 10^−8^ to 5.2 × 10^−12^) in the BMI-unadjusted and adjusted models, respectively, (Supplementary Data 5 and 6). Together these represent 21 novel genetic loci, of which 12 showed a high degree of statistical stringency (*P* < 5 × 10^−9^). Sentinel SNP for each locus was defined as the SNP with the lowest *p*-values within a ±500kb region. Conditional analysis confirms the 21 sentinel SNPs to be associated with T2D, independent of previously reported T2D genetic relationships (Supplementary Data 7; Table 1). Regional plots for the 21 loci are shown in Supplementary Figs. 3–44. Whilst the majority of our newly identified associations represent common genetic variants with modest effect size (OR for T2D < 1.2), we also identify a low-frequency genetic variant (rs76141923), which has a moderately-high risk on T2D (OR = 1.40; 95% CI: 1.26–1.57, minor allele frequency in SAS: 1%). There was no evidence for heterogeneity between the results for the association of the genetic variants with T2D between the analyses with and without BMI adjustment (all *P* > 0.003; Bonferroni corrected for 21 loci; Supplementary Data 8**)**. Sex-specific analysis did not reveal any additional significant findings (Supplementary Data 9).

**Table 1:** Genetic loci newly identified to be associated with Type 2 Diabetes (T2D).

Sentinel SNP	Chr	Pos	Transcript	EA	EAF	N	OR (95% CI)	P	Association Model
rs10916784	1	20729451	*VWA5B1*^m^	G	0.58	197,391	1.05 (1.03–1.06)	5.2E−12	BMI-adjusted
rs7531962	1	146746023	*CHD1L*^e,n^*, FMO5*^e^, *PRKAB2*^*e,m*^	A	0.11	197,391	1.06 (1.04–1.08)	2.6E−08	BMI-adjusted
rs12463719	2	203450680	*BMPR2*^e,m^, *FAM117B*^e^*, NOP58*^e^, *RP11-686O6.2*^*e*^	A	0.32	272,634	1.04 (1.03–1.06)	1.2E−10	BMI-unadjusted
rs7432739	3	58364650	*ABHD6*^e^, *DNASE1L3*^e^, *FLNB*^m^, *KCTD6*^m^, *PDHB*^e^, *PXK*^e,n^, *RP11-802O23.3*^e^, *RPP14*^e^	G	0.28	197,391	1.05 (1.03–1.06)	5.4E−09	BMI-adjusted
rs7626079	3	66427259	*LRIG1*^e^*, SLC25A26*^n,e,m^	C	0.64	197,391	1.04 (1.03–1.06)	8.9E−10	BMI-adjusted
rs62366901	5	49930286	*EMB*^*e*,m^*, PARP8*^e*^	T	0.60	197,080	1.04 (1.03–1.05)	1.3E−08	BMI-adjusted
rs74790763	5	122675214	*CEP120*^n,ns,m^	C	0.94	272,634	1.10 (1.07–1.14)	3.2E−11	BMI-unadjusted
rs7765207	6	138858054	*ABRACL*^e^*, CCDC28A*^e^, *HEBP2*^e^*, NHSL1*^n,e*,m^	T	0.39	197,391	1.04 (1.03–1.06)	4.2E−09	BMI-adjusted
rs73689877	7	40905274	*INHBA*^e^*, SUGCT*^n,m^	A	0.12	272,634	1.06 (1.04–1.08)	5.7E−09	BMI-unadjusted

Sentinel SNP	Chr	Pos	Gene	EA	EAF	N	OR (95% CI)	P	Association Model
rs2980766	8	8318095	*CLDN23*^e^, CTA-398F10.2e*, ENPP7P1^e^, *ERI1*^e^, *FAM85B*^e^, *FAM86B3P*^e^, *FAM90A9*^m^, *MFHAS1*^e,m^, *PRAG1*^e,m^*, RP11-10A14.5*^e^, *RP11-115J16.2*^e^, *RP11-62H7.2*^e^*, SGK223*^e^	C	0.48	264,876	1.04 (1.03–1.05)	7.5E−11	BMI-unadjusted
rs62486442	8	12623463	*LONRF1*^e,m^	A	0.34	190,682	1.05 (1.03–1.06)	2.4E−10	BMI-adjusted
rs13257283	8	105608497	*LRP12*^n,e,m^	G	0.92	272,634	1.07 (1.04–1.09)	4.2E−08	BMI-unadjusted
rs2488597	9	125992073	*RABGAP1*^e*,m^, *RC3H2*^e^, *STRBP*^n,e^, *ZBTB6*^e^	A	0.84	272,634	1.05 (1.03–1.07)	2.8E−09	BMI-unadjusted
rs2114824	10	88119015	*GRID1*^n,e,m^*, WAPAL*^e^	G	0.47	271,738	1.03 (1.02–1.04)	4.7E−08	BMI-unadjusted
rs10748694	10	99056190	*ARHGAP19*^n,e*^, *ARHGAP19-SLIT1*^e^*, AVPI1*^e^*, EXOSC1*^e^*, FRAT1*^*e,m*^*, FRAT2*^*e,m*^, *MMS19*^e^, *PGAM1*^*e*^, *RRP12*^e^*, SLIT1*^e^	A	0.42	272,634	1.04 (1.03–1.05)	1.8E−10	BMI-unadjusted
rs7123361	11	76161389	*EMSY*^n,m^*, LRRC32*^e^	A	0.69	228,651	1.04 (1.03–1.05)	1.4E−08	BMI-unadjusted
rs9568861	13	54079446	*OLFM4*^e^	T	0.15	272,634	1.05 (1.03–1.06)	2.5E−08	BMI-unadjusted
rs76141923	13	81646630	*–*	C	0.01	186,208	1.40 (1.26–1.57)	1.4E−09	BMI-adjusted
rs28790585	15	39668870	*C15orf54*^e^, *THBS1*^e*,m^	T	0.33	197,391	1.04 (1.03–1.06)	4.1E−08	BMI-adjusted

Sentinel SNP	Chr	Pos	Gene	EA	EAF	N	OR (95% CI)	P	Association Model
rs7261425	20	20068635	*CFAP61*^n,e*,m^*, CRNKL1*^e^, *INSM1*^e*^	C	0.71	272,634	1.04 (1.03–1.05)	4.3E−10	BMI-unadjusted
rs2065703	20	31966698	*CBFA2T2*^e,m^, *CDK5RAP1*^n^*, CHMP4B*^e^, *E2F1*^e^, *EIF2S2*^e^, *NECAB3*^e^, *PXMP4*^e^, *SNTA1*^e^	T	0.15	197,391	1.06 (1.04–1.08)	6.8E−11	BMI-adjusted

Candidate genes are annotated by the nature of the variant: n, nearby gene (10 kb).

*e* cis expression quantitative trait locus (eQTL), e* colocolized cis eQTL, *m* cis DNA methylation marker. Position is based on Build 37 of the reference genome. *Chr* chromosome, *EA* effect allele, *AA* alternate allele, *EAF* effect allele frequency in South Asians.

We used Genome-wide Complex Trait Analysis (GCTA) of the results for South Asians to seek for additional independent signals at the 21 novel loci; there were no signals that reached a locus-wide significance threshold of *P* < 1 × 10^−5^. The heritability for T2D in South Asians is estimated at ~12% by GREML in GCTA using our largest available cohort (LOLIPOP-IA610). The total T2D (trait) variance explained by the independent T2D-associated loci (both known and novel) after COJO was estimated at 10.6%. Taking a genome-wide approach, heritability was estimated at 35 and 50% in the IA317 and IA610 South Asian datasets, respectively. This is similar to that reported in Europeans^31,32^.

### Cross-ancestry comparisons

We next tested our 21 novel genetic loci for association with T2D in East Asian, African and Hispanic populations, ethnic groups not included in the discovery meta-analysis. Effective sample sizes in East Asians were 211,793 and 135,780 for the BMI-unadjusted and adjusted analyses, respectively, providing high statistical power (>90%) to identify the effect sizes observed in our South Asian samples, at *P* = 0.05. We found that 12 of our sentinel SNPs replicated in East Asians in the BMI-unadjusted model (*P* < 0.05 and same direction of effect, Supplementary Data 10). Amongst the nine sentinel SNPs that did not replicate, three show evidence for heterogeneity of effect (Supplementary Figs. 45–48), while four are low frequency or monomorphic, in East Asians. Findings were similar in the BMI-adjusted model (Supplementary Data 11; Supplementary Figs. 46–49). Effective sample sizes in African and Hispanic populations were lower (BMI unadjusted: *N* = 36,991 and 32,037; BMI adjusted *N* = 36,345 and 20,499, respectively). Although power to replicate the associations identified in South Asians ranged from 30 to 95% (mean 51%, Supplementary Fig. 50), we found that only one of the sentinel SNPs replicated in Africans, while none replicated in Hispanics (Supplementary Figs. 45–48).

There was limited evidence for heterogeneity of effect on T2D, between South Asians and Europeans at the 21 novel genetic loci (Supplementary Data 10 and 11, Supplementary Figs. 45–48); this is not unexpected in light of our strategy comprising meta-analysis of data from the two populations as the basis for discovery. We also find no evidence for heterogeneity of effect on the risk of sentinel SNPS on risk of T2D between South Asians and Europeans, at 237 (82%) of the 288 genetic loci reported to be associated with T2D (Supplementary Data 12 and 13, Supplementary Figs. 51 and 52). Taken together, the above results suggest that there is little heterogeneity between Europeans and South Asians, which supports out approach of meta-analysis across these population groups, to maximise power for the identification of shared effects. To extend on this analysis, we investigated all SNPs at *P* < 1 × 10^−3^ in either the BMI unadjusted (*N* = 17,944) or the BMI-adjusted (*N* = 17,215) model in the South Asians (Supplementary Data 14 and 15).

### Functional genomic analyses

We identified potential candidate genes based upon proximity (genes within 10 kb of the sentinel SNP), and functional genomic information (gene expression and regulatory DNA methylation) (Table 1). We searched for *cis* expression quantitative trait loci (eQTLs) using eQTL data generated from whole blood gene expression data from 31,684 individuals by the eQTLGen Consortium^33^. We found an enrichment of *cis* eQTLs among the novel SNPs (*P* < 1.8 × 10^−308^, compared to expectation under the null hypothesis [1-sample t-test]), with 47 unique significant *cis* eQTLs present for 16 of the 21 sentinel SNPs or their proxies (*r*^2^ ≥ 0.8) at a Bonferroni corrected *p*-value threshold of <6.8 × 10^fto^ (Table 1; Supplementary Data 16). To investigate the robustness of the identified eQTLs in South Asians, we performed lookups in our eQTL dataset derived from 693 South Asians, where we replicated 37% of the eQTLs across 9 of the loci (16 out of 43 eQTLs available in the South Asian dataset). Among the 63% that did not replicate, none of them were sufficiently powered to detect the effect at 80% power and 5% significance level. We further supplemented our analysis by interrogating islet-specific *cis* eQTLs^8,34,35^. Here we observed 50 unique significant *cis* eQTLs with 17 of the novel sentinel SNPs (*P* < 0.05; Supplementary Data 17; enrichment [1-sample t-test] *P* = 3.0 × 10^−132^), of which 13 replicated those that were found in the eQTLGen analysis, suggesting that the novel loci implicates both generic and tissue-specific eQTLs. In addition, at three of the novel loci, T2D signals colocalized with respective cis-eQTLs for *CHMP4B*, *PDHB* and *LRIG1* in both BMI-unadjusted and adjusted models (Table 1; Supplementary Data 18 and 19).

To better understand the disturbances in genomic regulation underlying T2D, we tested the association of the sentinel SNPs with DNA methylation, in South Asians (*N* = 1841). DNA methylation was quantified in peripheral blood by Illumina HumanMethylation450 array^36^. We found that 19 of the sentinel SNPs have one or more *cis* methylation quantitative trait loci (methQTL) associations at *P* < 8.2 × 10^−6^ (*P* < 0.05 after Bonferroni correction for the 6,065 SNP-CpG association tests; Supplementary Data 20), which represents a ~2-fold enrichment in cis methQTLs observed (enrichment [1-sample *t*-test]: *P* < 1.8 × 10^−308^). All methQTLs replicated in independent testing in 1354 South Asians (*P* < 0.05 with Bonferroni correction and consistent direction of effect; Supplementary Data 21). We mapped methQTLs to potential candidate genes using Illumina annotation files, supplemented by gene proximity, and noted that at nine loci, although we found both significant eQTLs and methQTLs with our sentinel SNPs, there was no evidence for colocalization observed (Table 1; Supplementary Data 22). In part, this may reflect the lower sample size available for functional genomic studies in South Asians.

### Functional annotation and cross-trait association

Detailed functional annotation for sentinel SNPs at the 21 novel loci is provided in Supplementary Data 23. VEP (Variant Effect Predictor) was used to annotate the sentinel SNPs and their proxies (*r*^2^ > 0.8) for nonsynonymous or splice site variations, and the presence of transcription factor binding sites (TFBS)^37^. None of the SNPs or their proxies was nonsynonymous. Two were splice site variants, seven SNPs overlap known TFBS and SNPs at four loci overlap CpG islands (Supplementary Data 23).

We used HaploReg v4.1 (Broad Institute) to identify SNPs overlapping regulatory regions (protein binding and regulatory motifs, promoter and enhancer histone marks, and DNase I hypersensitive sites (DHS))^38^. There was significant enrichment for SNPs and/or proxies for promoter histone marks in at least one tissue (*n* = 13; *P* = 1.92 × 10^−130^), as well as SNPs or proxies overlapping with enhancer histone marks or DHS in at least one tissue (*n* = 19; *P* = 2.16 × 10^−45^ and *n* = 17; *P* = 1.52 × 10^−19^, respectively). PhenoScanner, an on-line tool which searches 88 complex trait GWAMAs and three GWAS catalogues, was used to annotate the 21 novel loci for association with other complex traits (*P* < 1 × 10^−5^)^39,40^. As has been observed previously, we found associations with numerous metabolic phenotypes such as systolic and diastolic blood pressure/hypertension, waist circumference and BMI (Supplementary Data 24)^41–46^.

### Polygenic risk scores for risk stratification of T2D in South Asians

Studies in predominantly European populations have demonstrated the potential for polygenic risk scores to identify people at high risk of complex disease, who may show greater benefit from interventions to prevent disease development^47,48^. To advance the use of genomic information for risk stratification in South Asian populations, who are at high risk of T2D and other major chronic diseases, we used the LDpred algorithm to derive a polygenic risk score for T2D. We used South Asian summary statistics from the current meta-analysis to derive a South Asian specific polygenic risk score (SA-PRS) for T2D. We compared performance of this model to a polygenic risk for T2D based on European summary statistics from the DIAMANTE consortium (EUR-PRS)^49^. The best-performing SA-PRS was selected based upon an initial validation dataset (IA610; *n* = 2019 cases and 3696 controls), and identified as the model with maximum area under the receiver-operator curve (AUC; proportion of causal variants, *p* = 0.01; Supplementary Data 25). For the European-derived summary statistics model, we selected the model previously reported to be optimal with *p* = 0.01^47^. We replicated model performance of the SA-PRS and EUR-PRS in two independent testing sets (SINDI; *n* = 974 cases and 1168 controls; LOLIPOP-GSA: *n* = 1000 cases and controls each).

We found that the SA-PRS model, which is based on population-specific South Asian summary statistics, shows better predictive power for T2D than a risk score based on European summary statistics in both validation and testing (Validation: AUC: 0.62 [95% CI: 0.60–0.63] vs 0.55 [95% CI: 0.54–0.57], *P* = 1.0E−10; Testing: AUC: 0.59 [95% CI: 0.58–0.61] vs 0.55 [95% CI: 0.53–0.56], *P* = 2.2E−4, Supplementary Data 26). The prevalence of T2D increased from 19 to 53% across decile bin, with risk increasing as risk score increases (*p* = 1.6 × 10^−5^; trend test). Indeed, the SA-PRS identified the top quartile of our validation population as having 4.03 (95% CI:3.36–4.85; *P* < 4.5 × 10^−50^) higher risk for T2D relative to the bottom quartile (Supplementary Fig. 53). We further showed that there were no significant improvement in AUC using summary statistics obtained from meta-analysis of South Asians and Europeans (Validation AUC: *P* = 1.000; Testing AUC: *P* = 0.356; Supplementary Data 26). Our results confirm the potential for genomic information to identify South Asian individuals susceptible to T2D.

## Discussion

We carried out a genome-wide association meta-analysis of T2D in South Asians, leveraging power for discovery by inclusion of data from additional European samples, thereby revealing 21 genetic loci newly associated with T2D at the point of analysis. Leading genetic variants at these genetic loci associated with T2D are enriched for location in DNA protein-binding sites, regulatory motifs, DHS, promoter and enhancer histone marks, and for association with gene regulatory methylation marks in blood, and with gene expression in blood and pancreatic islets. At three of the novel loci, we found that T2D signals colocalized with respective cis-eQTLs for *CHMP4B*, *PDHB* and *LRIG1*.

Genetic variants in *LRGI1* have been previously shown to be linked to birth weight, blood pressure and cardiovascular endpoints^50–53^, while methylation of CpG sites in *LRIG1* has been linked to obesity in children, as well as birthweight and maternal adiposity^54–56^. Indeed, it has been recently shown that *LRIG1* regulates lipid metabolism via BMP signalling, and that *LRIG1* variants are strongly associated with increased BMI, but with lower risk of T2D^57^. *DHB* encodes one of the components of PDH, which catalyses the irreversible oxidative decarboxylation of pyruvate into acetyl-CoA and reduces NAD+ to NADH. Levels of PDH impact upon the proportion of energy supplied by glucose relative to that from other energy-producing molecules such as lipids and amino acids, thereby playing a key role in the development of diabetes^58^. At the same time, variants in *PDHB* have been linked to BMI-adjusted waist circumference and LDL cholesterol levels^59,60^.

For the nine genes where we found both significant eQTLs and methQTLs with our sentinel SNPs, we did not observe significant colocalization. This may reflect the choice of blood as the tissue for investigation, or poor coverage of the methylation markers, given that the methylation array is sparse and covers only ~2% of the methylome, and therefore do not identify the most important causal methylation site. In addition, despite being one of the largest DNA methylation datasets available, the sample size for the DNA methylation analysis is ~3200, which may limit the power of the colocalization experiment.

Although our observations support the view that the genetic variants we identify to be associated with T2D are likely to be of functionally importance, and operate primarily through an effect on genomic regulation, which is consistent with findings from other large-scale genetic association studies^9,61,62^, there is no direct evidence that DNA methylation is part of the casual chain leading to T2D.

Our study includes data from 50,533 South Asians; this represents the largest study of T2D genetics in this population to date. We leverage discovery through combined analysis of our leading results with data from a parallel study of 898,130 Europeans, confirming the utility of combining results from different ethnic groups through increased sample size. We also used data from large-scale GWAS of T2D in East Asian, African and Hispanic populations to examine whether our associations are cosmopolitan or specific. We show that many of the sentinel SNPs are not associated with T2D in East Asians, despite their large sample size and high statistical power. In most instances, the sentinel SNPs which appear specific to South Asians are low frequency, rare or even monomorphic in East Asians. Similarly, only a minority of our sentinel SNPs were associated with T2D in African and Mexican populations. Our results provide further support for the presence of population-specific genetic variation contributing to chronic disease, and the strong rationale for large-scale studies focused on this ethnic group that have at least equivalent sample size to current efforts in Europeans. Such studies will be an important source of discovery, and will help to reverse the current bias in genomic research towards investigations of predominantly European populations^27^.

We also investigate the application of polygenic risk scores for the prediction of T2D in South Asian populations. We show that polygenic risk scores based on South Asian specific GWA data are more predictive for T2D than scores based on results from European specific studies. Our South Asian polygenic risk score identifies the top quartile of the population to be at ~4-fold higher risk for T2D relative to the bottom quartile, confirming the potential for genetic risk scores to identify susceptible individuals. In addition, we extend previous observations and show that the polygenic risk scores for prediction of T2D in South Asians, show improved performance when based on South Asian rather than European association test results. This inferior performance of the PRS model based on European data likely reflects heterogeneity of effect, relative to that observed in South Asians.

In summary, we identify 21 novel genetic loci associated with T2D at the point of analysis. Whilst many may be cosmopolitan, several do not appear to be shared by East Asian, African and Hispanic populations. Our results provide further insights into the genetic mechanisms underlying T2D, and reinforce the importance of extending the investigation of susceptibility to T2D and other chronic diseases to non-European populations.

## Methods

### Statistics and reproducibility

Sixteen cohorts of South Asians with 50,533 participants were included in the analysis (16,677 cases and 33,856 non-prediabetic normal controls). Effective sample size (Neff) was calculated as 4/(1/N_cases + 1/N_controls), where N_cases is the number of cases and N_controls is the number of controls. For genome-wide meta-analyses, two primary models of logistic regression association analyses were carried out with or without BMI adjustment. Age, sex, and cohort-specific covariates were included where applicable, with the first 3–15 study-specific principal components included as covariates to correct for population substructure. No technical replicates were included in the analysis. The current sample size allows us to detect significant associations at *P*-value < 5 × 10^−8^ for SNPs with MAF ≥ 0.05 and odds ratios (OR) ≥ 1.21, or MAF ≥ 0.20 and OR ≥ 1.11 at 80% power, taking an additive disease model.

### Cohort information, definition of T2D cases and normoglycaemic controls

A total of 50,533 participants (16,677 cases and 33,856 non-prediabetic normal controls) across sixteen South Asians cohorts were included in the meta-analysis. T2D cases were defined as having any one of the following: medical history of T2D or T2D treatment, fasting plasma glucose concentration ≥7.0 mmol/L, plasma glucose concentration at 2 h of OGTT ≥ 11.1 mmol/L, or HbA1c ≥ 6.5%. Normoglycaemic controls were defined as meeting all of the following criteria (where data are available): no history of T2D or T2D treatment, fasting plasma glucose <6.1 mmol/L, plasma glucose concentration at 2 h of OGTT < 7.8 mmol/L, and HbA1c < 6.0%. Prediabetic participants who are neither T2D cases nor normoglycaemic controls were removed from 12 out of 16 cohorts analysed in this study. All cohort studies were approved by the relevant institutional review boards, and conducted according to the Declaration of Helsinki. All participants of each study provided written informed consent. An overview of the study design and number of novel loci discovered at each analysis stage is available in Supplementary Fig. 54. The list of participating South Asian cohorts and corresponding sample characteristics are available in Supplementary Data 1.

### Genotyping and imputation

All study centres performed genome-wide genotyping with standard commercial genotyping platforms. Quality control (QC) of samples and genetic markers, mainly single nucleotide polymorphisms (SNPs), and imputation to 1000 Genomes (1KG) Project Phase 3 cosmopolitan reference haplotypes were conducted at each study centre^16^, except for UKBB which was imputed to the Haplotype Reference Consortium (HRC)^63^. Markers in UKBB that did not overlap with 1KG were removed from UKBB. Standard QC for samples included removing samples with low call rate (e.g. <95%), extreme heterozygosity, mismatch of sex, and those of duplicates, relatedness, and population outliers. QC for variants included removing variants with low call rate (<95%), Hardy-Weinberg equilibrium *P*-value < 1 × 10^−6^, or minor allele frequency (MAF) < 1%.

Before pre-phasing and imputation, markers were aligned to NCBI build 37 locations of the human genome using the strand files and scripts developed by the University of Oxford. A further check of variants IDs, alleles, and frequencies matching with the 1KG Phase 3 reference panel was then applied using a script developed by the University of Oxford. This stringent QC reduces errors due to strand misalignment and duplicates by removing and/or updating SNPs that do not agree in position, allele or frequency with the 1KG relevant ethnic group data. Standard pre-phasing and imputation approaches were applied including pre-phasing using SHAPEIT and imputation using IMPUTE2 or MACH/minimac locally^64–66^, or using the Michigan Imputation Server for pre-phasing and imputation. Imputation quality was examined, and allele frequencies were checked against the 1KG reference panel for South Asian population, and those significantly deviating from reference population (>0.20) were examined and any quality issue solved. All cohorts passed imputation QC. Association analyses were then performed by each study centre upon satisfactory imputation quality. In total, there were 32,869,683 SNPs available for analysis after quality control.

### Genome-wide meta-analyses

Two primary models of logistic regression association analyses were carried out with SNPTEST (for imputation with IMPUTE2), mach2dat (for imputation with MACH/minimach), or other appropriate programs (sex-combined, with or without BMI adjustment)^65,66^. Age, sex, and cohort-specific covariates were included where applicable. The first 3–15 study-specific principal components were included as covariates to correct for population substructure. Details of genotyping arrays and imputation are summarised in Supplementary Data 2.

Association summary statistics were filtered for QC before meta-analysis. Criteria for inclusion of a variant in association meta-analysis included imputation info ≥ 0.4 for IMPUTE2 and 0.3 for MACH/minimac^66–68^, minor allele count (MAC) ≥ 6, *P*-values for Hardy-Weinberg equilibrium ≥ 1 × 10^−6^, standard error (SE) > 0 and <10, and *P*-values > 0 and ≤1. Meta-analyses combining association summary statistics from all cohorts were performed using the inverse-variance weighted fixed-effect model as implemented in the METAL program^69^. Genomic control (GC) adjustment was applied in each cohort before meta-analysis when the GC factor (lambda) was greater than one.

In sex-combined models with or without BMI adjustment, SNPs with MAF ≥ 0.01, *P*-values < 1 × 10^−3^, not in a known T2D loci (>500 kb from the known variants previously published), and total sample size exceeding half of the maximum sample size, were included for further testing. This yielded a total of 18,537 and 17,929 potentially novel SNPs for the sex-combined BMI unadjusted or adjusted model at *P*-values < 1 × 10^−3^, respectively (Supplementary Data 3). The European DIAMANTE consortium data were used for the meta-analyses. Meta-analyses of summary statistics on the above SNPs in South Asians and in European DIAMANTE data were performed with METAL using the inverse-variance weighted fixed effects approach^69^. SNPs with meta-analysis *P*-values < 5 × 10^−8^ were considered statistically significant. Results are summarised in Supplementary Data 5 and 6. Genetic correlation for T2D between South Asians and Europeans were calculated using *popcorn*^30^.

### Conditional analysis

In an effort to investigate if the newly identified variants were independent of known associations, we performed a conditional regression conditional on known SNPs associated with T2D in the same loci (±1Mb) using the -cojo-cond function in the Genome-wide Complex Trait Analysis (GCTA)—conditional and joint analysis(COJO) program^70^. GCTA version 1.91.0 beta was used in this study. This approach uses the summary statistics from meta-analysis, and linkage disequilibrium data from a GWAS study or reference haplotypes, which we defined as IA610 here. Heritability for T2D was estimated using GREML in GCTA using the same cohort (IA610) as reference.

### Detection of distinct association signals

To detect multiple distinct association signals at each novel locus (+/−1Mb region), we performed approximate conditional analyses using GCTA with genome-wide meta-analysis summary statistics from South Asian cohorts in BMI-adjusted or unadjusted models, and with LD estimated from IA610. The cojo-slct option was used for a stepwise model selection to select independently associated SNPs in each region. We used cojo-p 1e-5 to set the threshold p-value to declare a locus-wide significant hit. The default multiple regression R2 (0.9) on the selected SNPs was used for the cut off value to control collinearity. SNPs with allele frequency differences >0.2 between association SNP and the reference SNP were removed from analysis.

### Cross-ancestry comparison

We then systematically investigated the association with T2D for these 21 novel loci in other ethnic-specific studies, including participants of East Asian, African-American and Hispanic ancestry. Sentinel SNPs were looked up in both BMI-unadjusted and adjusted models where possible. Results are shown in Supplementary Data 10 and 11.

For East Asians, the T2D meta-analyses were performed with studies participating in the Asian Genetic Epidemiology Network (AGEN), a consortium of genetic epidemiology studies of T2D and related traits conducted in individuals of East Asian ancestry. The first phase of the East Asian meta-analysis included 77,418 T2D cases and 356,122 controls from 20 GWAS and three biobanks, China Kadoorie Biobank (CKB), Korea Biobank Array (KBA), and Biobank Japan (BBJ) (*N*_eff_ = 211,793)^71–74^. In Phase 2 of the meta-analysis, a subset from Phase 1 (56,267 T2D cases and 227,155 controls; *N*_eff_ = 139,701) were analysed in BMI-adjusted and sex-specific models. Included studies were genotyped on either commercially available or customised Affymetrix or Illumina genome-wide genotyping arrays. Array quality control criteria implemented within each study, including variant call rate and Hardy-Weinberg equilibrium. The genotype scaffold for each study was then imputed to the 1000 Genomes Phase 1 or 3 reference panel using minimac3 or IMPUTEv2^16,67,75,76^. In Phase 1, all studies were imputed to 1000 Genomes Phase 3. In Phase 2, all studies were imputed to 1000 Genomes Phase 3 except for Biobank Japan (BBJ), which was imputed to the 1000 Genomes Phase 1 reference panel^76^. Institutional review boards approved all study protocols at their respective sites, and written informed consent was obtained from all participants.

Data of African–American ancestry was based upon the MEDIA (Meta-analysis of Type 2 Diabetes in African Americans) Consortium, a collaborative effort to combine T2D GWAS data from individuals of African ancestry. The current study includes 18 GWAS cohorts (15,043 T2D cases and 22,318 controls), with genotype data imputed to either 1000 Genomes phase 1 or phase 3.

For the Hispanic/Latino ancestry meta-analyses, 14 studies with a total of 13,151 cases and 21,511 controls were included. The component studies consisted of the Bio*Me* Biobank^77^, the Genetics of Latinos Diabetic Retinopathy study^78^, Hispanic Community Health Study and Study of Latinos^79^, the Mexican-American Hypertension-Insulin Resistance Family Study^80–82^, Los Angeles Latino Eye Study^83^, Mexican-American Coronary Artery Disease Study^84,85^, Mexico City studies 1 and 2^86^, Multi-Ethnic Study of Atherosclerosis^87^, the Non-Insulin-Dependent Diabetes Mellitus-Atherosclerosis Study^84^, San Antonio Family Heart Study^88,89^, the Slim Initiative in Genomic Medicine for the Americas study^17,18,90^, Starr County Health Studies^91^, and the Women’s Health Initiative study^92^. All studies comprised self-reported Hispanic/Latino individuals with varying country of origin and admixture proportions. Quality control of genome-wide genotype data, imputation to 1000 Genomes Project reference datasets phase 1 or 3^16,76^, and association analyses were performed for each study. Results from association analyses adjusted for sex were provided for all studies, and when available, results from additional analyses adjusted for both sex and body mass index, and sex-stratified analyses both with and without adjustment for body mass index were provided for meta-analyses as well. Association results for each study were filtered to remove low-frequency variants (minor allele count <14) and low-quality variants (minimac3 *r*^2^ < 0.3 or IMPUTE2 info <0.4)^67,75,93^. Meta-analysis was performed using MR-MEGA^94^, including one axis of genetic variation as captured by study-level measures of mean allele frequency differences.

To check for heterogeneity between ethnic populations, we performed meta-analyses of summary statistics on the SNPs with METAL using the inverse-variance weighted fixed effects approach.

### Effects of BMI adjustment and Sex-differentiated meta-analysis

The difference between BMI-adjusted and unadjusted models was tested using a matched analysis within the same subset of 15 cohorts, where the Z-score is calculated as below to account for correlation between the two models^6^.

1 $$(\beta -{\beta }_{1})/{{{{{\rm{sqrt}}}}}}({{{{{{\rm{SE}}}}}}}^{2}-{{{{{{{\rm{SE}}}}}}}_{1}}^{2}{-}2\,{\times}\,\rho \,{\times}\,{{{{{\rm{SE}}}}}}\,{\times}\,{{{{{{\rm{SE}}}}}}}_{1}),$$

where β and β_1_ are effect sizes for BMI-unadjusted and adjusted models, respectively, SE and SE_1_ are the corresponding standard errors, and ρ is the estimated correlation between β and β_1_ obtained from all SNPs from this study (ρ = 0.96).

Sex-differentiated meta-analysis allowing for heterogeneity of allelic effects between males and females was conducted to detect sex-differentiated effects with the GWAMA program^95^.

### Expression quantitative trait loci (eQTL) analysis

Using eQTL data generated from whole blood gene expression data from 31,684 individuals by the eQTLGen Consortium^33^, we examined the *cis*-associations of the novel variants and their proxies (*r*^2^ ≥ 0.8 in South Asians) with expression levels of nearby genes (±1 Mb) in all tissues to explore the potential functionality of novel variants. Additional eQTL lookups were performed for (i) whole blood in South Asians from LOLIPOP (*n* = 693) and (ii) in pancreatic islets (*n* = 420) to explore metabolically relevant tissue-specific relationships^96^. We also performed permutation testing to determine the extent of enrichment of *cis* eQTLs observed. We first determined the number of our novel sentinel SNPs that showed significant *cis* eQTLs. Expectation under the null hypothesis was then determined by permutation testing using a set of matched background SNPs. SNPs were matched for MAF (±2% in South Asians) and distance to the nearest gene (±10 kb). *P*-values were calculated by comparing the observed number of SNPs that displayed significant *cis* eQTLs to the mean expectation under the null hypothesis from permutation testing using a 1-sample *t* test (*N* = 1000 permutations of 21 SNPs).

### Expression quantitative trait loci (eQTL) analysis in South Asians

Transcriptome-wide measurements of gene expression in blood were undertaken in South Asian (*N* = 693) participants of the LOLIPOP study, using the Illumina HumanHT-12 v4 BeadChip array according to manufacturer’s protocol. Expression values were summarised to gene level estimates by averaging the log2 transformed expression levels of probes mapping to the same gene. To quantify the relationship between genetic variation and gene expression we first derived residuals for gene expression using linear regression of gene expression levels against sex, age, RNA integrity number, RNA conversion batch and RNA extraction batch. Expression residuals were then used as outcome variables in a linear regression model with SNP dosage as the independent variable, corresponding to the following linear model formulae: Gene ~ SNP + sex + age + RIN + RNA_Conv_Batch + RNA_Extract_Batch. Data analysis was performed using Matrix eQTL^97^.

### methQTL analysis

For the discovery phase, we measured DNA methylation in 1,841 South Asians using peripheral blood sample collected at baseline from the London Life Sciences Prospective Population Study (LOLIPOP) (see Cohort Description—Supplementary Note 1); for the replication phase we studied 1354 South Asians using blood sample collected at the follow-up visit. All participants were unrelated. DNA methylation was quantified using the Illumina HumanMethlation450K array according to the manufacturer’s instructions. Bisulfite conversion of genomic DNA was performed using the EZ DNA methylation kit according to the manufacturer’s instructions (Zymo Research, Orange, CA). Bead intensity was retrieved using the *minfi* software package, and a detection *P* value of <10^−16^ was used for marker calling. Of the 486,427 positions assayed by the array, we excluded markers with call rates <98% (*N* = 4,684), or that do not measure methylation at CpG sites (*N* = 4006). This left 466,186 autosomal and 11,329 sex chromosome markers for analysis. Markers reported to be cross-hybridising were retained but flagged. Samples with gender inconsistency and/or with low marker call rate (<98%) were filtered^98^. Genotyping was done with a combination of Illumina genotyping arrays (HumanHap300, Human-Hap610, OmniExpress and OmniExomeExpress). Genotypes were called with Illumina Genome Studio and imputation performed using the IMPUTEv2 software package and 1000 Genomes Project cosmopolitan reference panel (ALL_1000G_phase1integrated_v3_impute_macGT1). Standard GWAS quality control criteria were applied, including filtering for call-rate, minor allele frequency, info score and Hardy–Weinberg equilibrium.

Data normalisation was performed separately within each cohort and percentage methylation at each CpG site was calculated. Residuals were then derived from a linear regression of the percentage methylation (outcome) with technical and clinical predictors. These include age, sex, estimates of white-blood cell subpopulations and principal components of control-probe intensities. Residuals were used as outcomes in association testing. Association testing for the discovery stage was performed separately for each study population and genotyping platform using QuickTest. Summary statistics were combined by fixed-effect meta-analysis using METAL^69^. Statistical significance was set to *P* < 7.7 × 10^−6^, which corresponds to *P* < 0.05 after Bonferroni correction for the 6,494 SNP-CpG association tests.

We also performed permutation testing to determine the extent of enrichment of *cis* methQTLs observed. We first determined the number of our novel sentinel SNPs that showed significant *cis* methQTLs. Expectations under the null hypothesis was then determined by permutation testing using a set of matched background SNPs. SNPs were matched for MAF (±2% in South Asians) and distance to the nearest gene (±10 kb). *P*-values were calculated by comparing the observed number of SNPs that displayed significant *cis* methQTLs to the mean expectation under the null hypothesis from permutation testing using a 1-sample t-test (*N* = 1,000 permutations of 21 SNPs).

### Colocalization analysis

We used *coloc* 2.3-1 to check for colocalization of the T2D association signals at our 21 loci with eQTL signals from eQTLgen^99^. For each locus, we examined all SNPs available in both datasets within 1 Mb of the sentinel SNP identified in our T2D GWAS, and ran *coloc.abf* with default parameters and priors. The summary statistics from the joint meta-GWAS of South Asians and Europeans were used for the SNP-T2D axis. We also checked for colocalization between eQTL and methQTL associations. We considered there to be sufficient evidence for colocalization when *coloc* PP4 > 0.6 (posterior probability for shared underlying causal variant).

### Functional annotation and cross-trait associations

We annotated the sentinel SNPs and their proxies for regulatory regions (promoter and enhancer histone marks, DNase I hypersensitivity, protein binding and regulatory motifs) with HaploRegv4.1 (Broad Institute) using the *haploR* package in R (version 3.6.0)^38^. VEP (Variant Effect Predictor) was used for the identification of transcription factor binding sites and nonsynonymous and splicing variants^37^. EpiExplorer and the UCSC Genome Browser were used to annotate CpG islands^100^. We performed permutation testing to determine the degree of enrichment for various regulatory regions. We first determined the number of novel sentinel SNPs that were annotated for the respective features, followed by generating expectations under the null hypothesis by permutation testing using a set of matched background SNPs. SNPs are matched for MAF (±2% in South Asians) and distance to the nearest gene (±10 kb). *P*-values were calculated by comparing the observed number of SNPs that were annotated for the respective features to the mean expectation under the null hypothesis from permutation testing using a 1-sample t-test (*N* = 1000 permutations of 21 SNPs).

### Polygenic risk score

For PRS derivation, we applied the LDpred algorithm, a Bayesian approach that calculates posterior mean effect for all variants based on a prior (effect size and level of statistical significance in the prior GWAS) and subsequent shrinkage based on linkage disequilibrium (LD) with other variants nearby^49^. DNA polymorphisms with ambiguous strand (A/T or C/G) were removed from the score derivation.

We developed two models for PRS derivation, one based upon South Asian-derived summary statistics (current study) and another based upon European-derived summary statistics for T2D associations (DIAMANTE). For the South Asian-derived summary statistics, we reperformed our genome-wide meta-analysis across only 14 cohorts, excluding two cohorts to be utilised as validation and independent testing series for LDpred (validation: IA610; *n* = 2,019 cases and 3696 controls and testing: SINDI: *n* = 974 cases and 1168 controls). A third independent cohort was also used for testing (LOLIPOP_GSA: *n* = 900 cases and 919 controls). South Asians and Europeans from the 1000 Genomes Phase 3 were used as the reference panel for LD calculation for the South Asian- and European-derived PRS models, respectively.

LD radius was set to M/3000 (default value in LDpred) in both cases, whereby M is the total number of SNPs used in the corresponding analysis. For the tuning parameter p, which refers to the proportion of variants assumed to be causal (non-zero effects), a range of values were tested (1, 0.3, 0.1, 0.03, 0.01, 0.003, 0.001, 0.0003, 0.0001) and the SNP weights generated used to calculate PRS. The optimal PRS for the South Asian-derived summary statistics model was chosen according to the area under the receiver-operator curve (AUC) based upon a logistic regression model^47^. For the European-derived summary statistics model, we selected the model with *p* = 0.01, as previously reported to be optimum^47^. The proportion of variance explained was calculated for each model using Nagelkerke’s pseudo-R2 metric. To plot the risk gradient, we first divided the validation population into 10 bins according to decile of the PRS, and plotted the prevalence of T2D within each bin.

### Reporting summary

Further information on research design is available in the Nature Research Reporting Summary linked to this article.

## Supplementary information

Supplementary Information

Description of Additional Supplementary Files

Supplementary Data 1-26

Reporting Summary

## Data Availability

Summary statistics data is available via GWAS catalogue. Study Accession IDs are GCST90093109 and GCST90093110 for the BMI-unadjusted and BMI-adjusted models, respectively. Full summary statistics for *cis* eQTL analyses were downloaded from eQTLGen website (https://eqtlgen.org/cis-eqtls.html).
