# Author Correction: Identification of genetic effects underlying type 2 diabetes in South Asian and European populations

**DOI:** 10.1038/s42003-022-03404-x

**Published:** 2022-05-05

**Authors:** Marie Loh, Weihua Zhang, Hong Kiat Ng, Katharina Schmid, Amel Lamri, Lin Tong, Meraj Ahmad, Jung-Jin Lee, Maggie C. Y. Ng, Lauren E. Petty, Cassandra N. Spracklen, Fumihiko Takeuchi, Md. Tariqul Islam, Farzana Jasmine, Anuradhani Kasturiratne, Muhammad Kibriya, Karen L. Mohlke, Guillaume Paré, Gauri Prasad, Mohammad Shahriar, Miao Ling Chee, H. Janaka de Silva, James C. Engert, Hertzel C. Gerstein, K. Radha Mani, Charumathi Sabanayagam, Marijana Vujkovic, Ananda R. Wickremasinghe, Tien Yin Wong, Chittaranjan S. Yajnik, Salim Yusuf, Habibul Ahsan, Dwaipayan Bharadwaj, Sonia S. Anand, Jennifer E. Below, Michael Boehnke, Donald W. Bowden, Giriraj R. Chandak, Ching-Yu Cheng, Norihiro Kato, Anubha Mahajan, Xueling Sim, Mark I. McCarthy, Andrew P. Morris, Jaspal S. Kooner, Danish Saleheen, John C. Chambers

**Affiliations:** 1 Lee Kong Chian School of Medicine, Nanyang Technological University, Singapore, 308232 Singapore; 2 Department of Epidemiology and Biostatistics, Imperial College London, London, W2 1PG UK; 3 Department of Cardiology, Ealing Hospital, London North West Healthcare NHS Trust, Middlesex, UB1 3HW UK; 4 Institute of Computational Biology, Deutsches Forschungszentrum für Gesundheit und Umwelt, Helmholtz Zentrum München, 85764 Neuherberg, Germany; 5 Department of Informatics, Technical University of Munich, 85748 Garching bei München, Neuherberg, Germany; 6 Department of Medicine, McMaster University, Hamilton, ON Canada; 7 Population Health Research Institute, Hamilton Health Sciences and McMaster University, Hamilton, ON Canada; 8 The University of Chicago, Biological Sciences Division, Public Health Sciences, 5841 South Maryland Avenue, MC2000, Chicago, IL 60637 USA; 9 Genomic Research on Complex diseases, CSIR-Centre for Cellular and Molecular Biology (CSIR-CCMB), Hyderabad, India; 10 Translational Medicine and Human Genetics, Department of Medicine, University of Pennsylvania, Philadelphia, Pennsylvania USA; 11 Department of Medicine, Mayo Hospital, Lahore, Pakistan; 12 Center for Genomics and Personalized Medicine Research, Center for Diabetes Research, Wake Forest School of Medicine, Winston-Salem, NC 37215 USA; 13 Vanderbilt Genetics Institute, Division of Genetic Medicine, Vanderbilt University Medical Center, Nashville, TN 37232 USA; 14 Human Genetics Center, School of Public Health, The University of Texas Health Science Center at Houston, Houston, TX 77030 USA; 15 Department of Genetics, University of North Carolina at Chapel Hill, Chapel Hill, NC 27599 USA; 16 Department of Biostatistics and Epidemiology, University of Massachusetts, Amherst, MA 01003 USA; 17 Department of Gene Diagnostics and Therapeutics, Research Institute, National Center for Global Health and Medicine, Tokyo, Japan; 18 U Chicago Research Bangladesh, House#4, Road#2b, Sector#4, Uttara, Dhaka, 1230 Bangladesh; 19 Department of Public Health, Faculty of Medicine, University of Kelaniya, Kelaniya, Sri Lanka; 20 Department of Pathology and Molecular Medicine, McMaster University, Hamilton, ON Canada; 21 Academy of Scientific and Innovative Research, CSIR-Institute of Genomics and Integrative Biology Campus, New Delhi, 110020 India; 22 Systems Genomics Laboratory, School of Biotechnology, Jawaharlal Nehru University, New Delhi, 110067 India; 23 Singapore Eye Research Institute, Singapore National Eye Centre, Singapore, Singapore; 24 Department of Medicine, Faculty of Medicine, University of Kelaniya, Kelaniya, Sri Lanka; 25 Department of Medicine, McGill University, Montreal, QC Canada; 26 Department of Human Genetics, McGill University, Montreal, QC Canada; 27 Department of Health Research Methods, Evidence, and Impact, McMaster University, Hamilton, ON Canada; 28 Ophthalmology & Visual Sciences Academic Clinical Program (Eye ACP), Duke-NUS Medical School, Singapore, Singapore; 29 Department of Biostatistics and Epidemiology, University of Pennsylvania, Philadelphia, Pennsylvania 19104 USA; 30 Department of Ophthalmology, Yong Loo Lin School of Medicine, National University of Singapore, Singapore, Singapore; 31 Diabetes Unit, King Edward Memorial Hospital and Research Centre, Pune, India; 32 Department of Biostatistics and Center for Statistical Genetics, University of Michigan, Ann Arbor, Michigan 48109 USA; 33 Department of Biochemistry, Wake Forest School of Medicine, Winston-Salem, NC 37215 USA; 34 JSS Academy of Health Education of Research, Mysuru, India; 35 Science and Engineering Research Board, Department of Science and Technology, Ministry of Science and technology, Government of India, New Delhi, India; 36 Oxford Centre for Diabetes, Endocrinology and Metabolism, Radcliffe Department of Medicine, University of Oxford, Oxford, OX3 7BN UK; 37 Wellcome Centre for Human Genetics, Nuffield Department of Medicine, University of Oxford, Oxford, OX3 7BN UK; 38 Saw Swee Hock School of Public Health, National University of Singapore, Singapore, Singapore; 39 Oxford NIHR Biomedical Research Centre, Churchill Hosptial, Oxford University Hospitals NHS Foundation Trust, Oxford, OX3 7LE UK; 40 Department of Biostatistics, University of Liverpool, Liverpool, L69 3GL UK; 41 Estonian Genome Center, Institute of Genomics, University of Tartu, Tartu, Estonia; 42 Wellcome Trust Centre for Human Genetics, University of Oxford, Oxford, OX3 7BN UK; 43 Imperial College Healthcare NHS Trust, Imperial College London, London, W12 0HS UK; 44 MRC-PHE Centre for Enviroment and Health, Imperial College London, London, W2 1PG UK; 45 National Heart and Lung Institute, Imperial College London, London, W12 0NN UK; 46 Center for Non-Communicable Diseases, Karachi, Pakistan; 47 Department of Medicine, Columbia University Irving Medical Center, New York, NY 10032 USA; 48 Department of Cardiology, Columbia University Irving Medical Center, New York, NY 10032 USA

**Keywords:** Genetic variation, Diabetes

Correction to: *Communications Biology* 10.1038/s42003-022-03248-5, published online 7 April 2022.

In this article the citation number 57 was incorrectly given as Silverman, S. H., Purdue, G. F., Hunt, J. L. & Bost, R. O. Cyanide toxicity in burned patients. *J. Trauma.* **28**, 171–176 (1988), but should have been Herdenberg, C., Mutie, P.M., Billing, O. et al. LRIG proteins regulate lipid metabolism via BMP signaling and affect the risk of type 2 diabetes. *Commun. Biol.* **4**, 90 (2021). 10.1038/s42003-020-01613-w.

In the Discussion sentence beginning ‘Indeed, it has been recently shown…’ in this article, the text 'LRIG' should read '*LRIG1*'.

These have now been corrected in the PDF and HTML versions of the Article.

