# Supplementary material for: Identification of genetic effects underlying type 2 diabetes in South Asian and European populations
Source: Commun Biol. 2022 Apr 7;5:329. doi: 10.1038/s42003-022-03248-5 (PMC8991226; doi:10.1038/s42003-022-03248-5)
Supplement: Supplementary file 1 — Supplementary Information [file 42003_2022_3248_MOESM1_ESM.pdf]

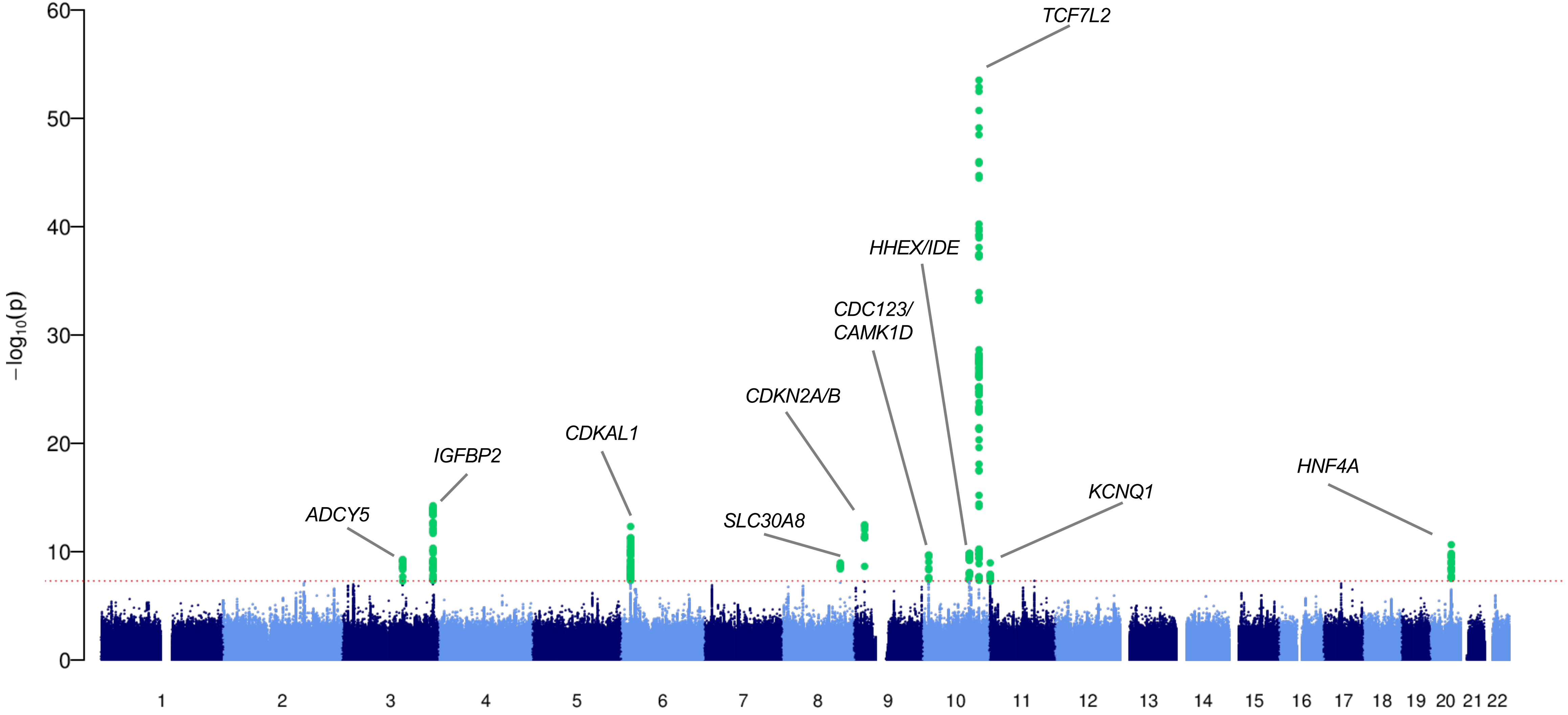

Supplementary Figure 1. Manhattan plot for genome-wide association with T2D in meta-analysis of South Asian cohorts with BMI unadjusted model. Significant loci are coloured in green.

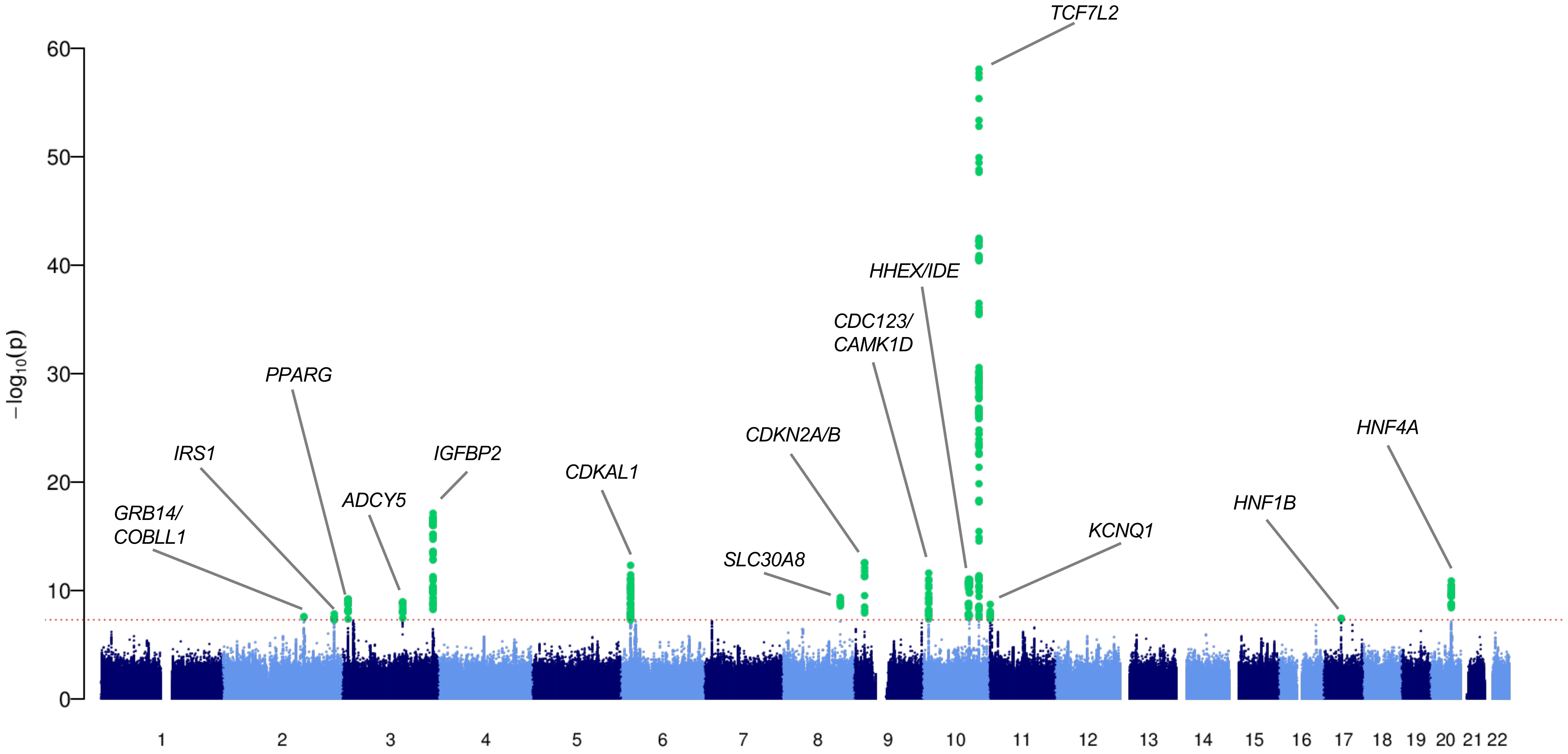

Supplementary Figure 2. Manhattan plot for genome-wide association with T2D in meta-analysis of South Asian cohorts with BMI unadjusted model. Significant loci are coloured in green.

rs10916784 (chr1: 20729451)

South Asians only

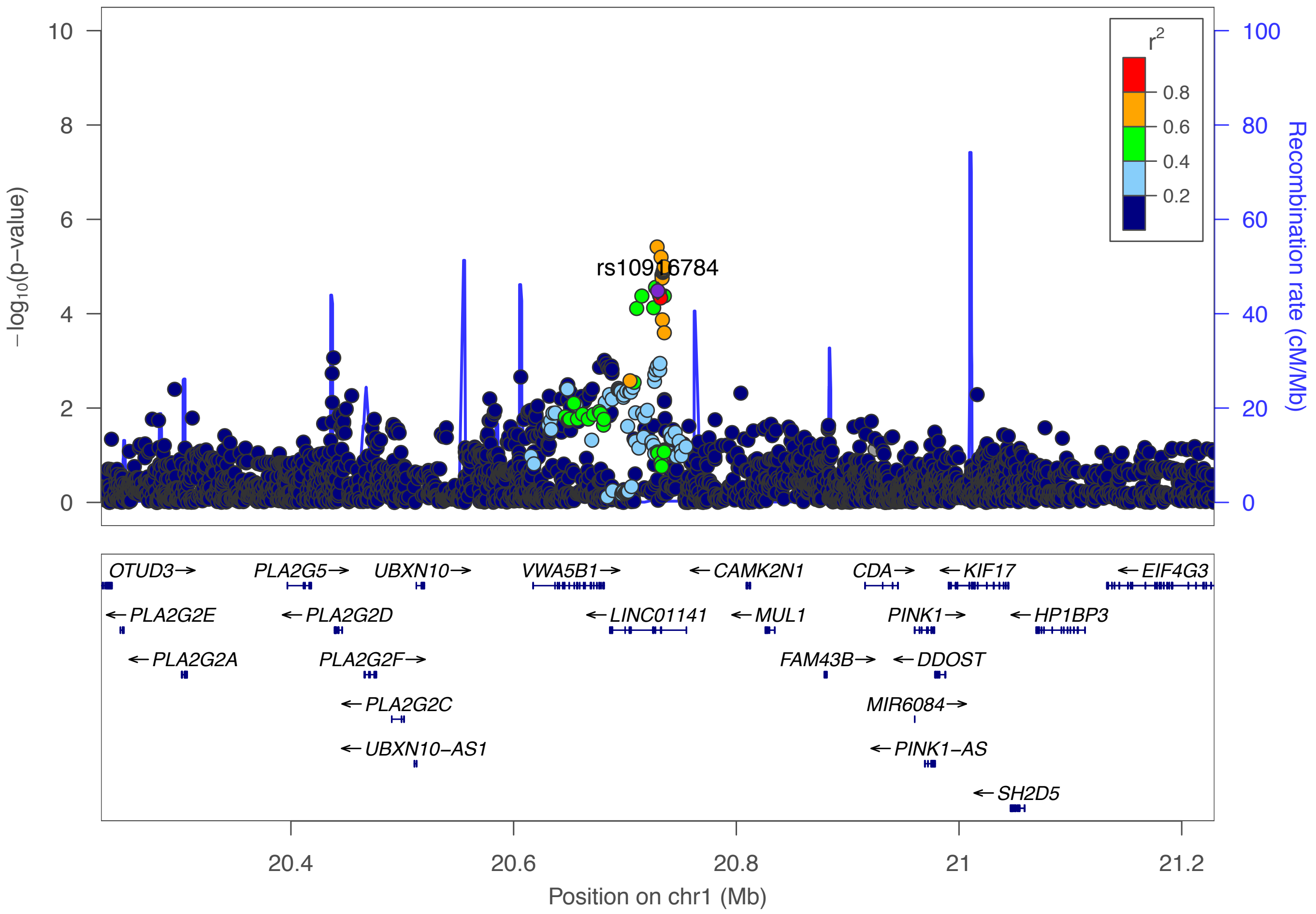

South Asians + Europeans

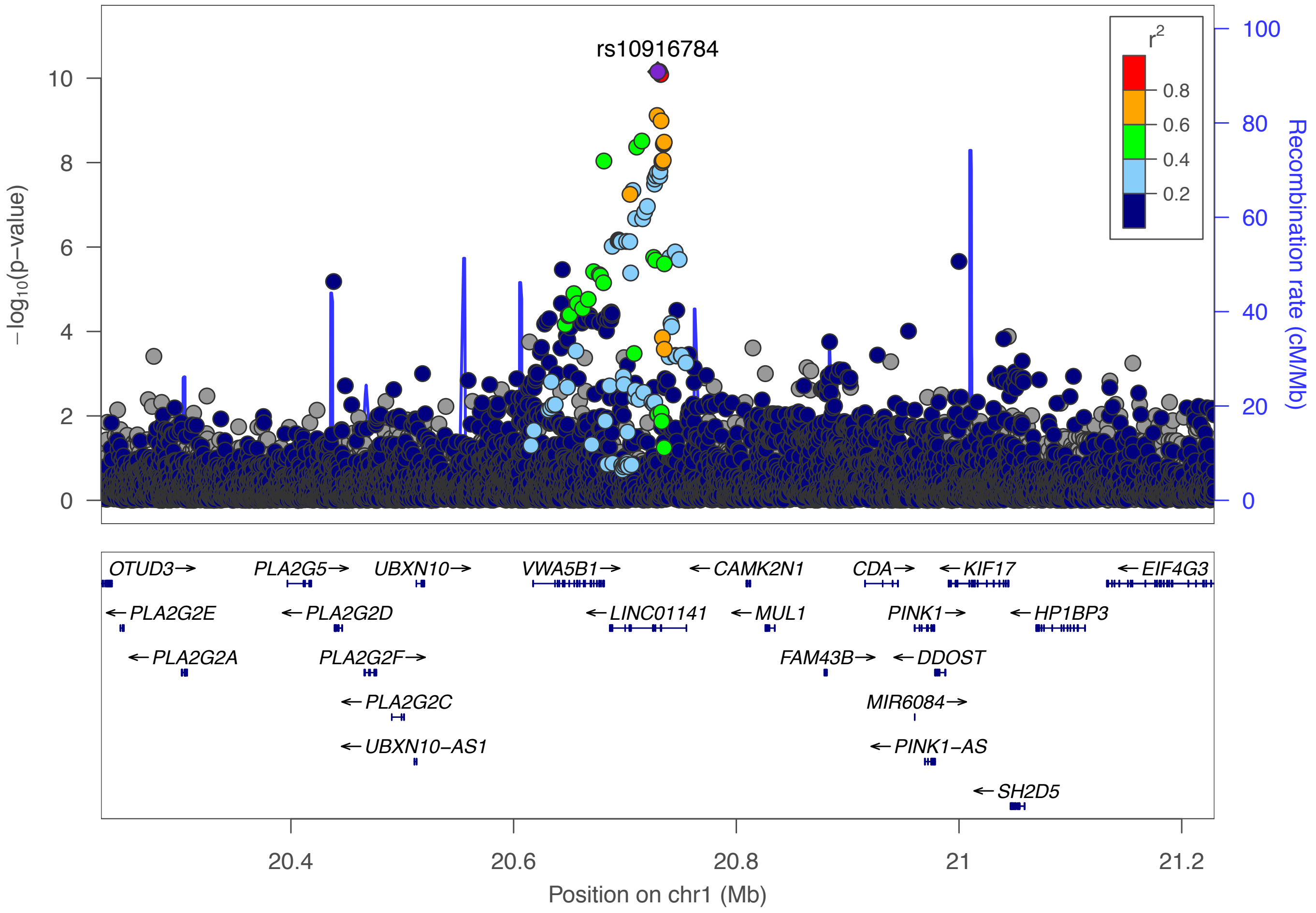

Supplementary Figure 3. Regional plots for rs10916784, showing association of SNPs with T2D for BMI unadjusted model in South Asians only, and in South Asians and Europeans.

rs7531962 (chr1: 146746023)

South Asians only

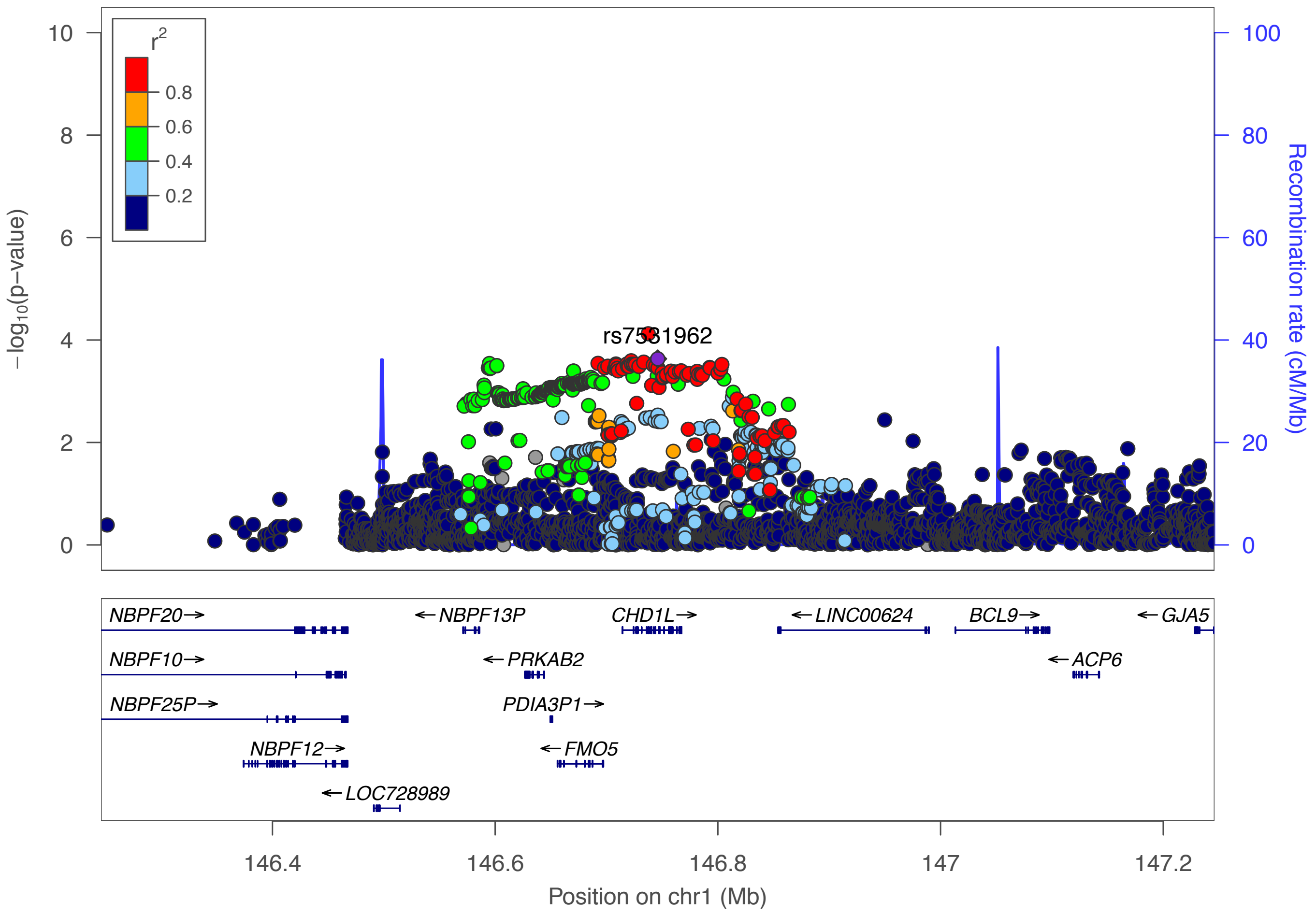

South Asians + Europeans

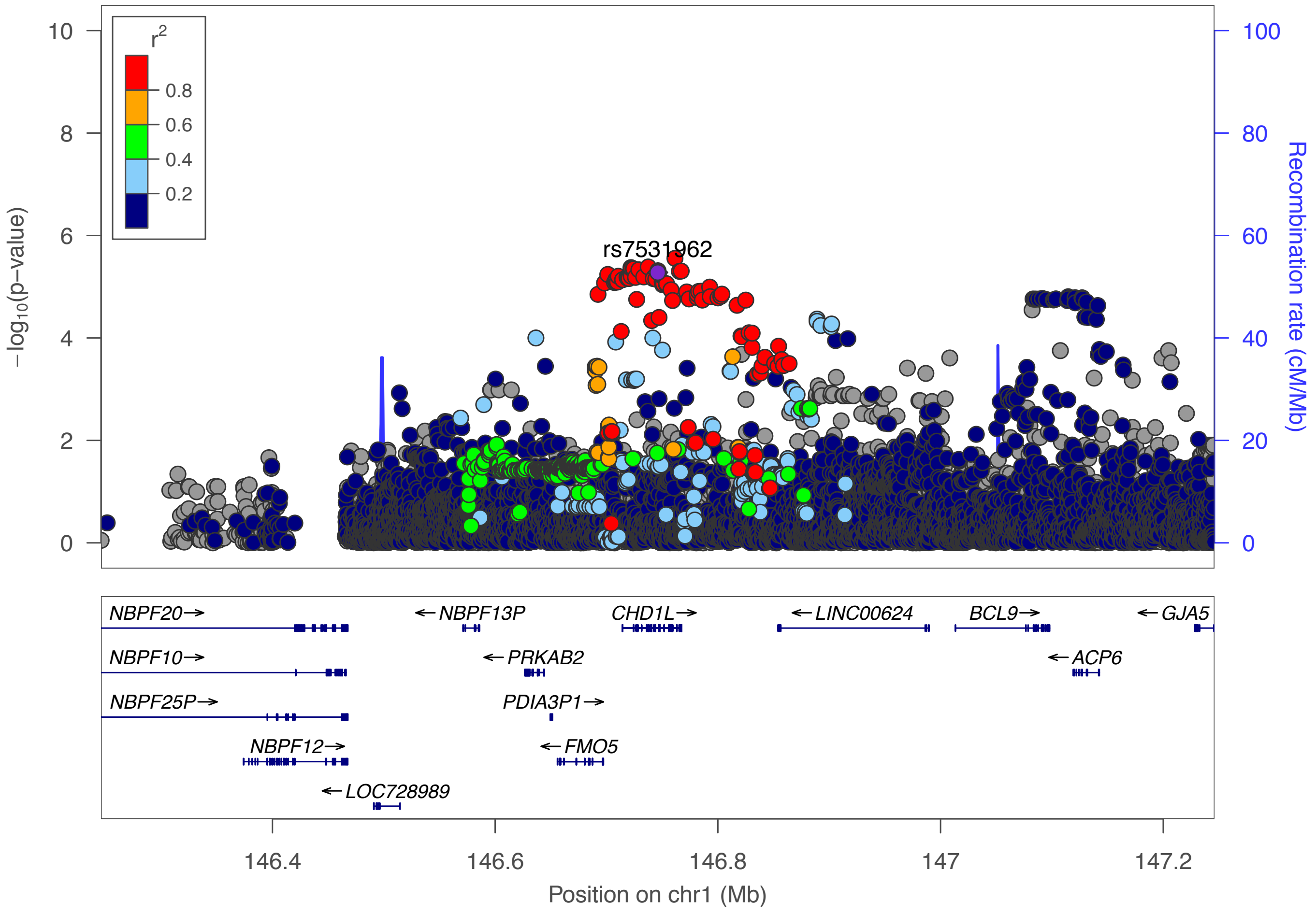

Supplementary Figure 4. Regional plots for rs7531962, showing association of SNPs with T2D for BMI unadjusted model in South Asians only, and in South Asians and Europeans.

rs12463719 (chr2: 203450680)

South Asians only

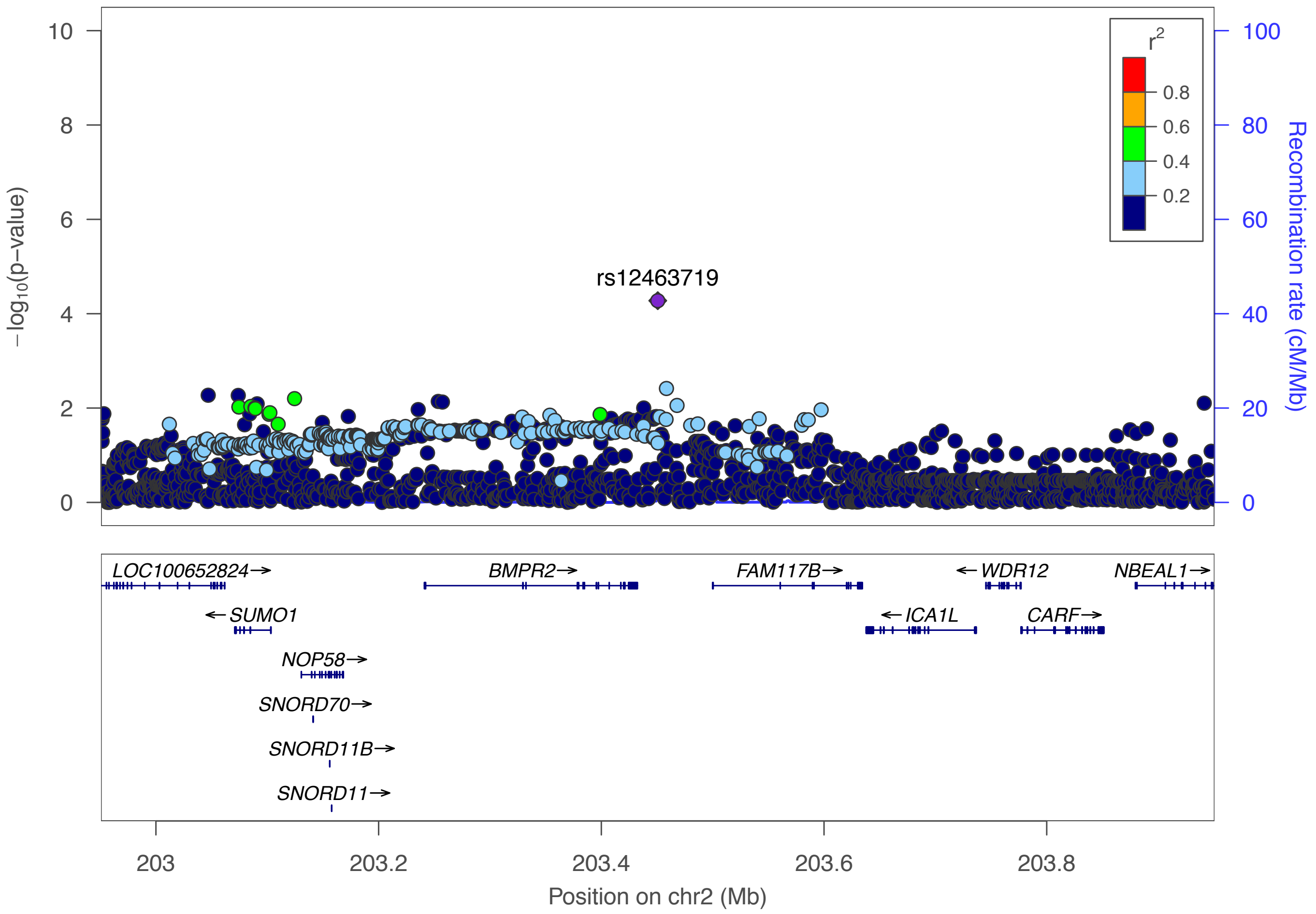

South Asians + Europeans

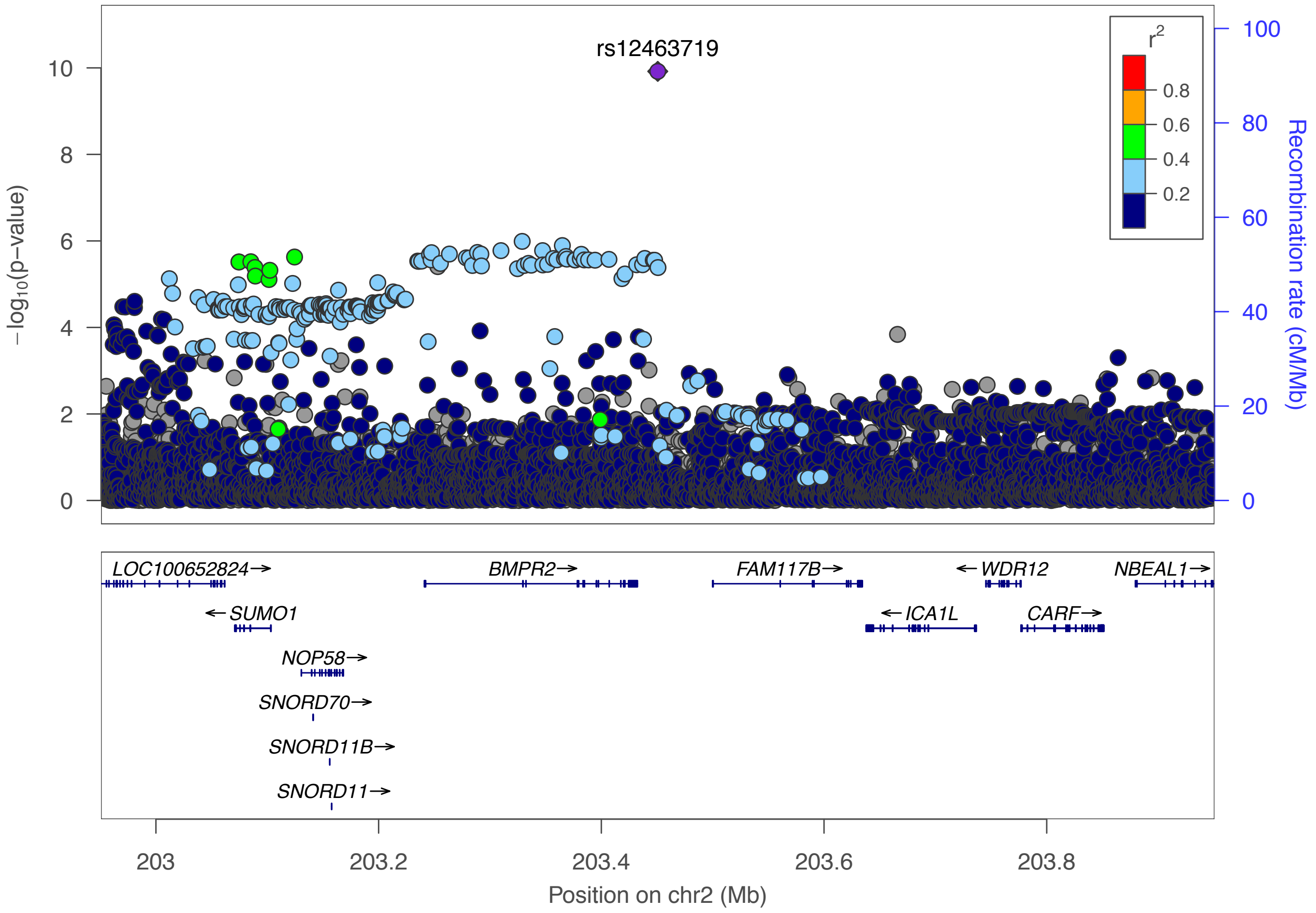

Supplementary Figure 5. Regional plots for rs12463719, showing association of SNPs with T2D for BMI unadjusted model in South Asians only, and in South Asians and Europeans.

rs7432739 (chr3: 58364650)

South Asians only

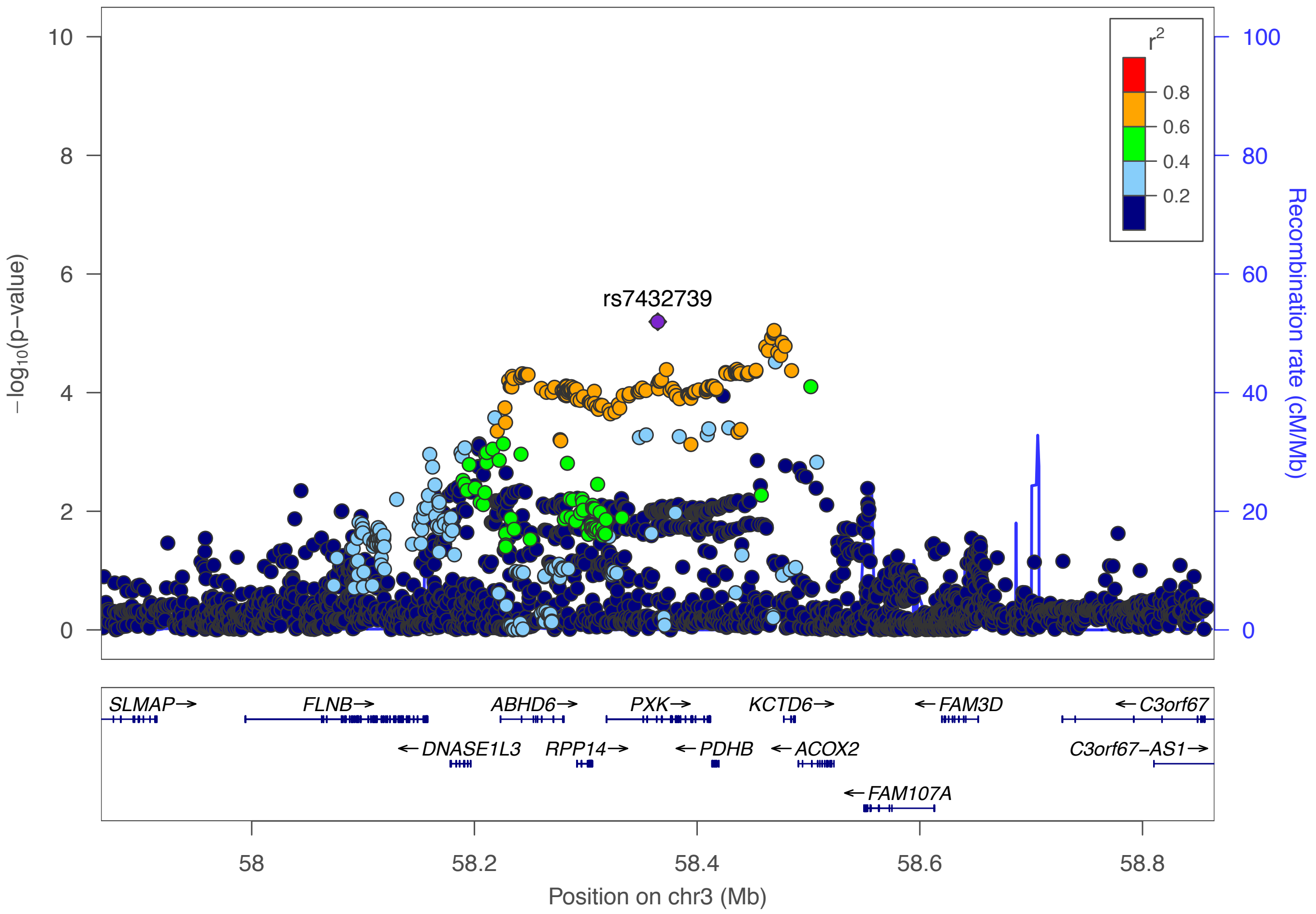

South Asians + Europeans

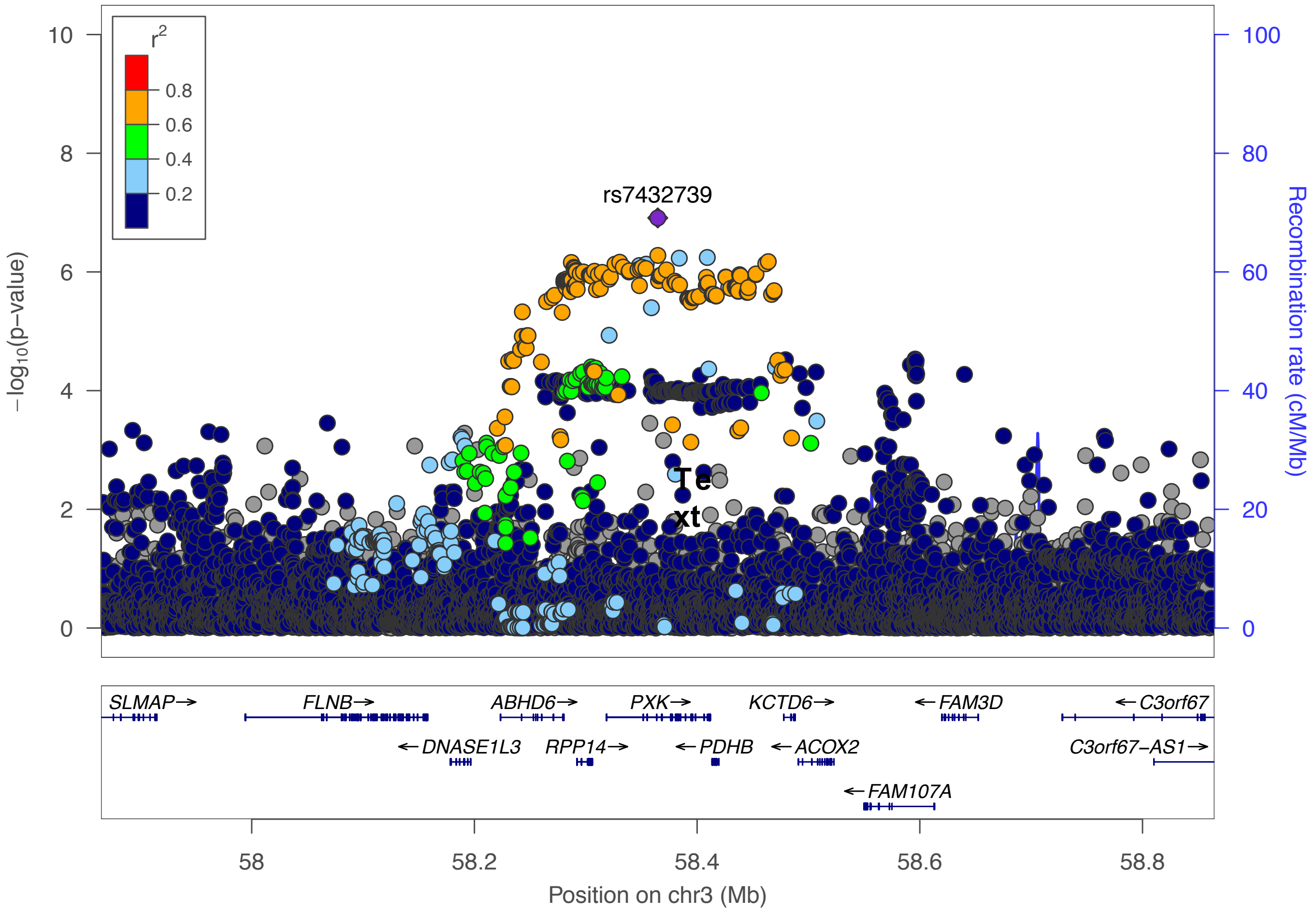

Supplementary Figure 6. Regional plots for rs7432739, showing association of SNPs with T2D for BMI unadjusted model in South Asians only, and in South Asians and Europeans.

rs7626079 (chr3: 66427259)

South Asians only

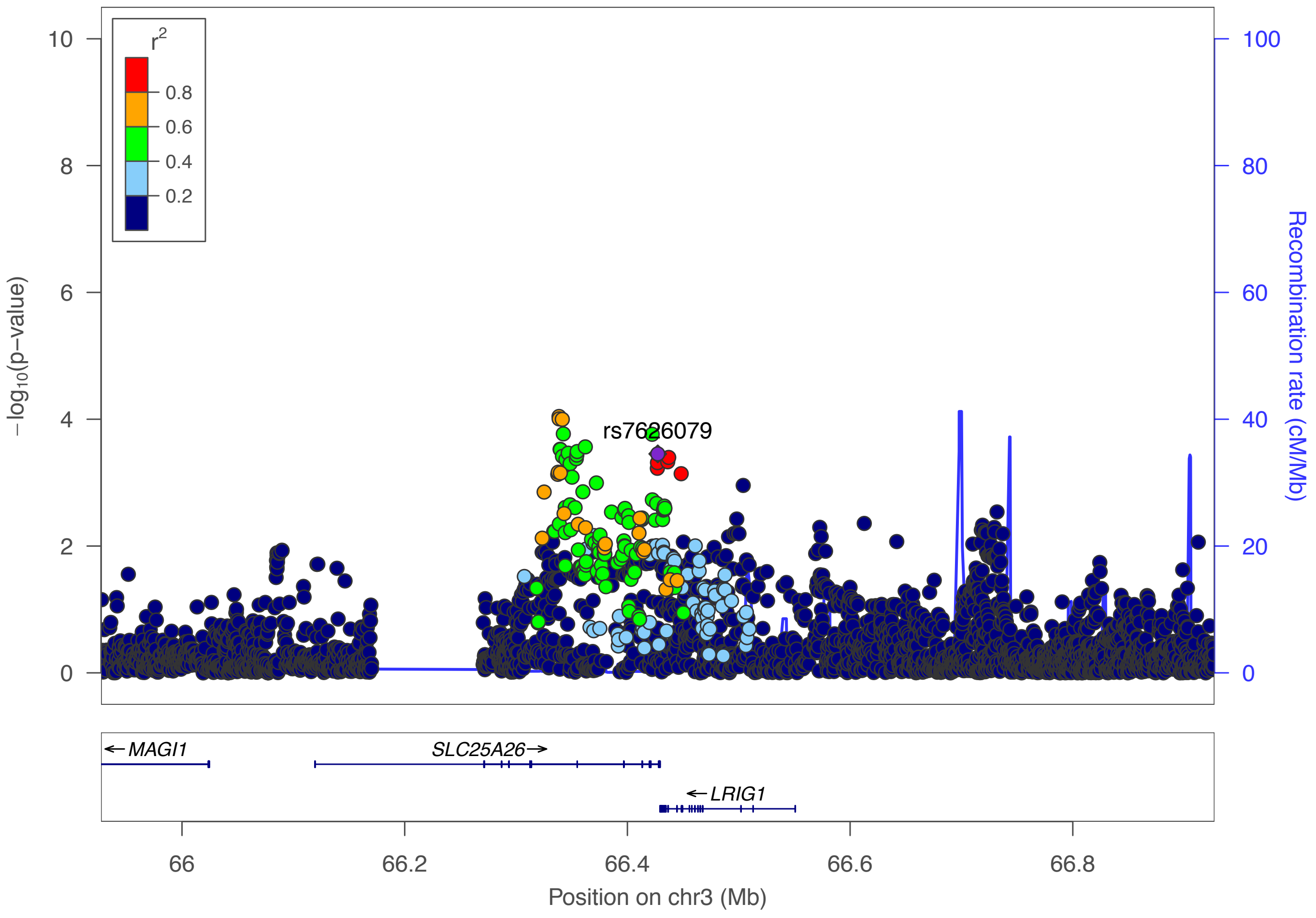

South Asians + Europeans

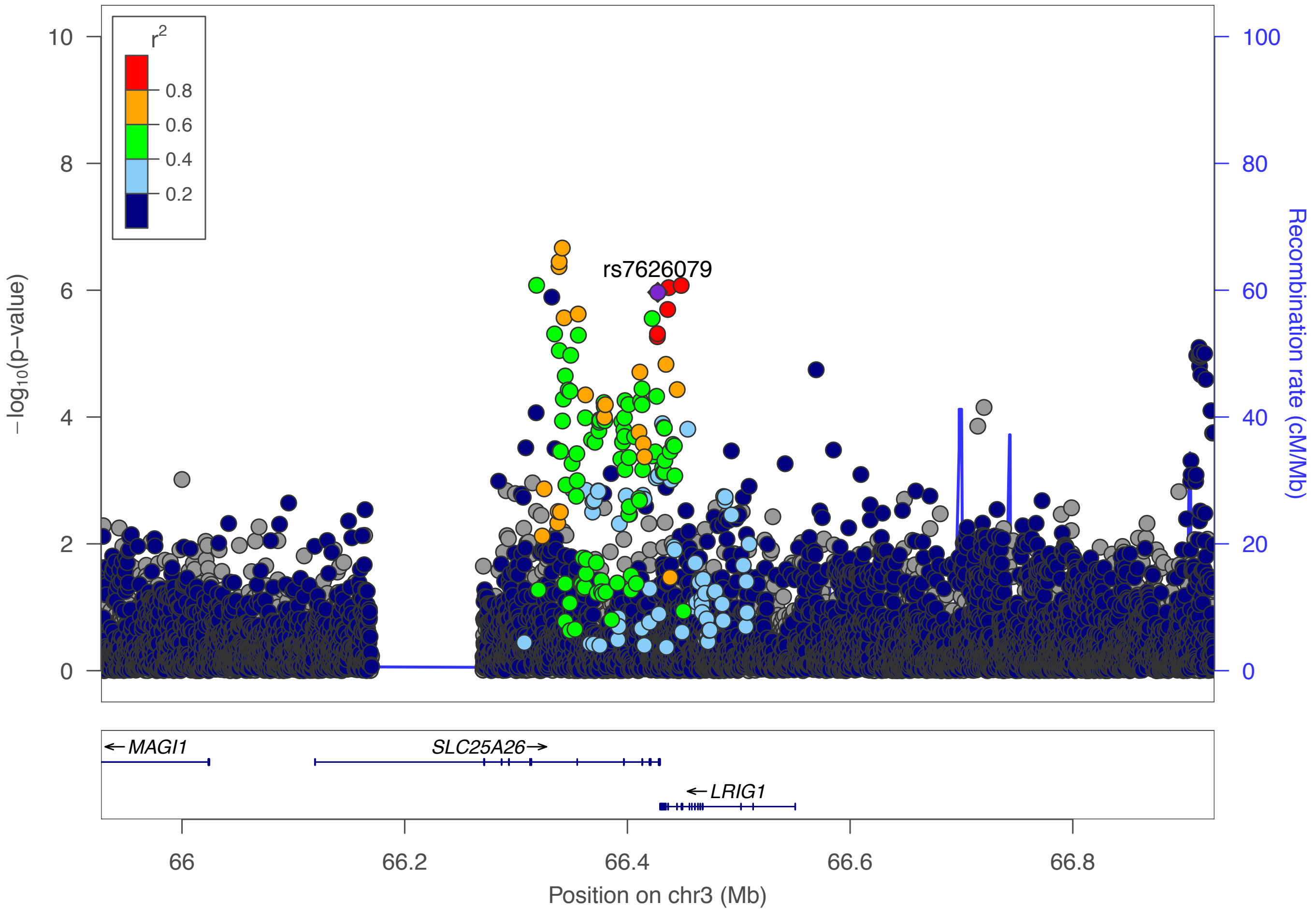

Supplementary Figure 7. Regional plots for rs7626079, showing association of SNPs with T2D for BMI unadjusted model in South Asians only, and in South Asians and Europeans.

rs62366901 (chr5: 49930286)

South Asians only

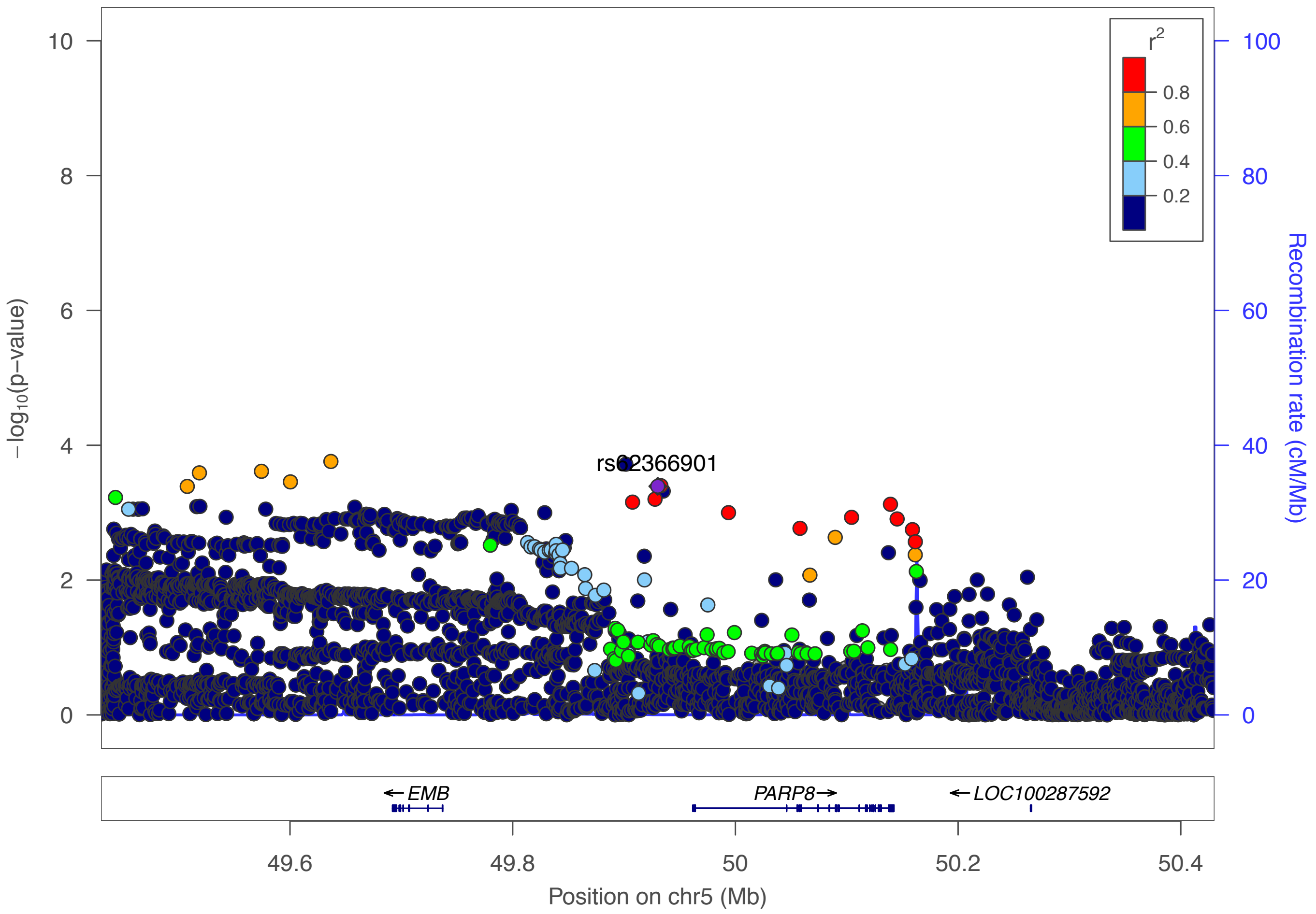

South Asians + Europeans

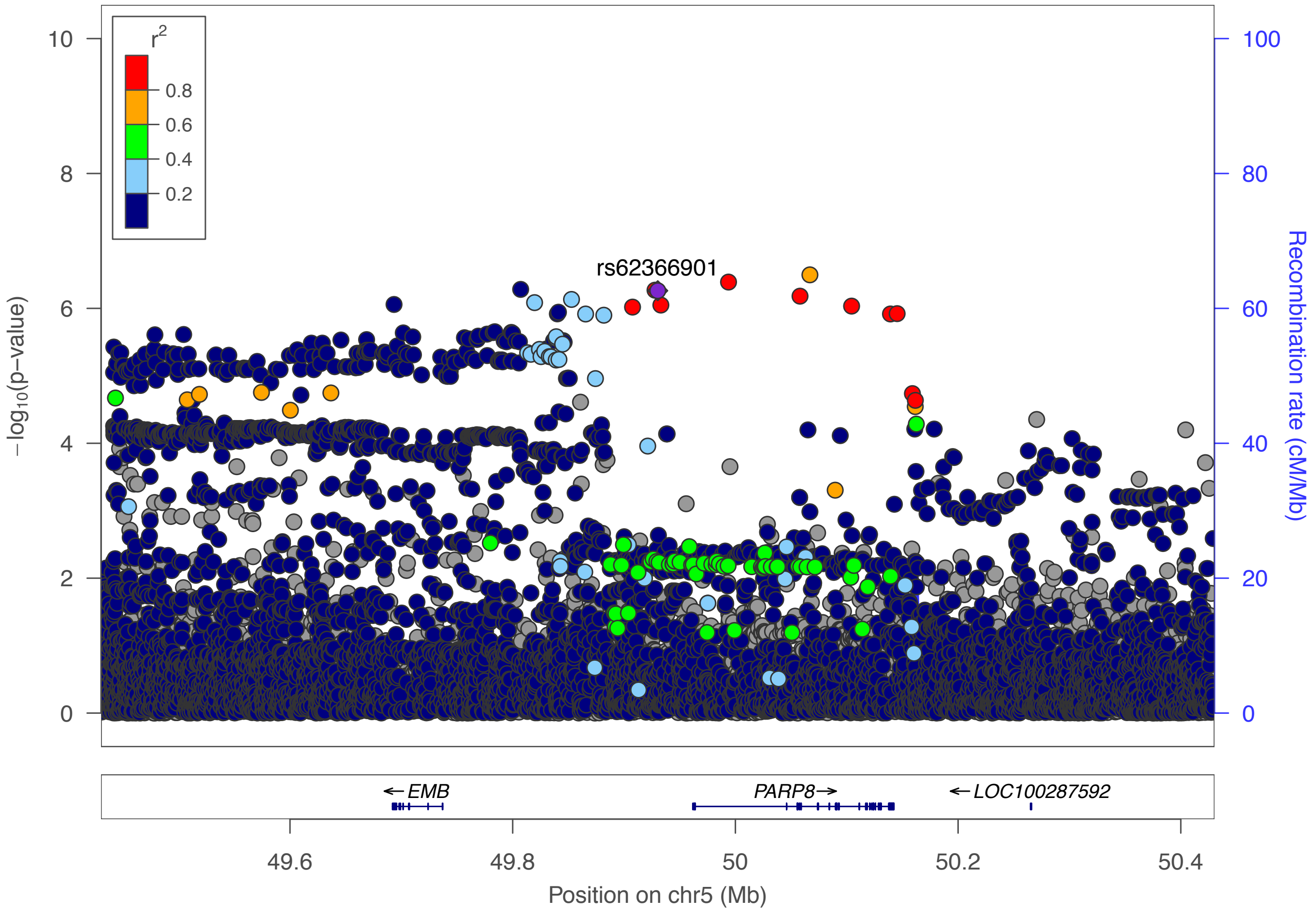

Supplementary Figure 8. Regional plots for rs62366901 showing association of SNPs with T2D for BMI unadjusted model in South Asians only, and in South Asians and Europeans.

rs74790763 (chr5: 122675214)

South Asians only

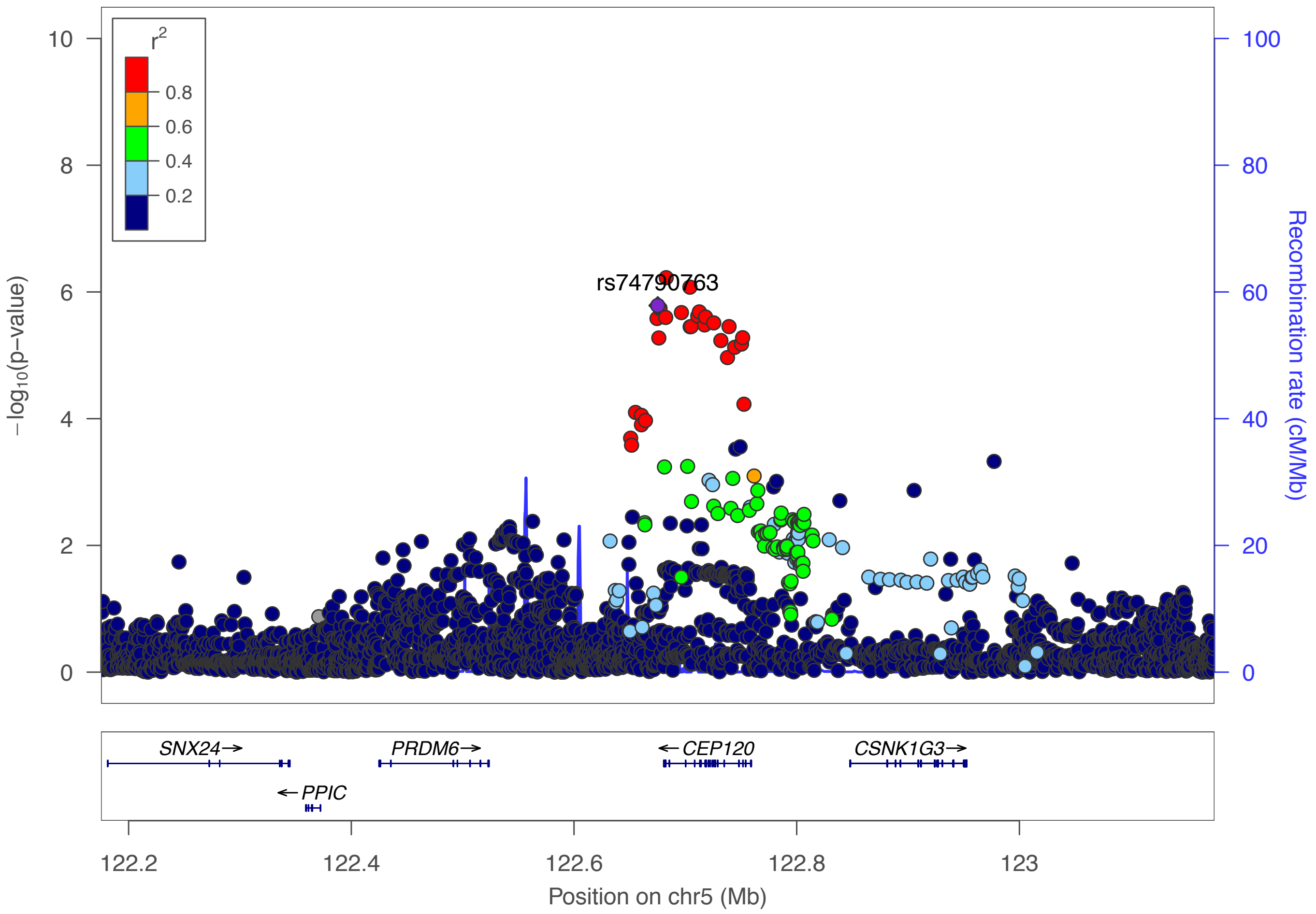

South Asians + Europeans

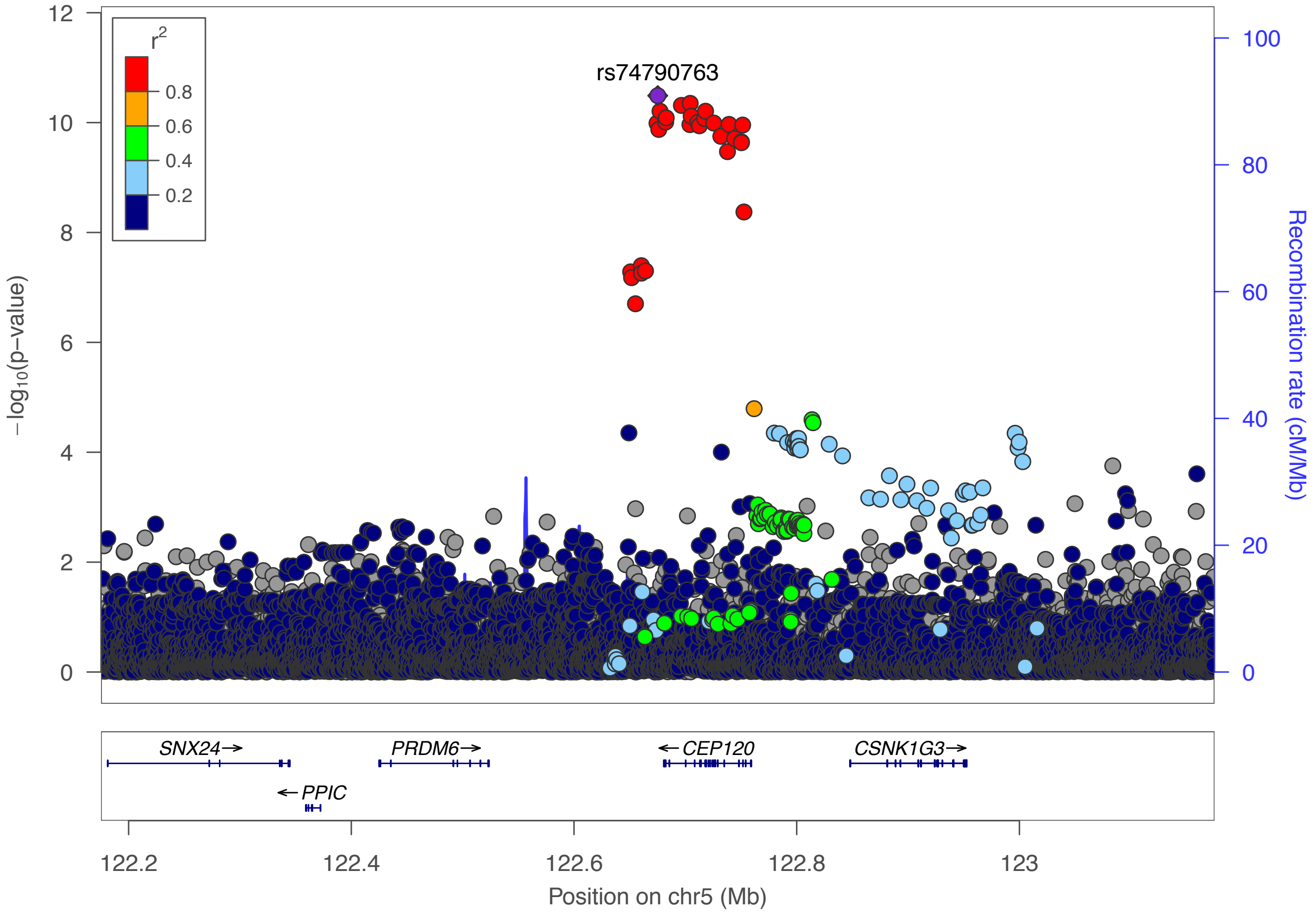

Supplementary Figure 9. Regional plots for rs74790763 showing association of SNPs with T2D for BMI unadjusted model in South Asians only, and in South Asians and Europeans.

rs7765207 (chr6: 138858054)

South Asians only

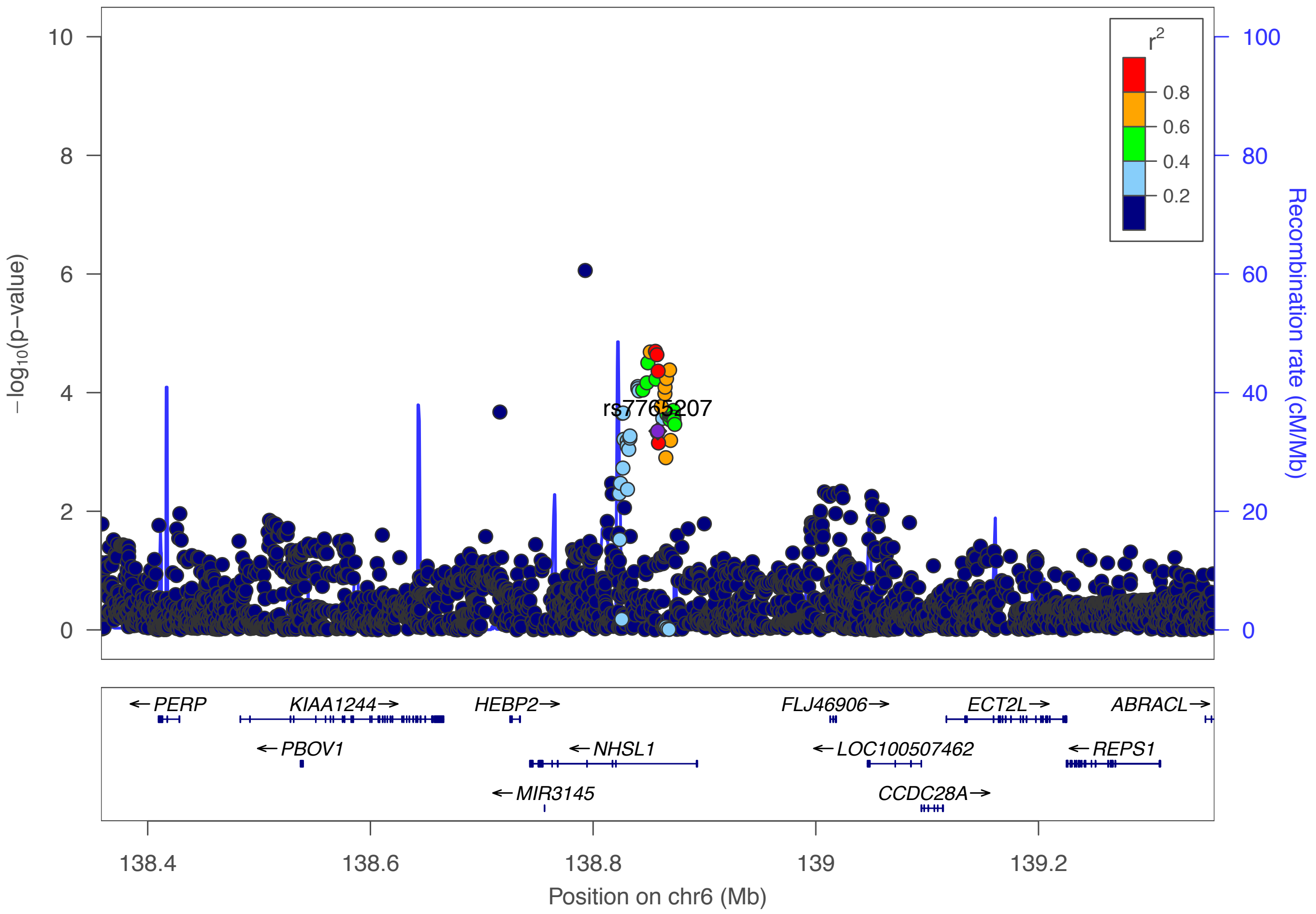

South Asians + Europeans

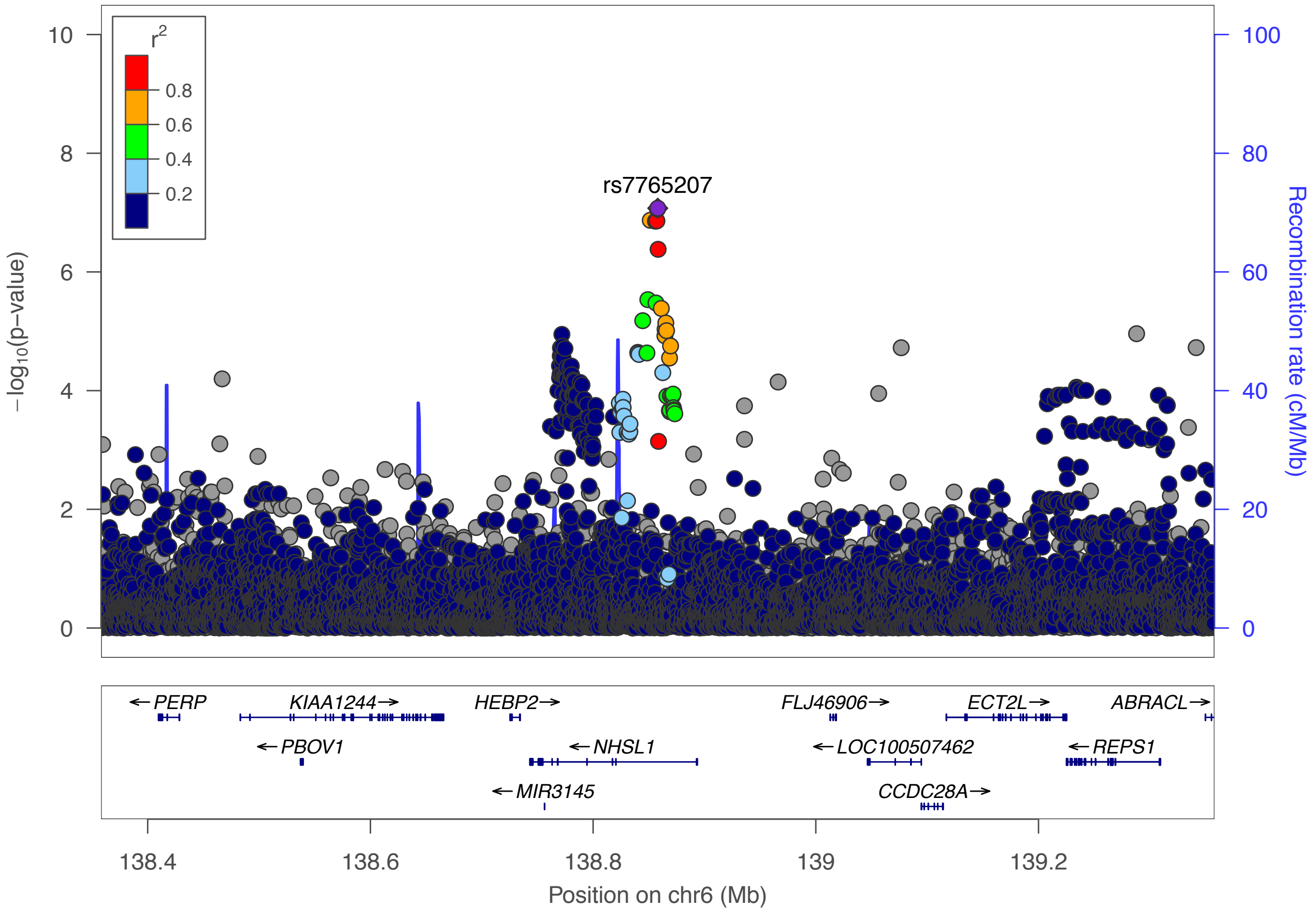

Supplementary Figure 10. Regional plots for rs7765207 showing association of SNPs with T2D for BMI unadjusted model in South Asians only, and in South Asians and Europeans.

rs73689877 (chr7: 40905274)

South Asians only

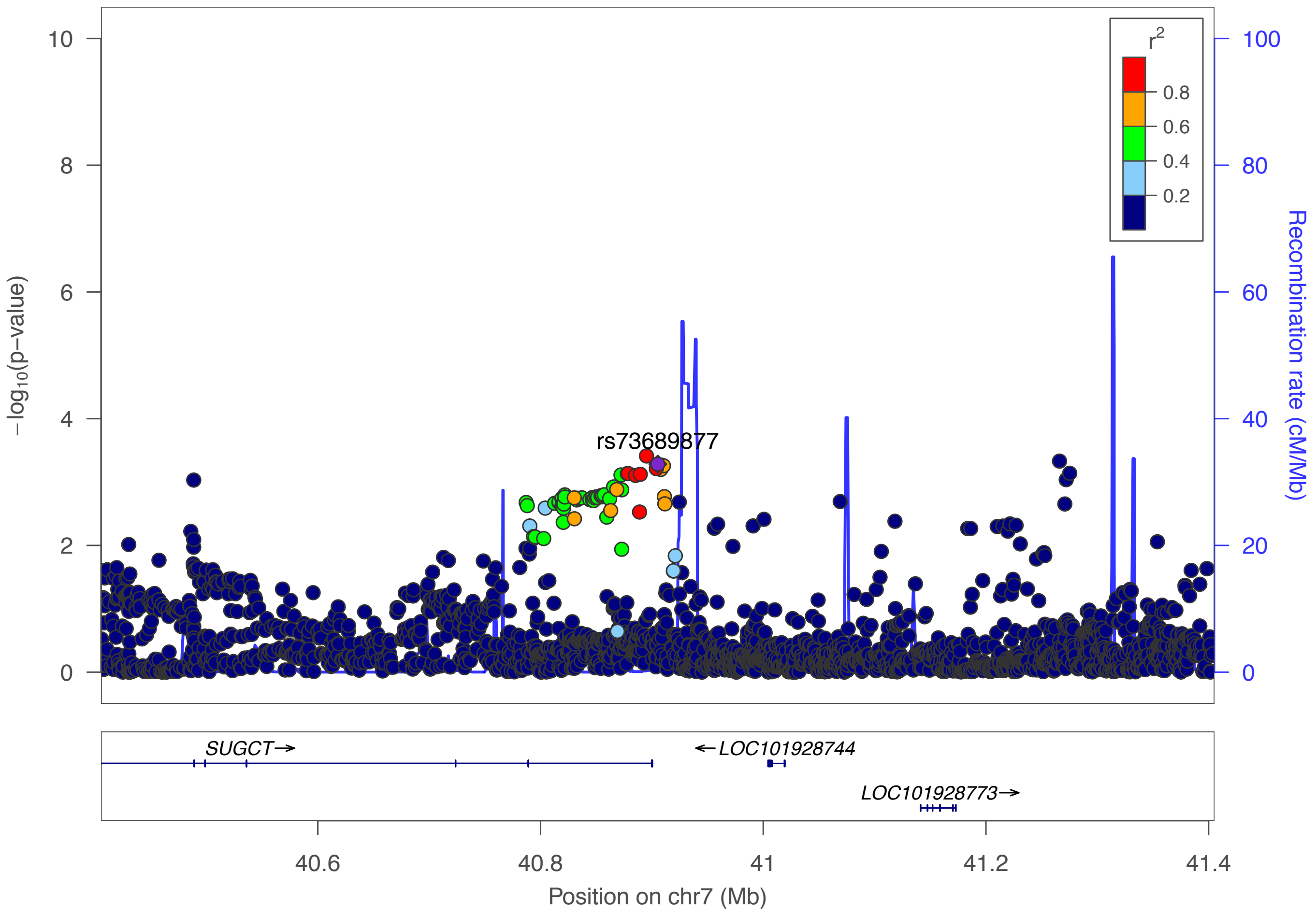

South Asians + Europeans

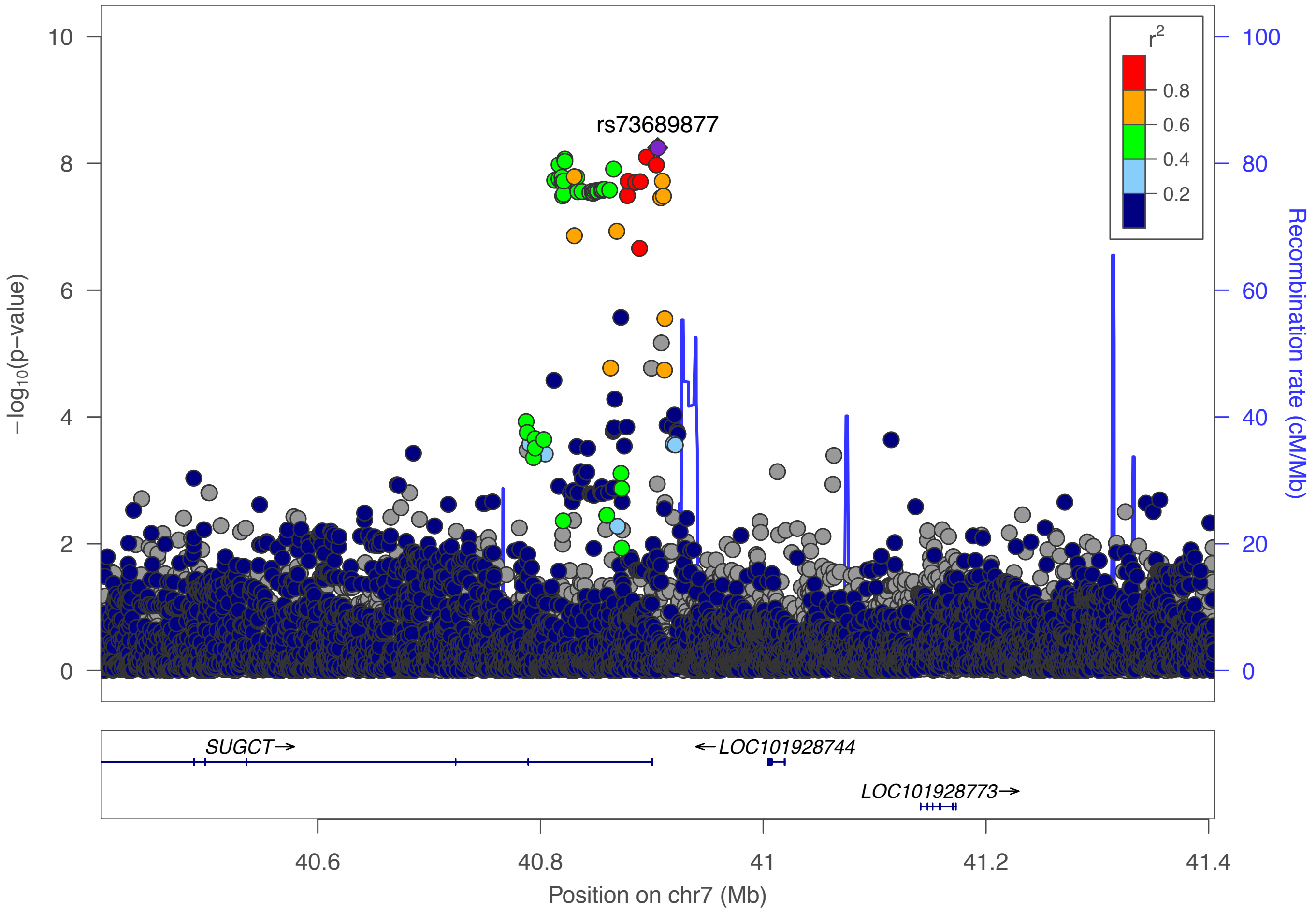

Supplementary Figure 11. Regional plots for rs73689877 showing association of SNPs with T2D for BMI unadjusted model in South Asians only, and in South Asians and Europeans.

rs2980766 (chr8: 8318095)

South Asians only

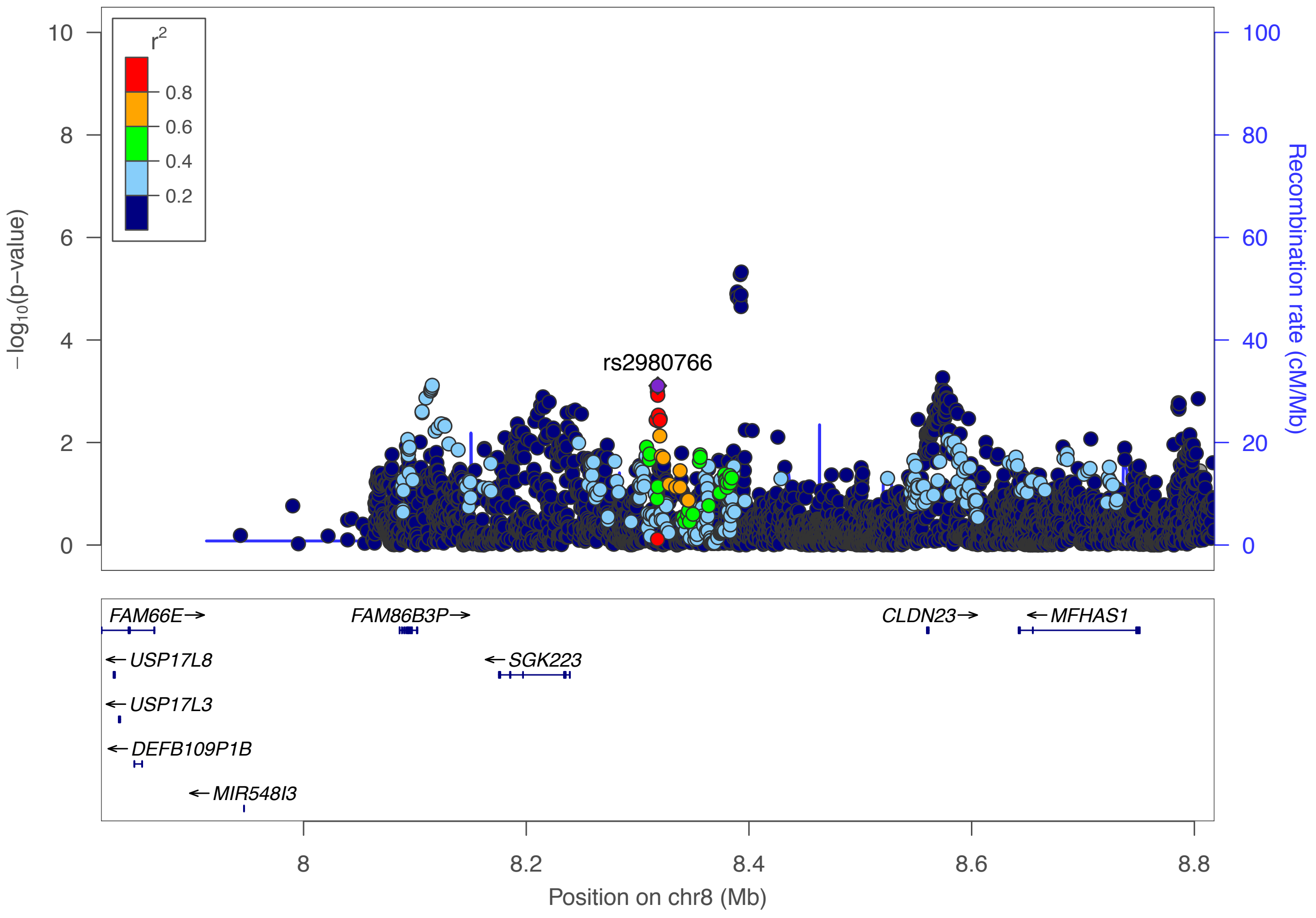

South Asians + Europeans

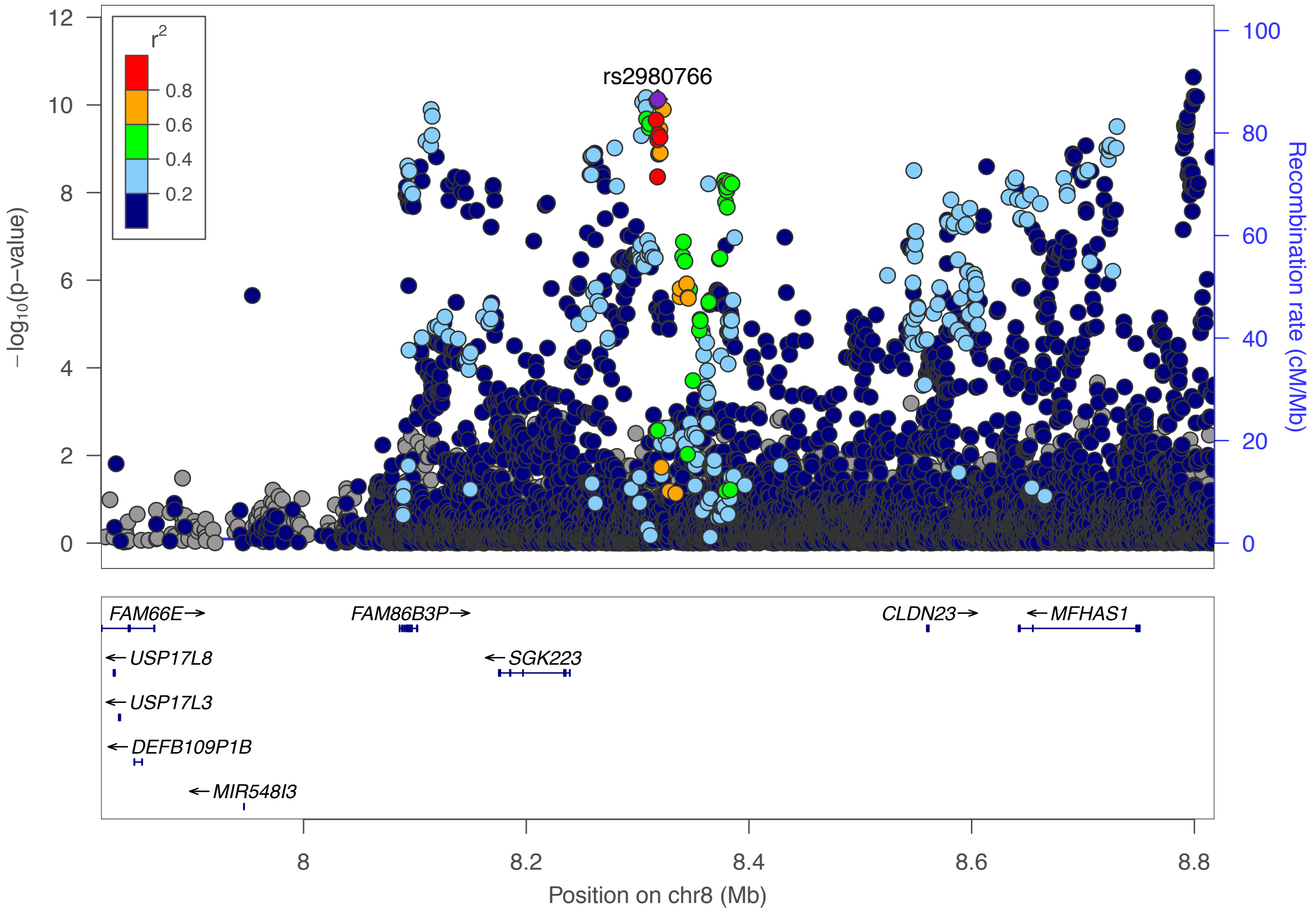

Supplementary Figure 12. Regional plots for rs2980766 showing association of SNPs with T2D for BMI unadjusted model in South Asians only, and in South Asians and Europeans.

rs62486442 (chr8: 12623463)

South Asians only

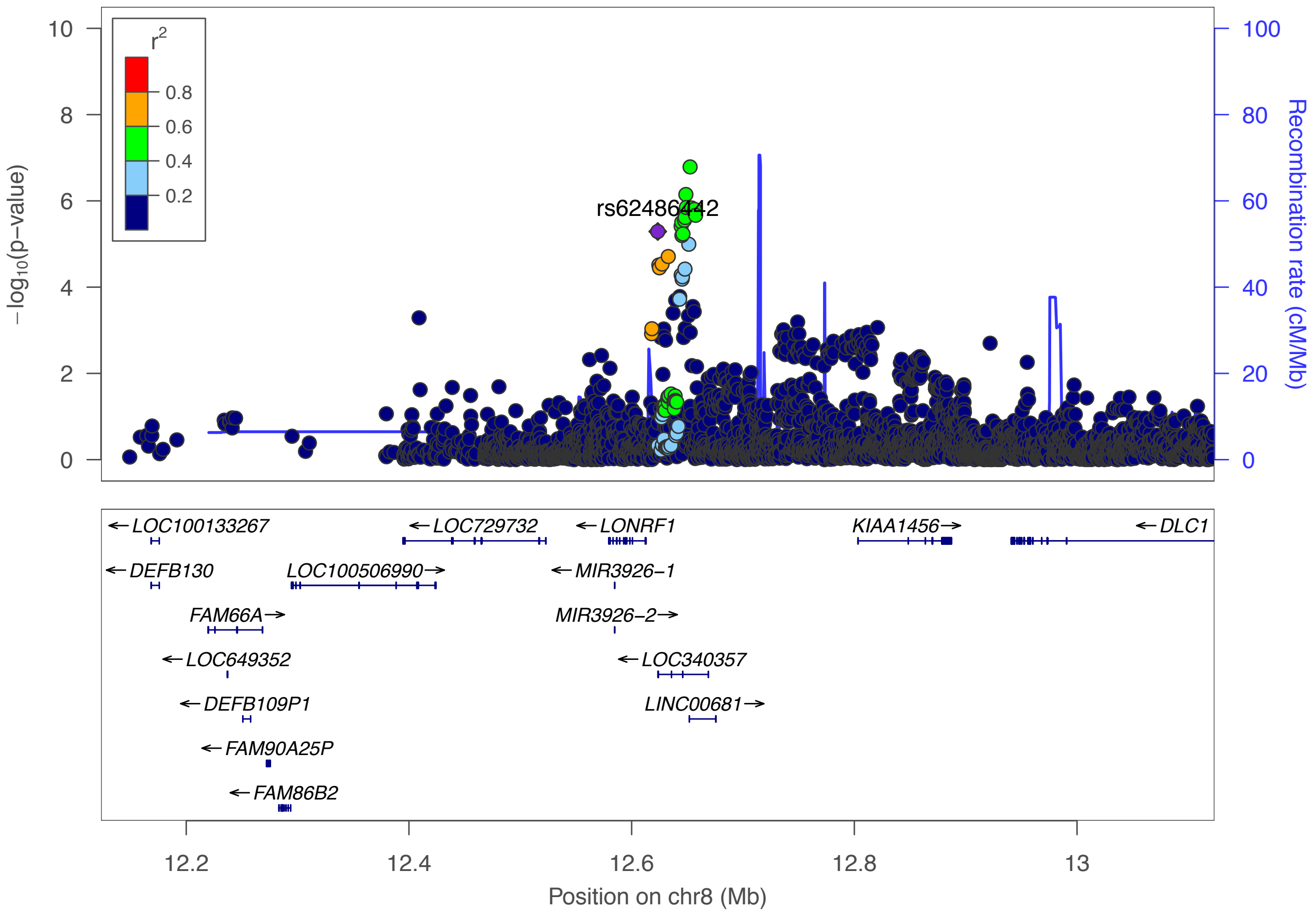

South Asians + Europeans

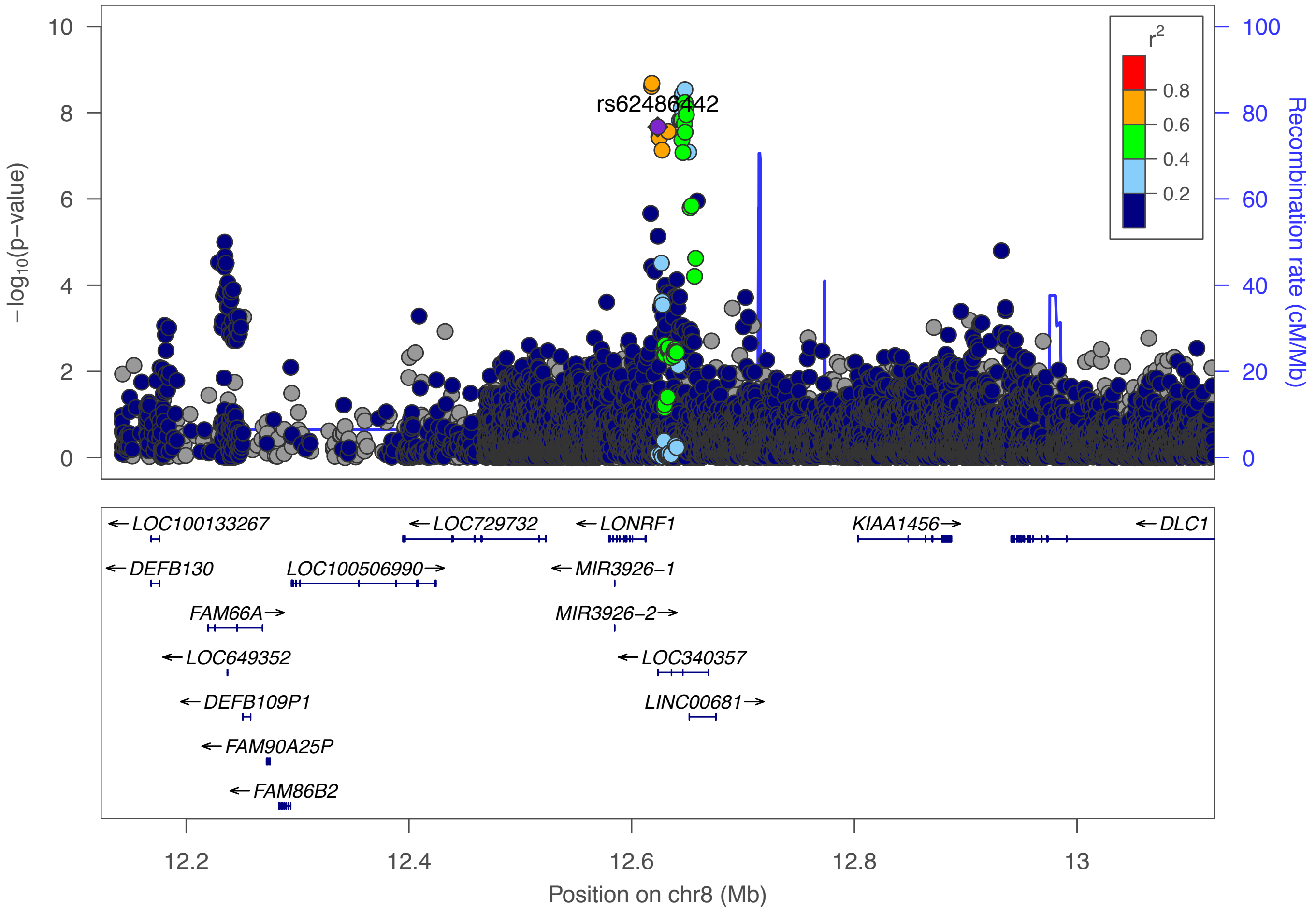

Supplementary Figure 13. Regional plots for rs62486442 showing association of SNPs with T2D for BMI unadjusted model in South Asians only, and in South Asians and Europeans.

rs13257283 (chr8: 105608497)

South Asians only

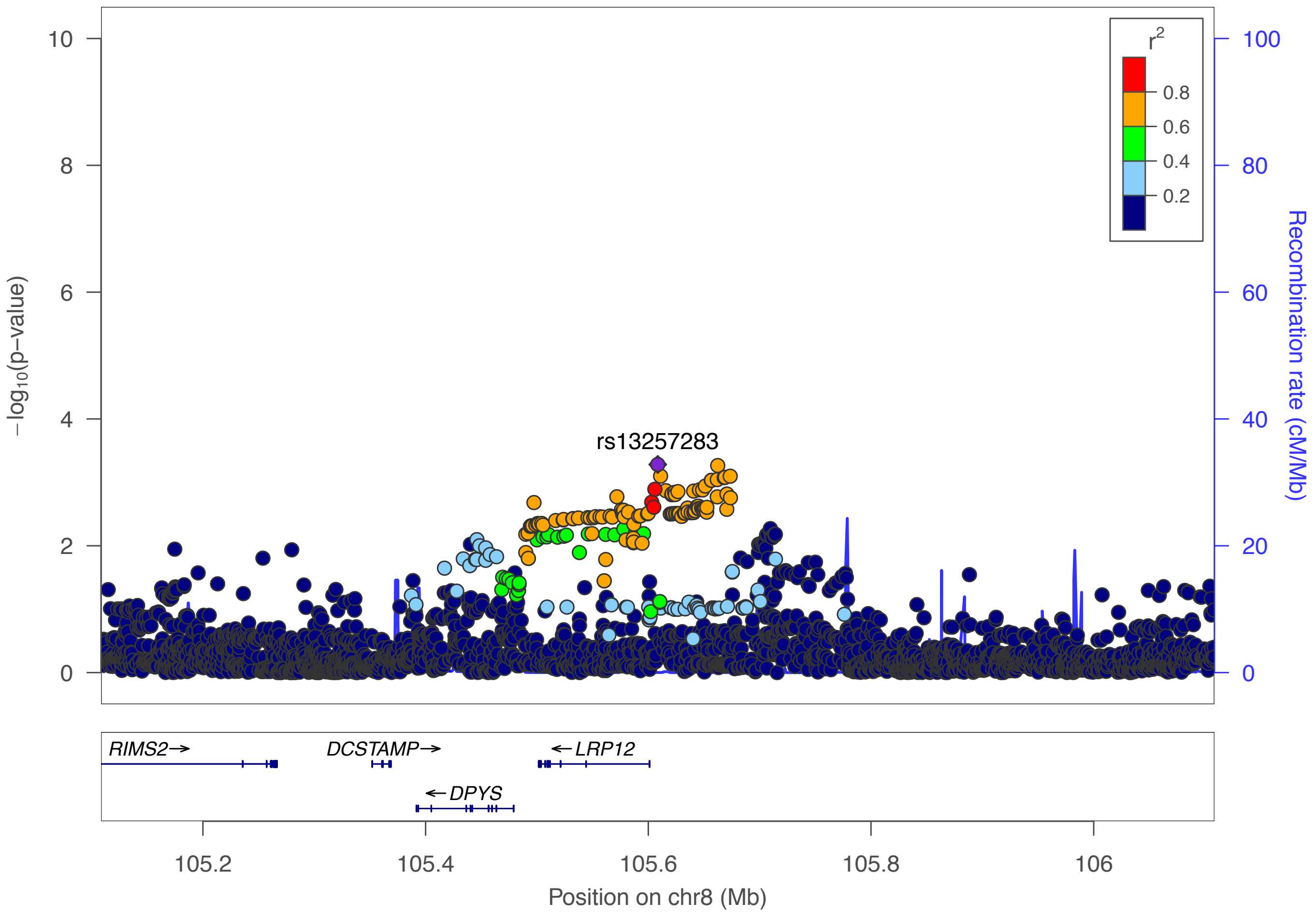

South Asians + Europeans

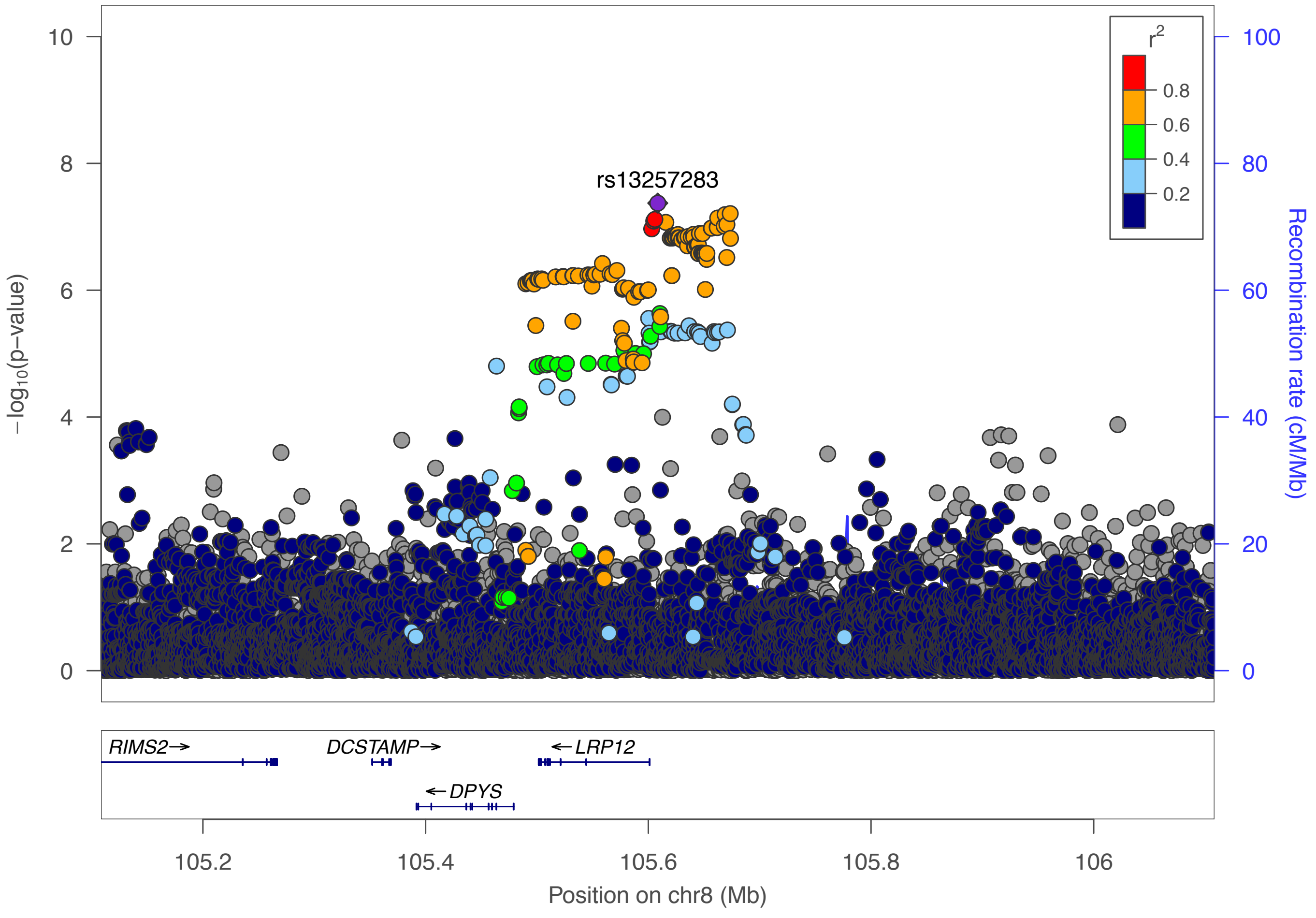

Supplementary Figure 14. Regional plots for rs13257283 showing association of SNPs with T2D for BMI unadjusted model in South Asians only, and in South Asians and Europeans

rs2488597 (chr9: 125992073)

South Asians only

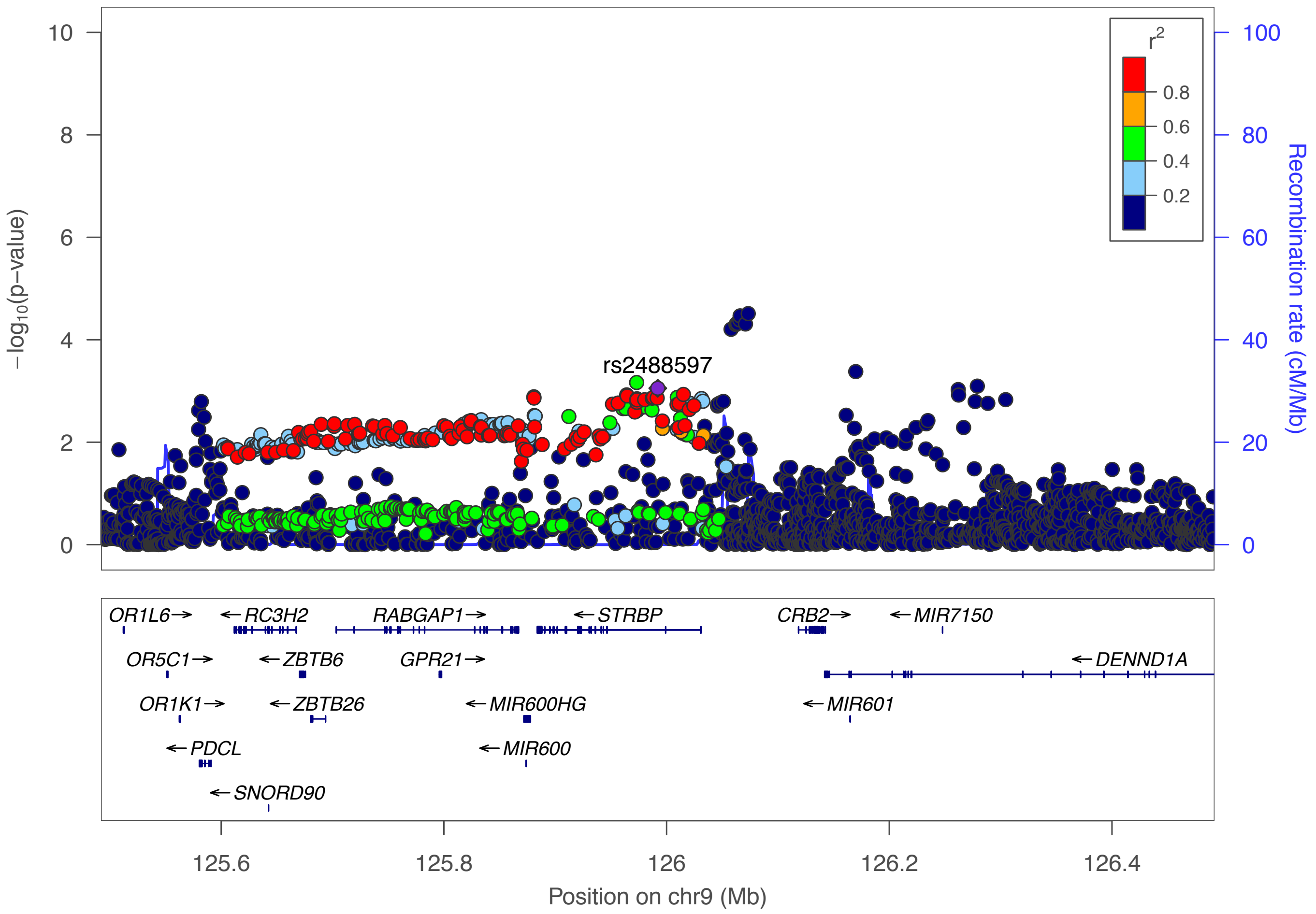

South Asians + Europeans

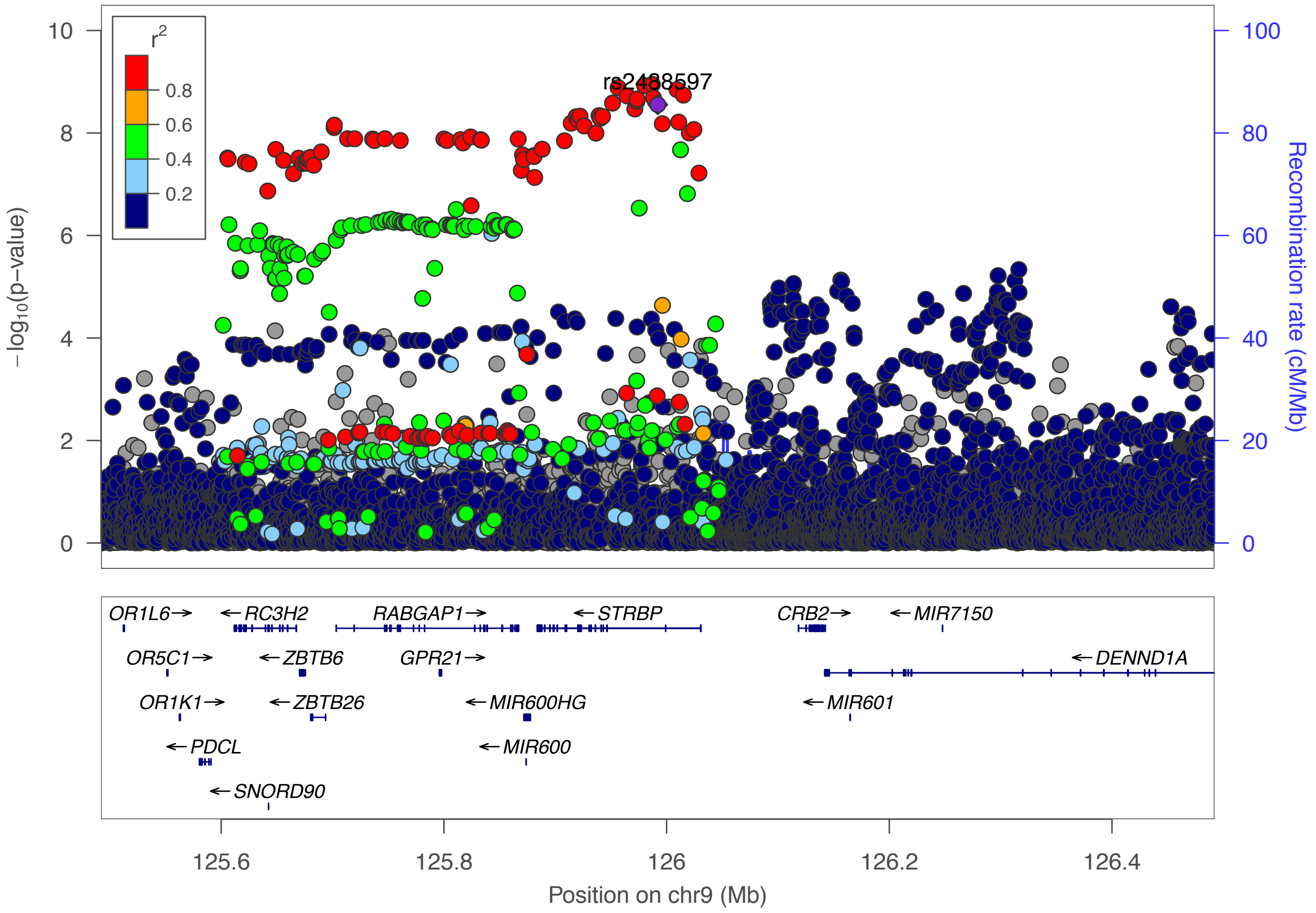

Supplementary Figure 15. Regional plots for rs2488597 showing association of SNPs with T2D for BMI unadjusted model in South Asians only, and in South Asians and Europeans.

rs2114824 (chr10: 88119015)

South Asians only

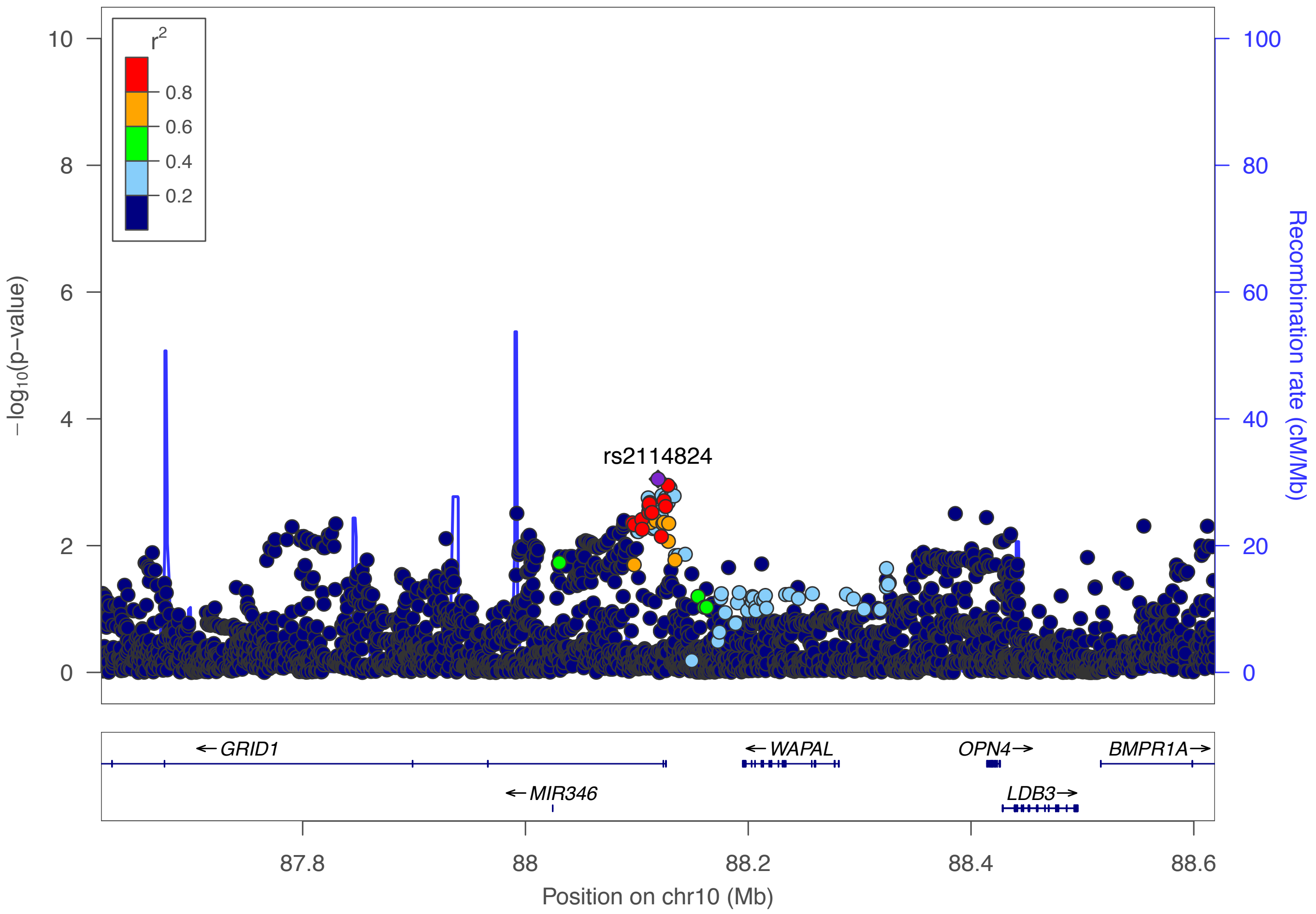

South Asians + Europeans

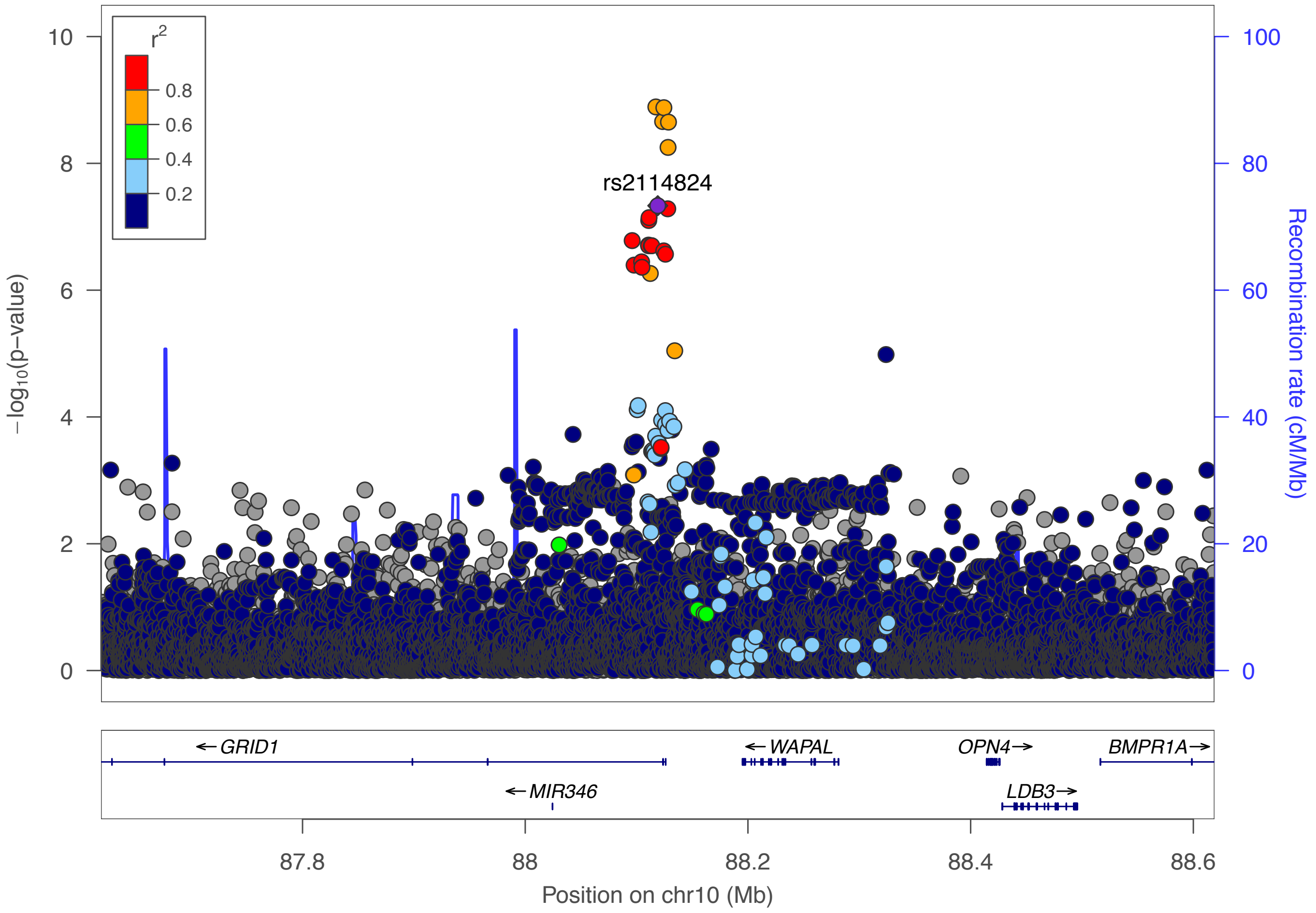

Supplementary Figure 16. Regional plots for rs2114824 showing association of SNPs with T2D for BMI unadjusted model in South Asians only, and in South Asians and Europeans.

rs10748694 (chr10:99056190)

South Asians only

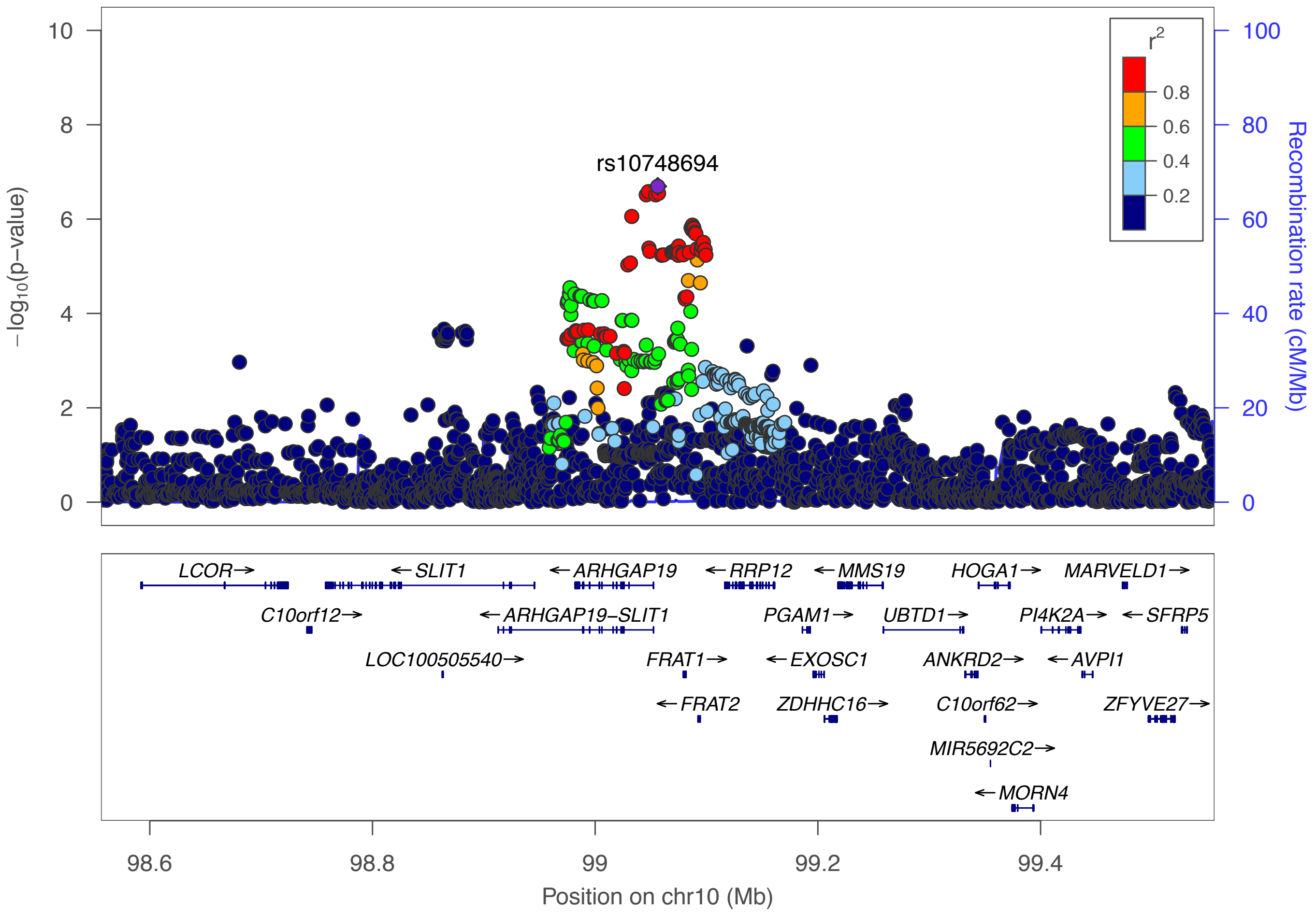

South Asians + Europeans

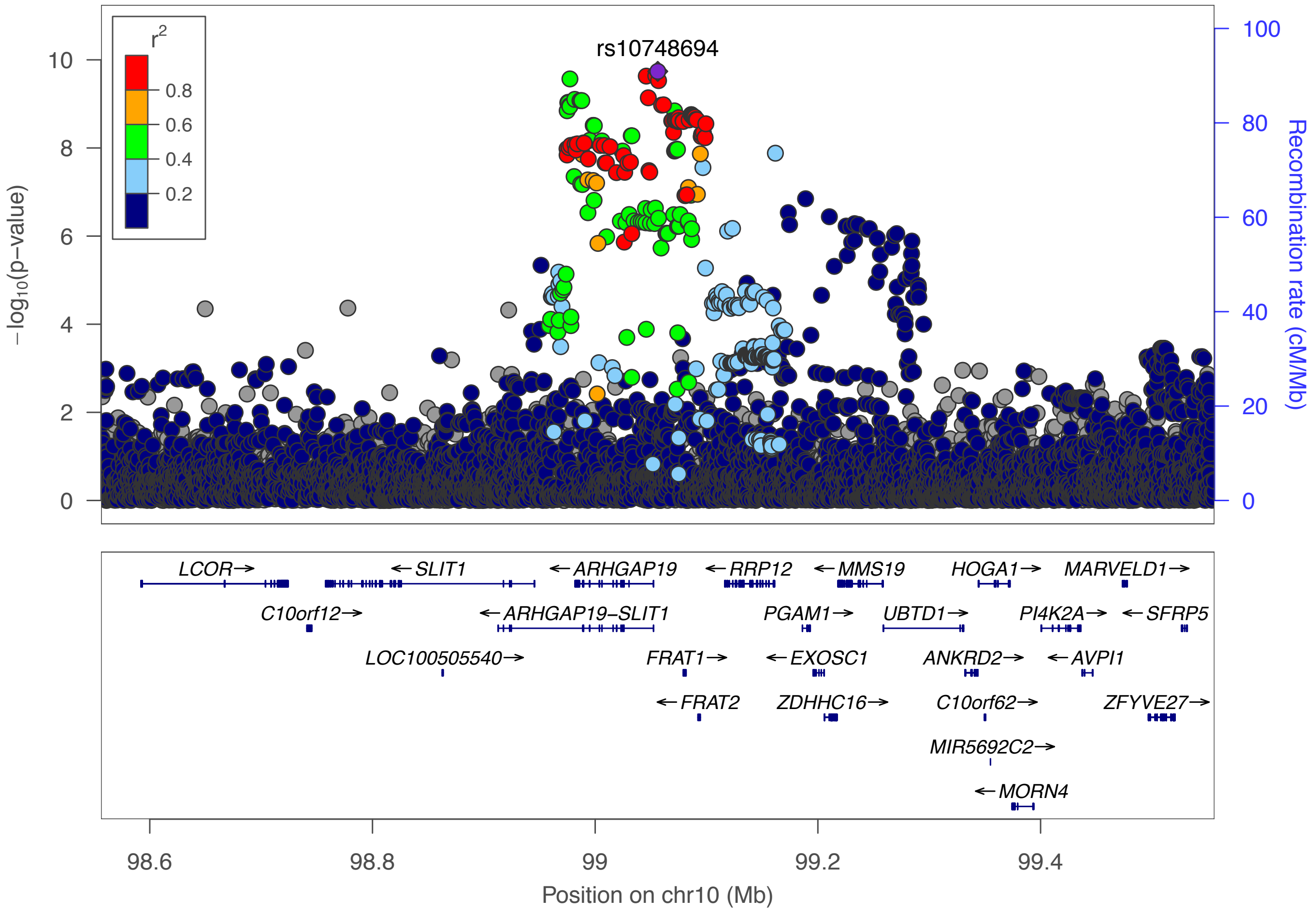

Supplementary Figure 17. Regional plots for rs10748694 showing association of SNPs with T2D for BMI unadjusted model in South Asians only, and in South Asians and Europeans.

rs7123361 (chr11:76161389)

South Asians only

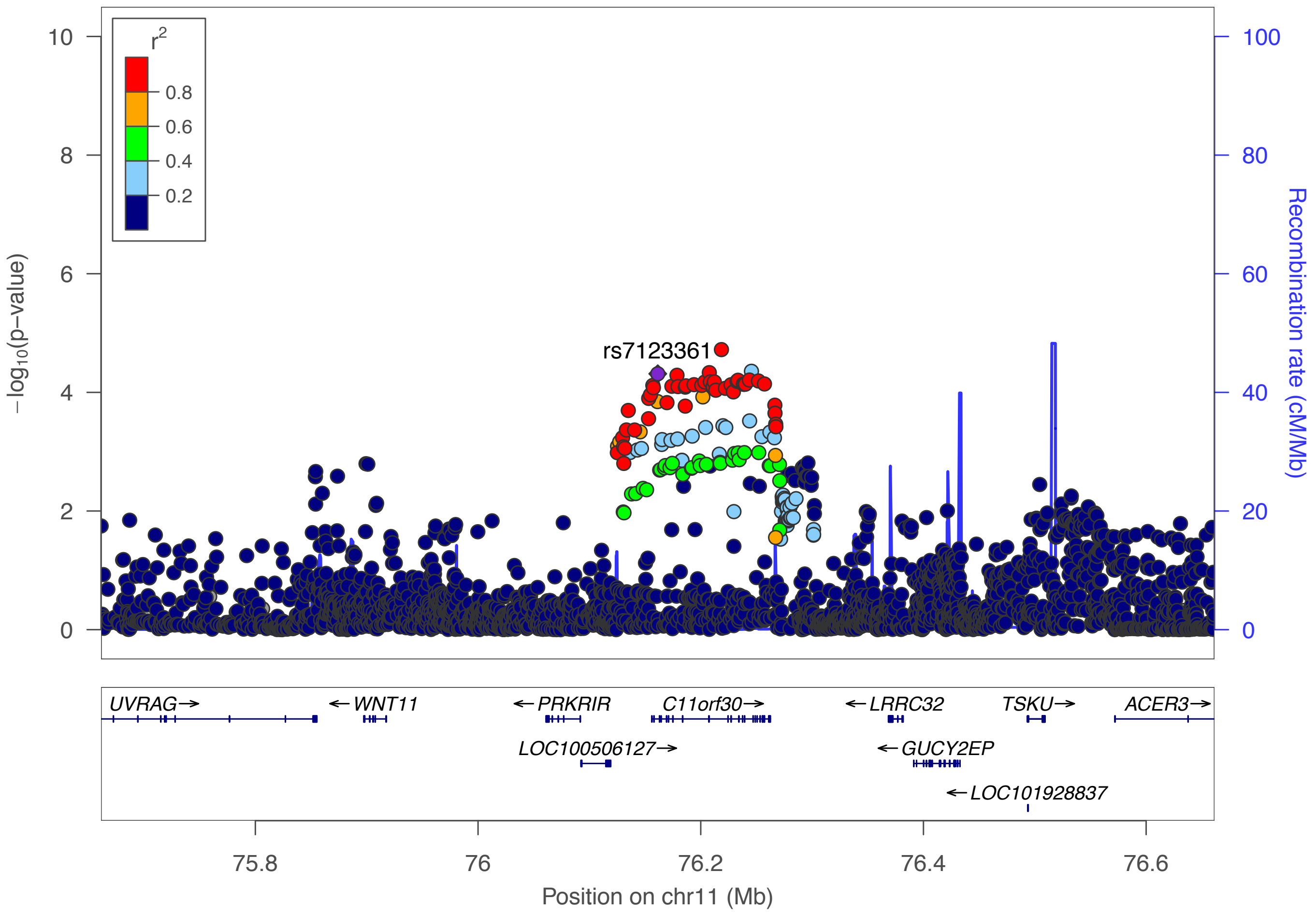

South Asians + Europeans

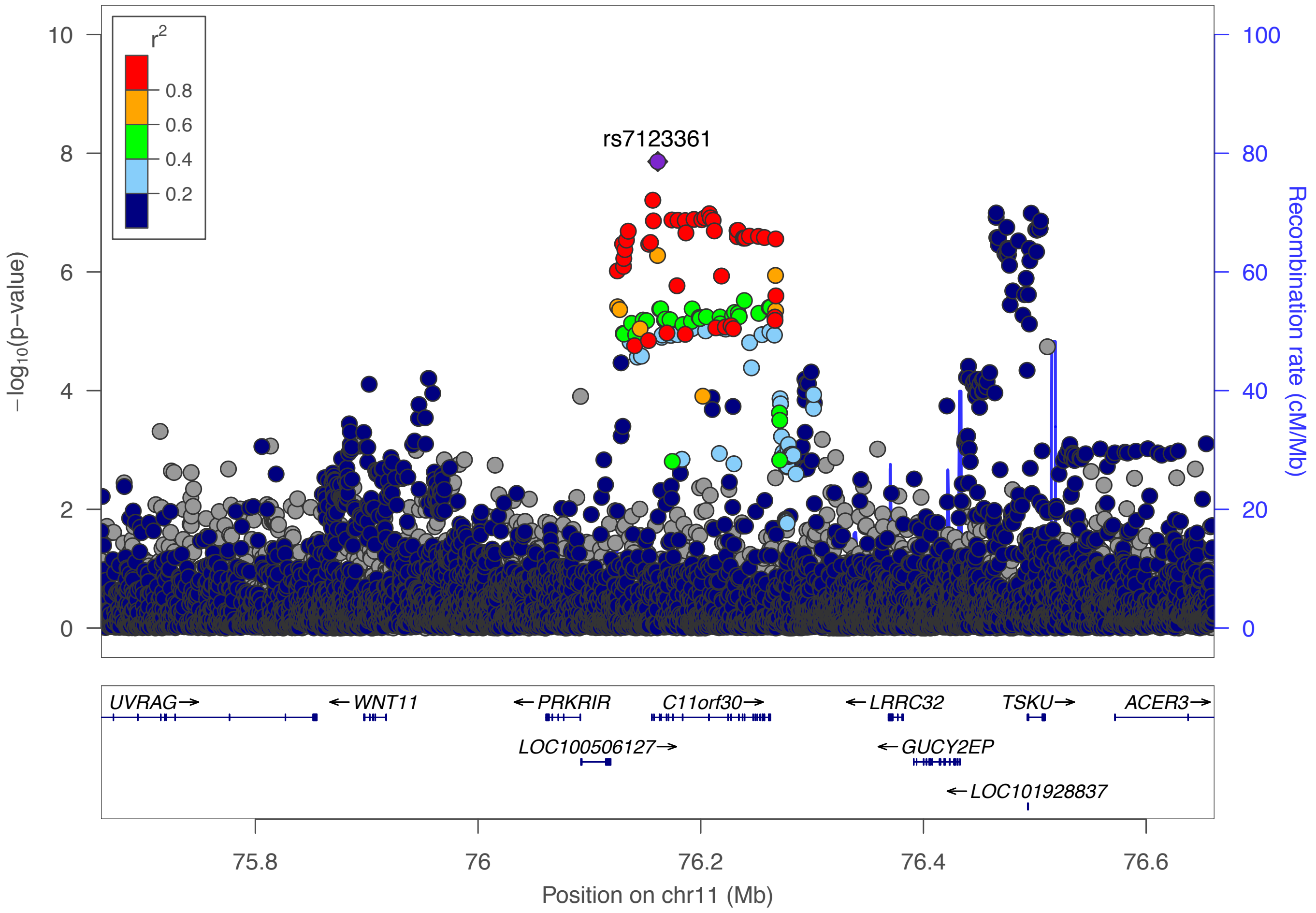

Supplementary Figure 18. Regional plots for rs7123361 showing association of SNPs with T2D for BMI unadjusted model in South Asians only, and in South Asians and Europeans.

rs9568861 (chr13:54079446)

South Asians only

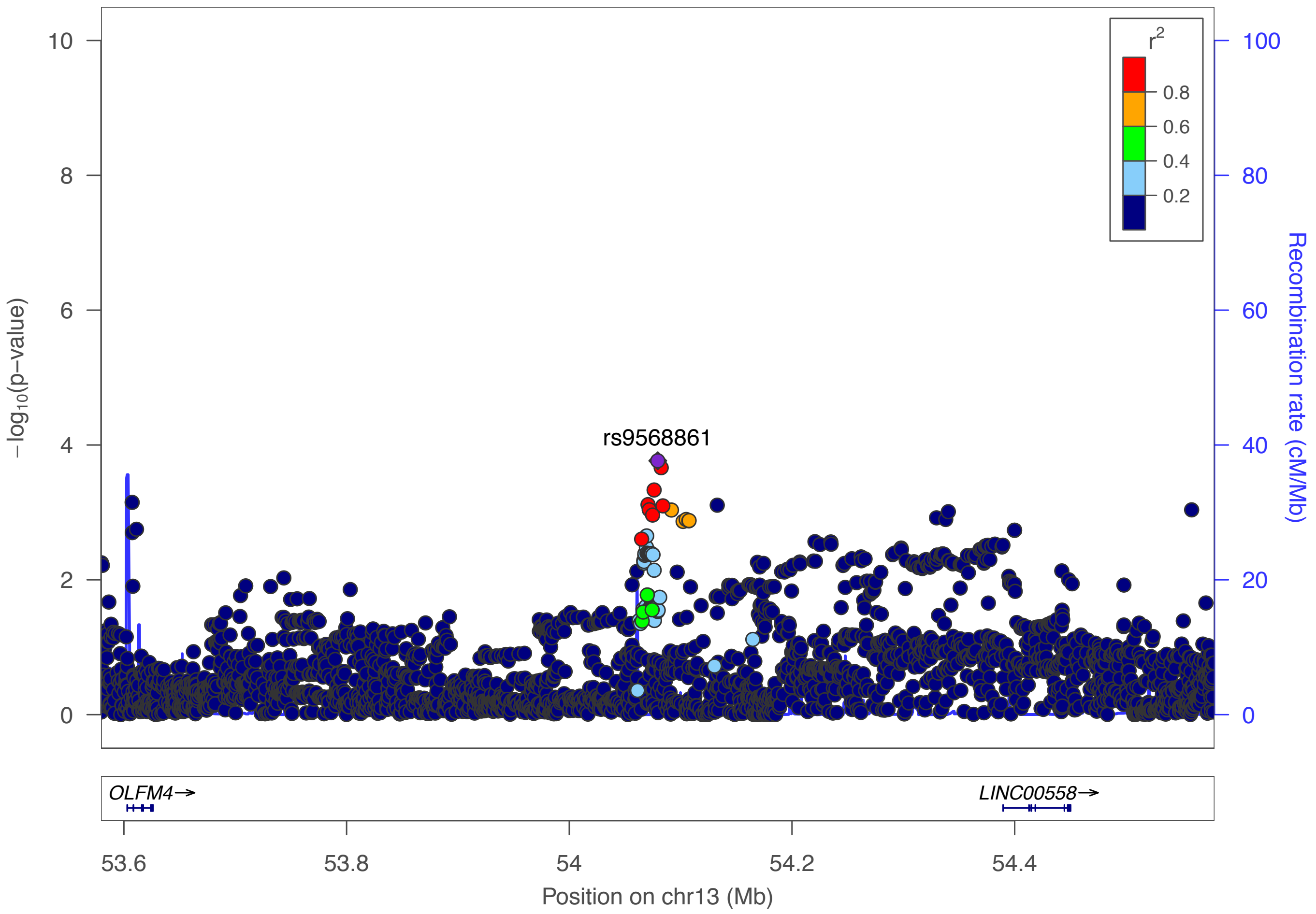

South Asians + Europeans

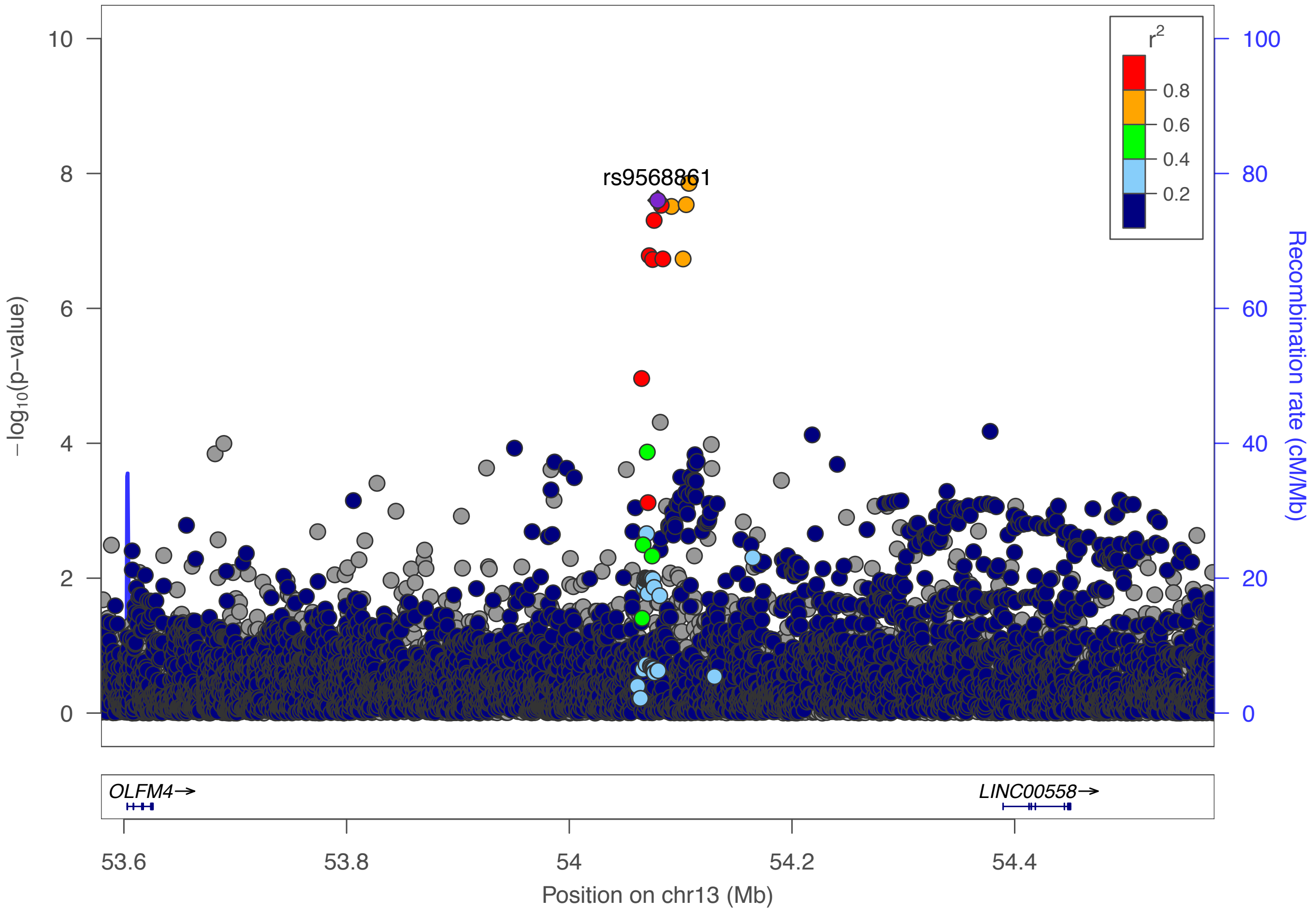

Supplementary Figure 19. Regional plots for rs9568861 showing association of SNPs with T2D for BMI unadjusted model in South Asians only, and in South Asians and Europeans.

rs76141923 (chr13:81646630)

South Asians only

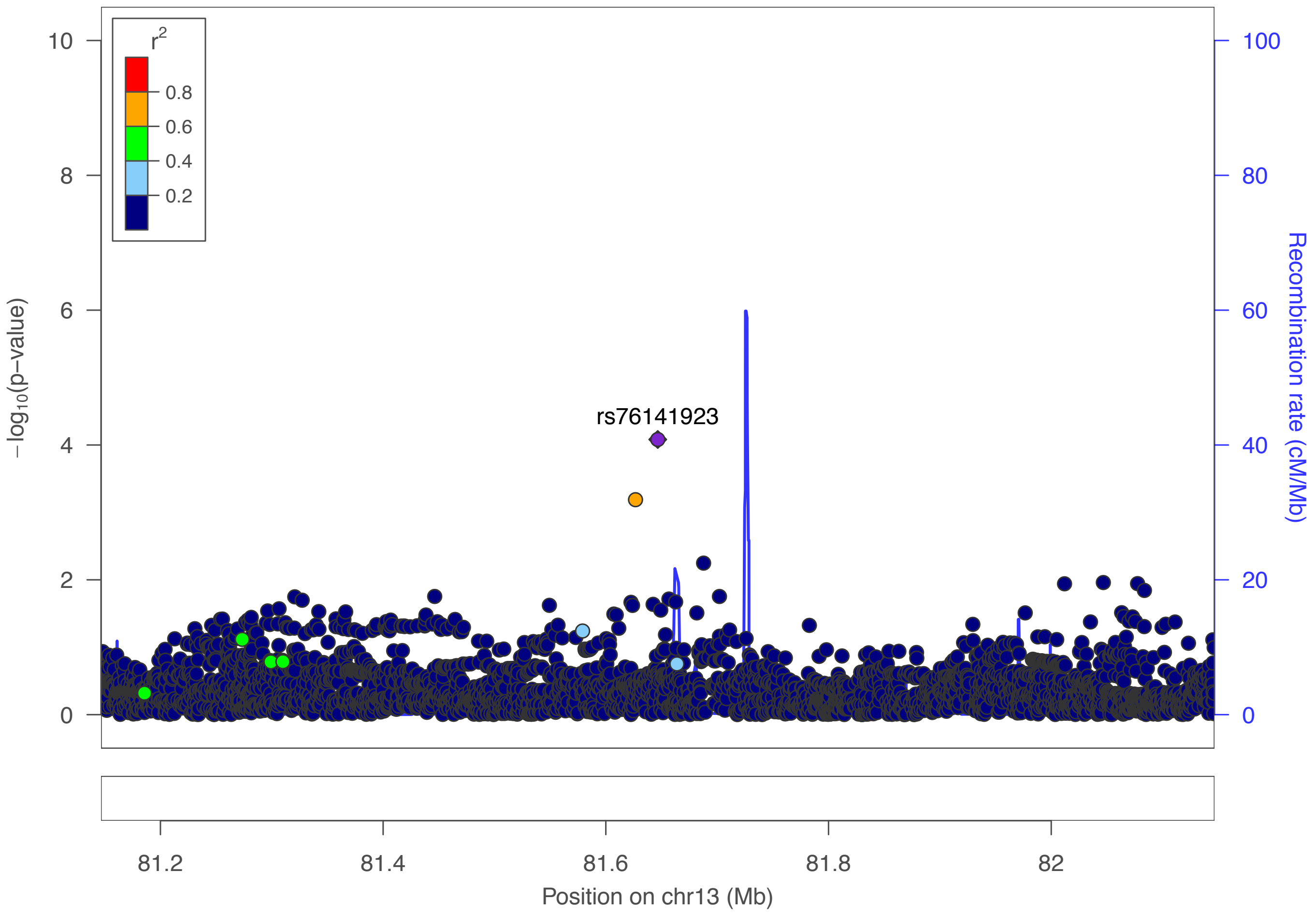

South Asians + Europeans

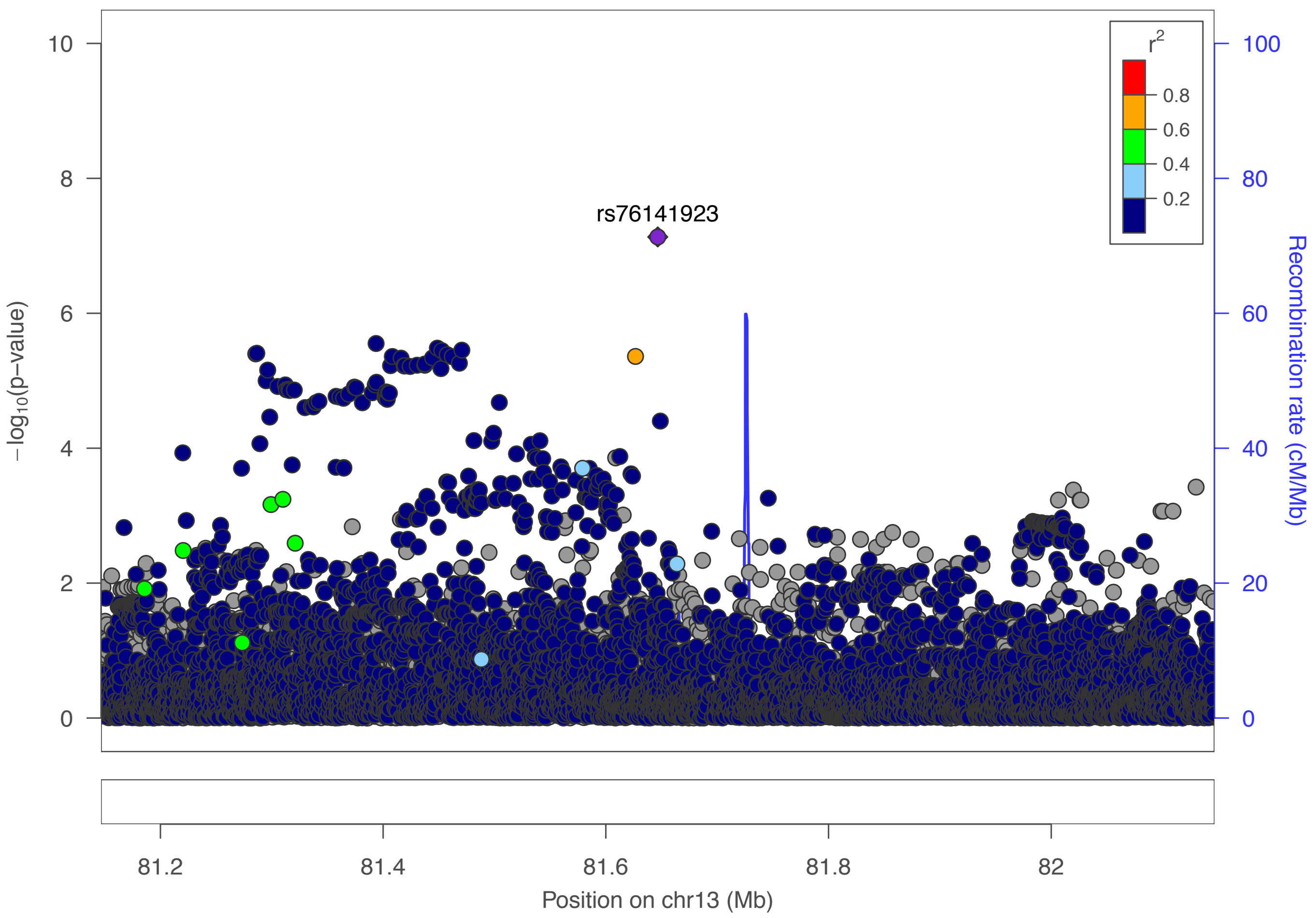

Supplementary Figure 20. Regional plots for rs76141923 showing association of SNPs with T2D for BMI unadjusted model in South Asians only, and in South Asians and Europeans.

rs28790585 (chr15:39668870)

South Asians only

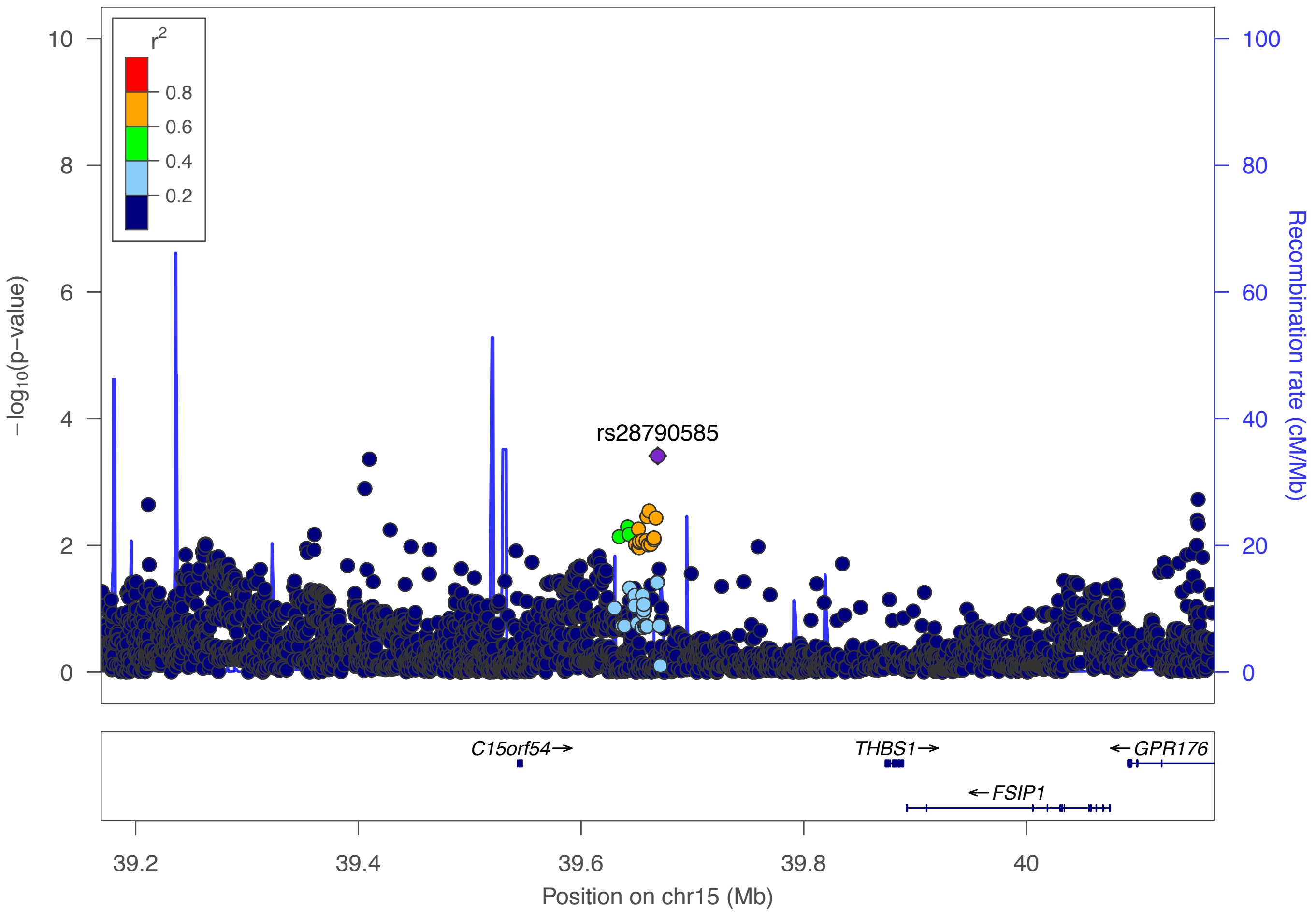

South Asians + Europeans

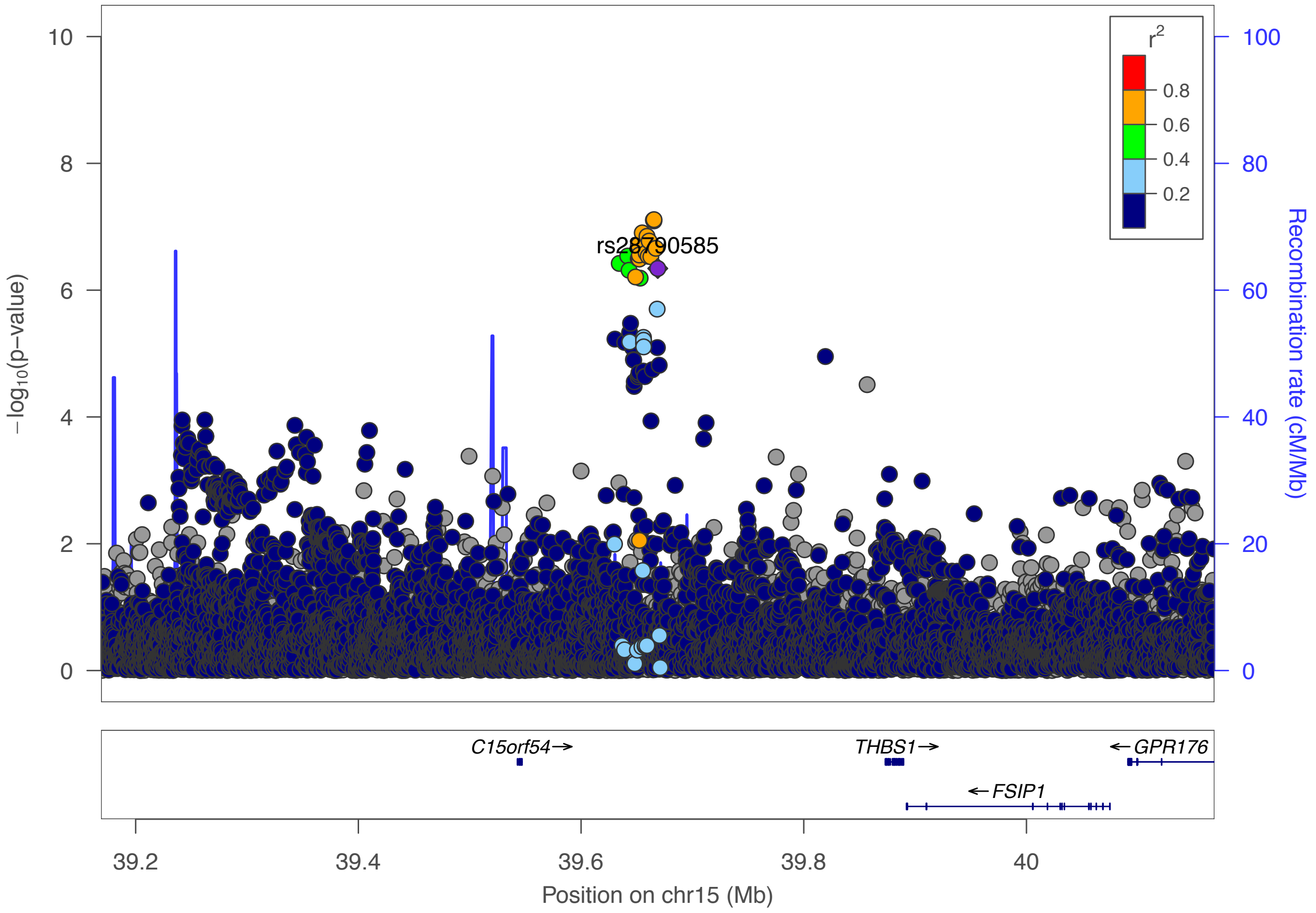

Supplementary Figure 21. Regional plots for rs28790585 showing association of SNPs with T2D for BMI unadjusted model in South Asians only, and in South Asians and Europeans.

rs7261425 (chr20:20068635)

South Asians only

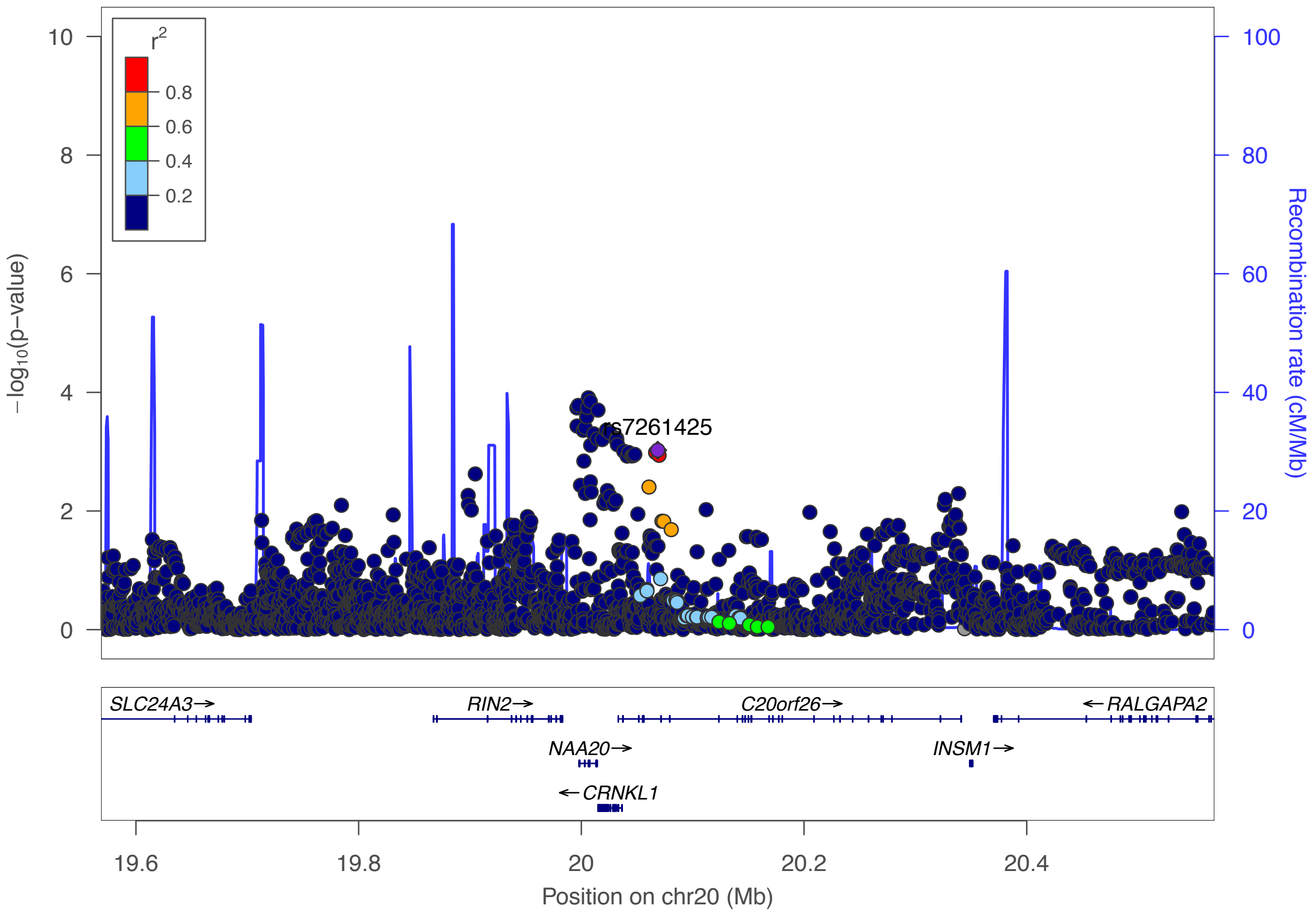

South Asians + Europeans

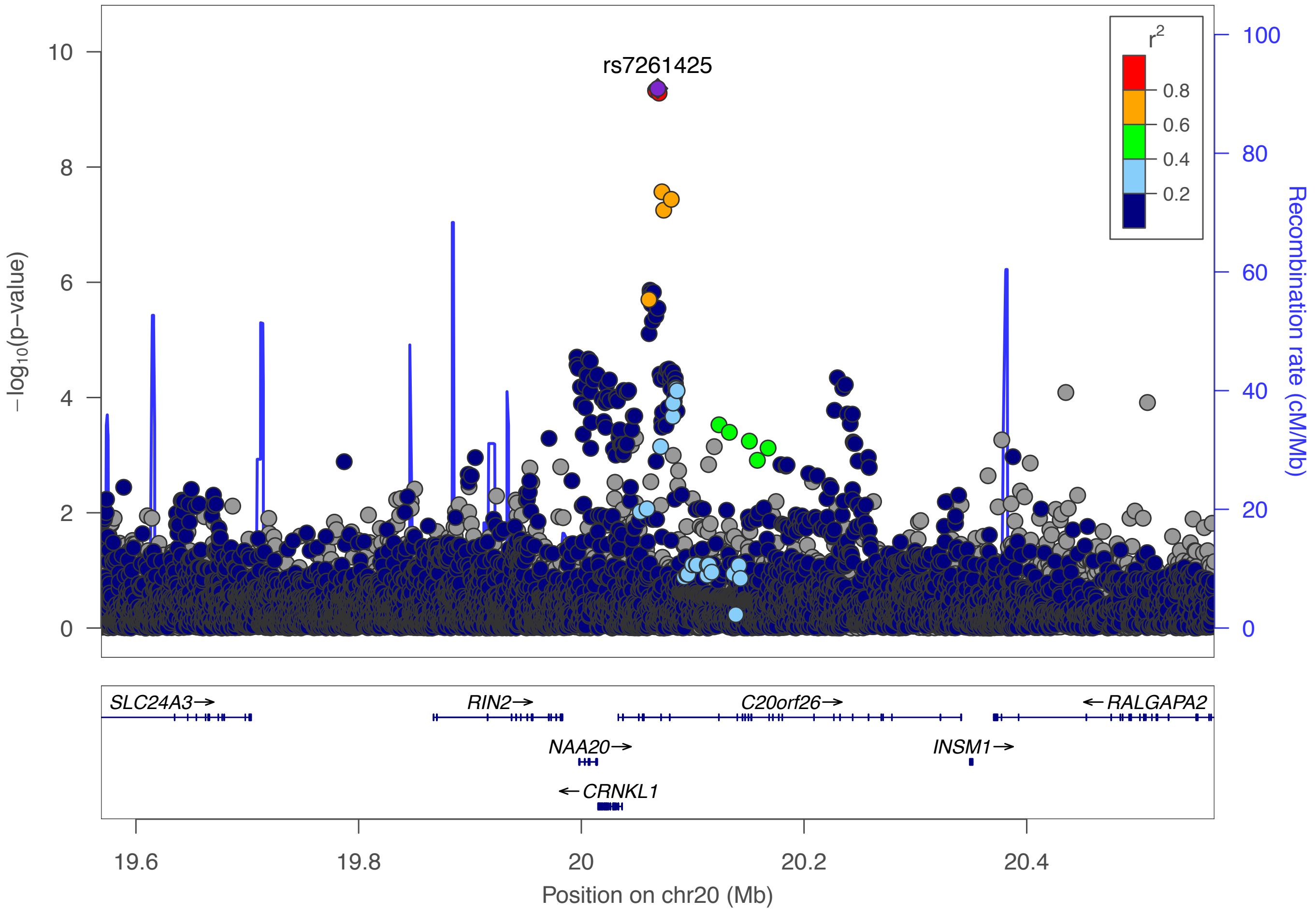

Supplementary Figure 22. Regional plots for rs7261425 showing association of SNPs with T2D for BMI unadjusted model in South Asians only, and in South Asians and Europeans.

rs2065703 (chr20:31966698)

South Asians only

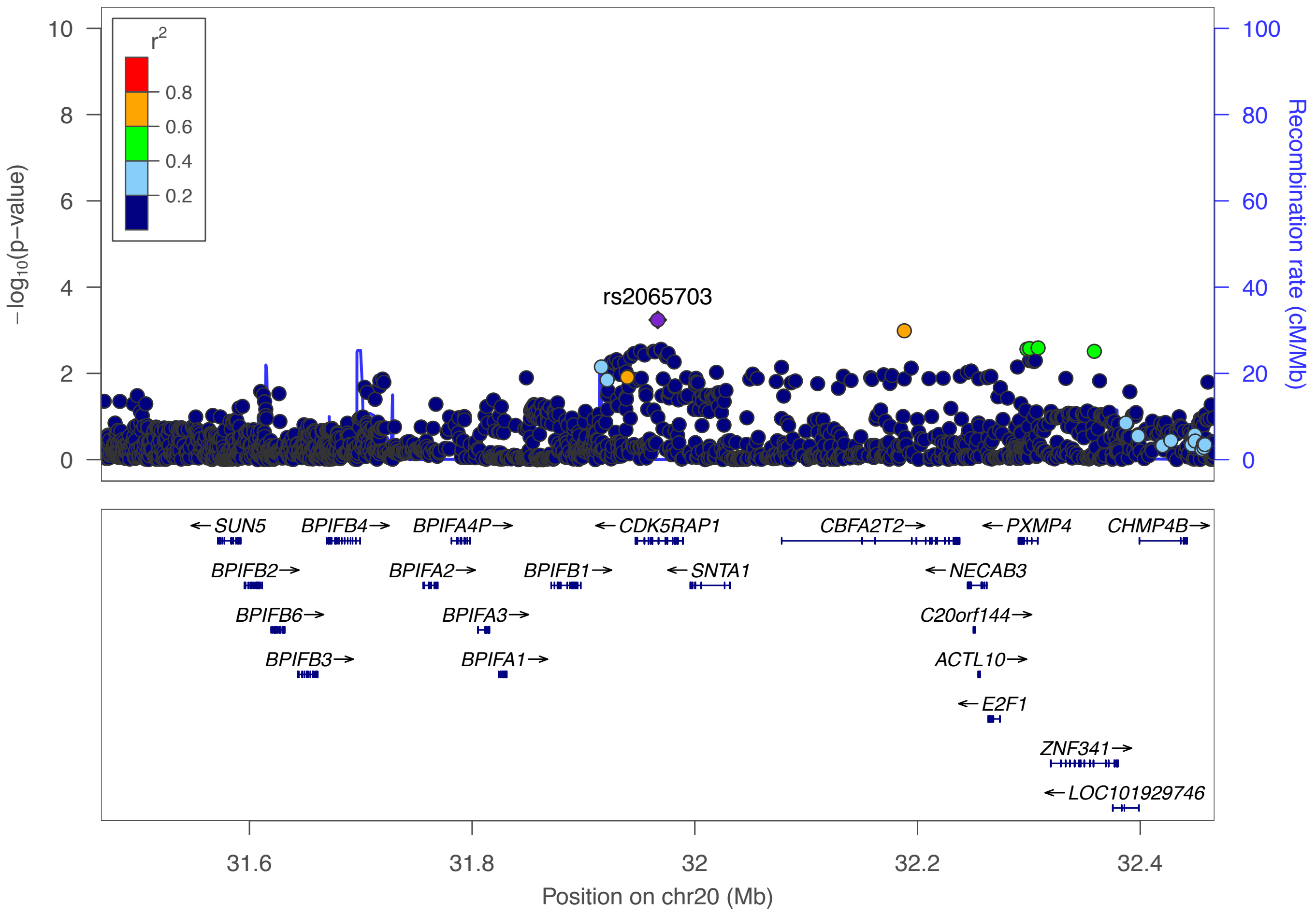

South Asians + Europeans

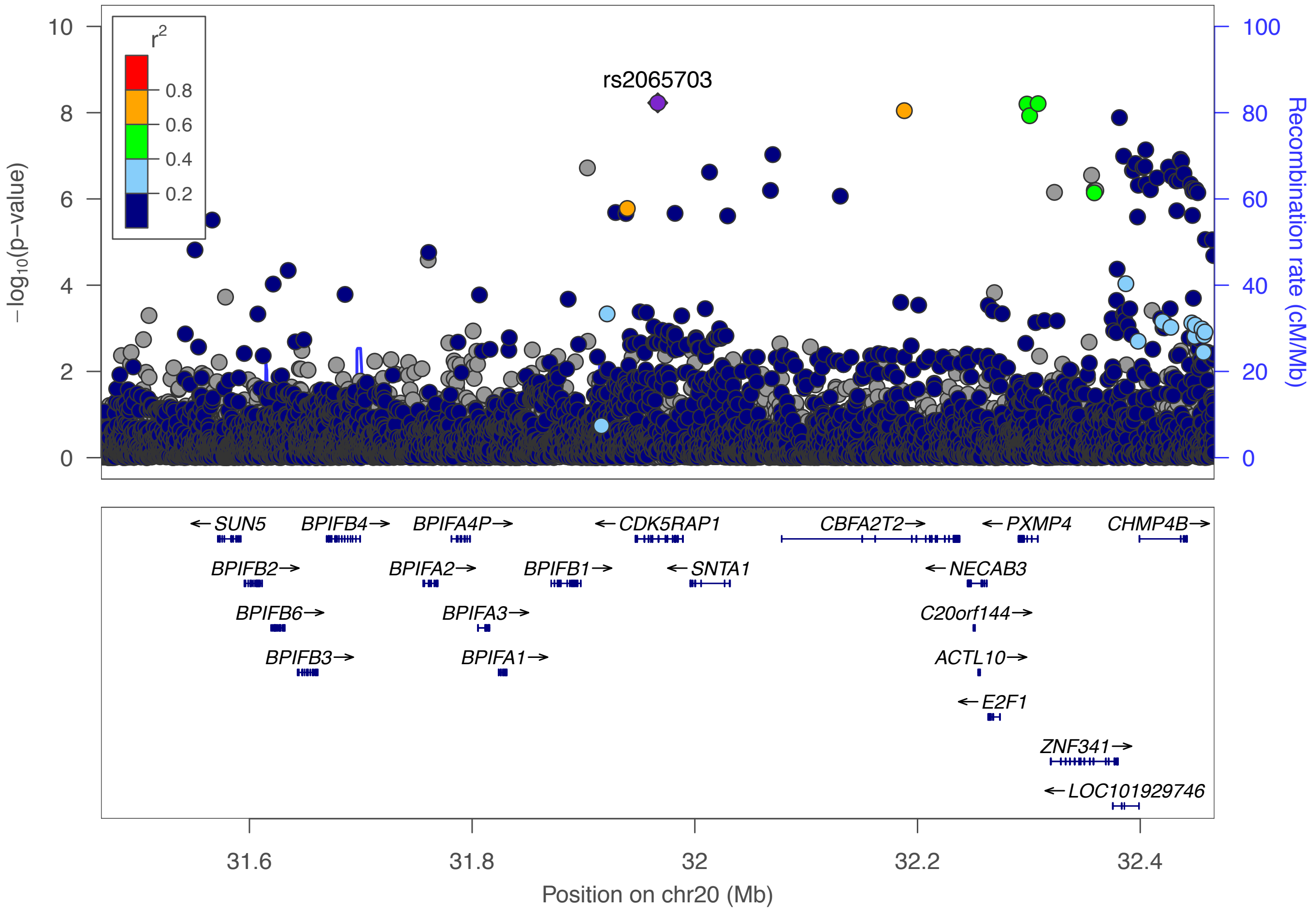

Supplementary Figure 23. Regional plots for rs2065703 showing association of SNPs with T2D for BMI unadjusted model in South Asians only, and in South Asians and Europeans.

rs10916784 (chr1: 20729451)

South Asians only

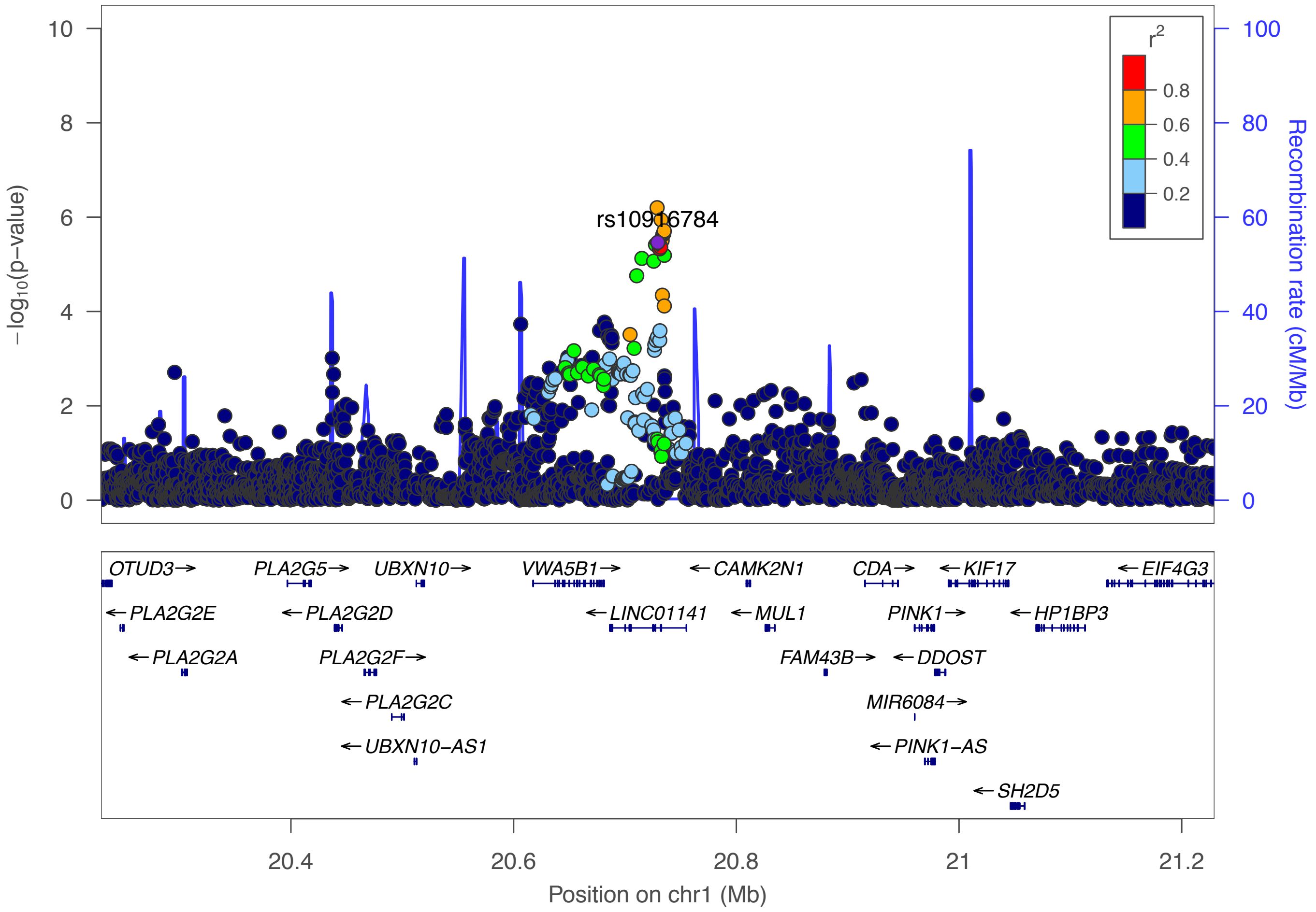

South Asians + Europeans

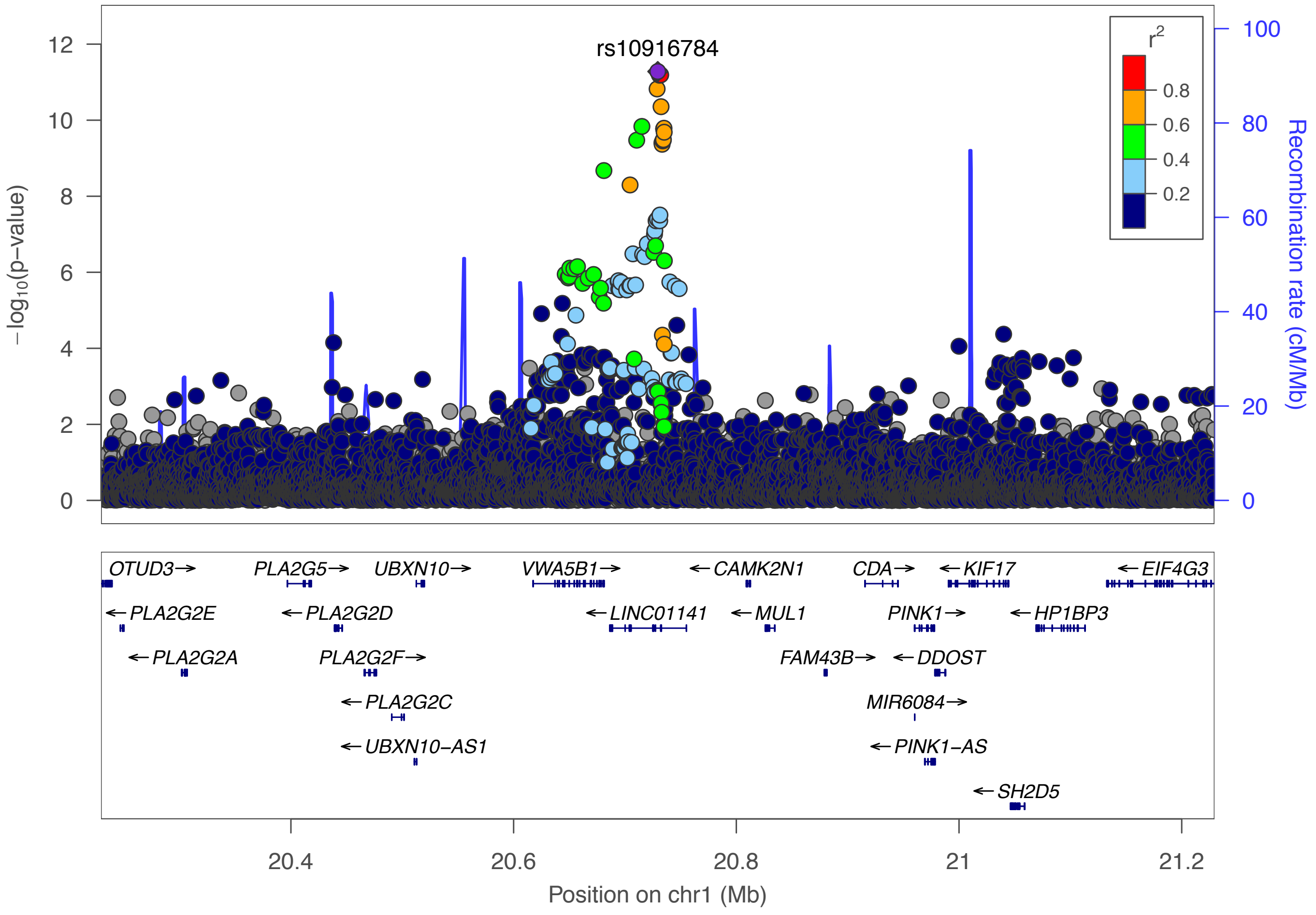

Supplementary Figure 24. Regional plots for rs10916784, showing association of SNPs with T2D for BMI adjusted model in South Asians only, and in South Asians and Europeans.

rs7531962 (chr1: 146746023)

South Asians only

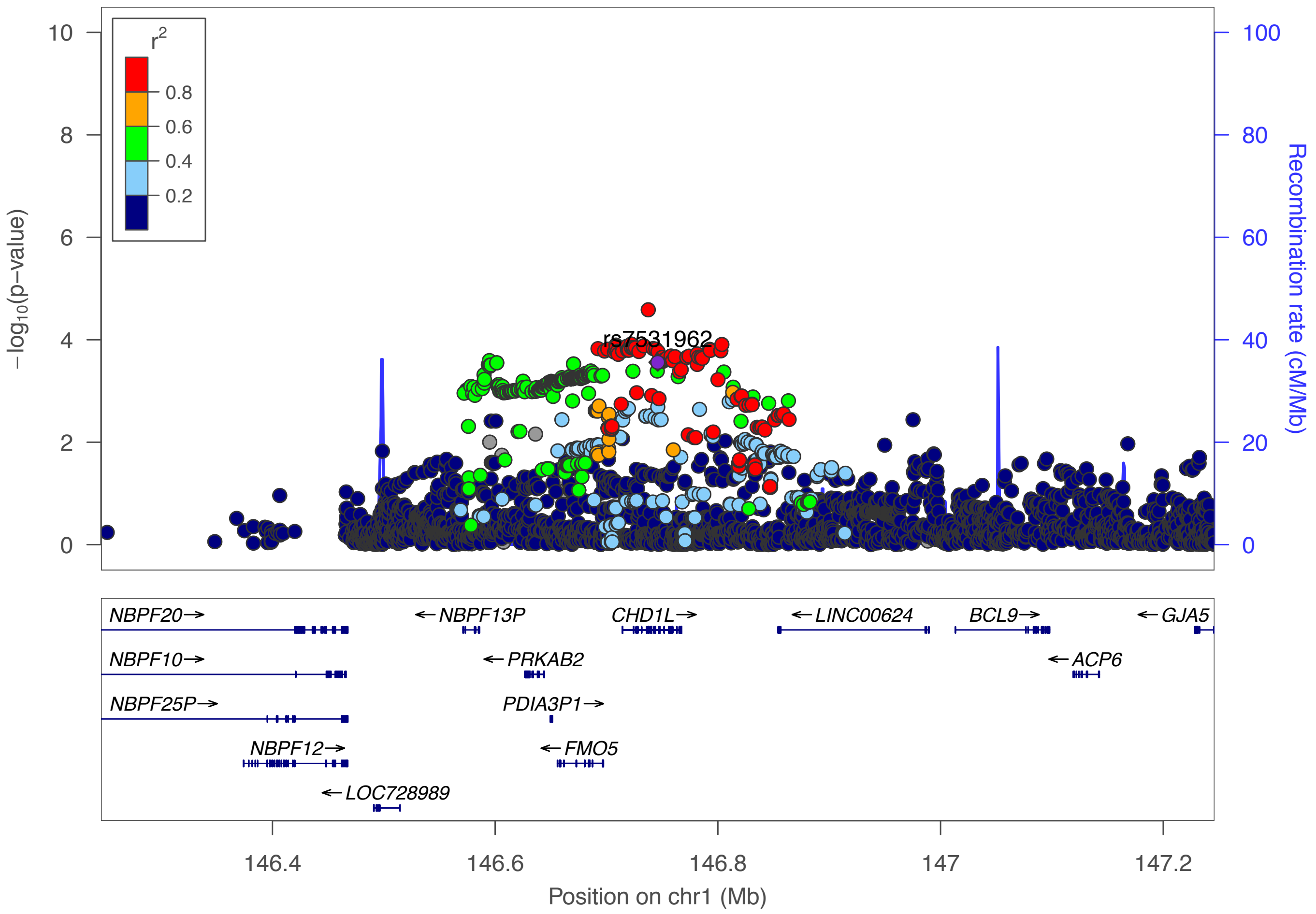

South Asians + Europeans

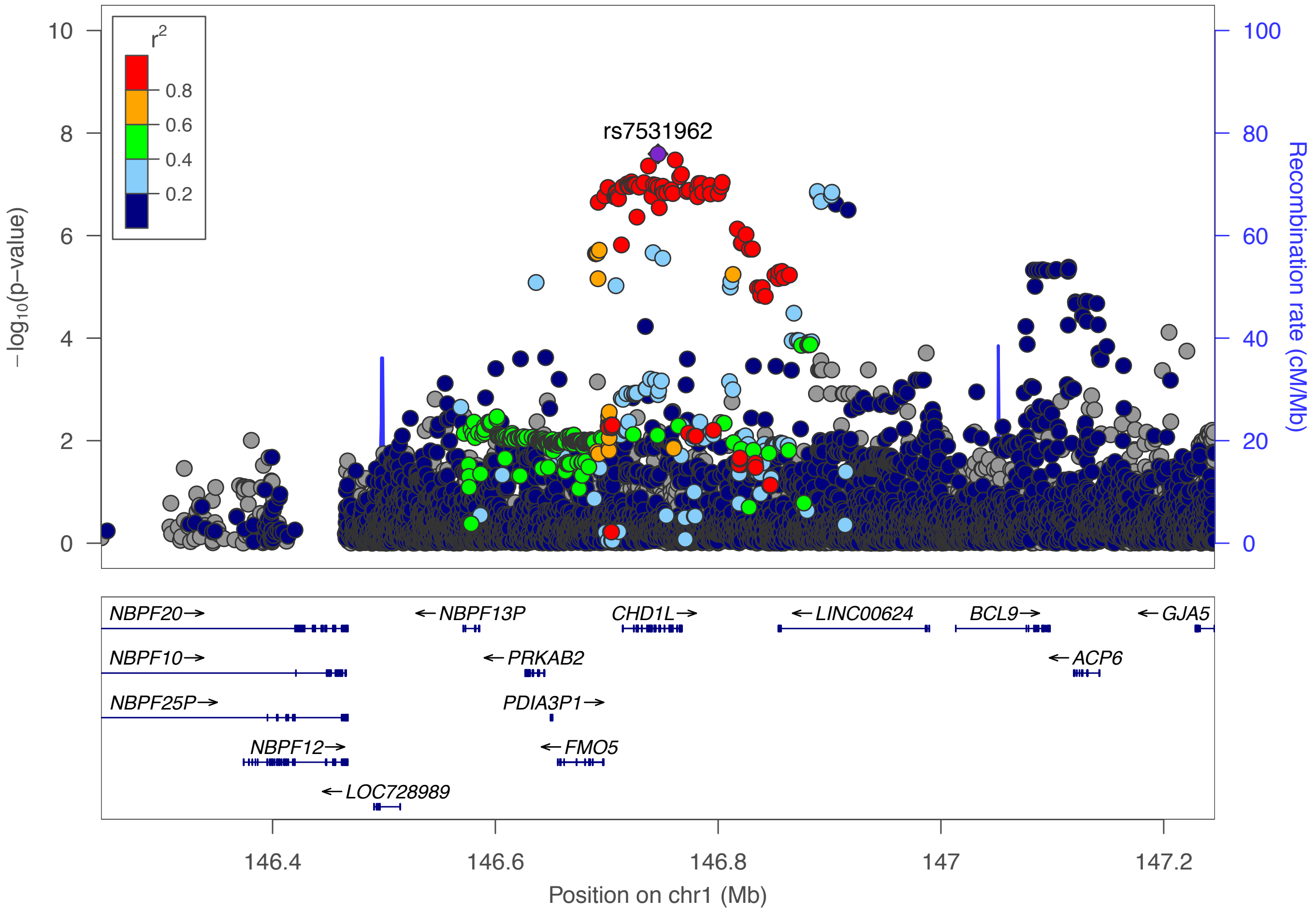

Supplementary Figure 25. Regional plots for rs7531962, showing association of SNPs with T2D for BMI adjusted model in South Asians only, and in South Asians and Europeans.

rs12463719 (chr2: 203450680)

South Asians only

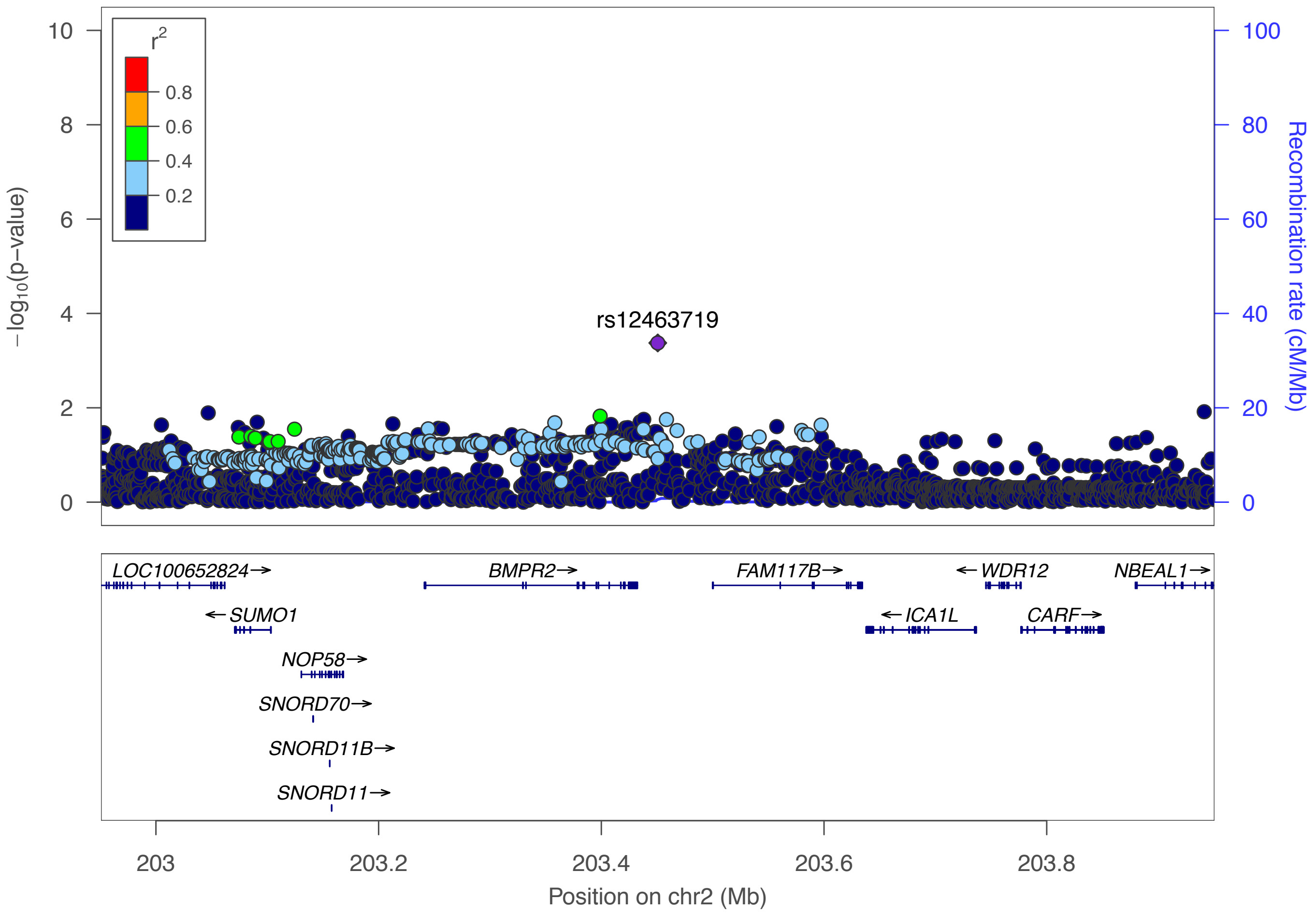

South Asians + Europeans

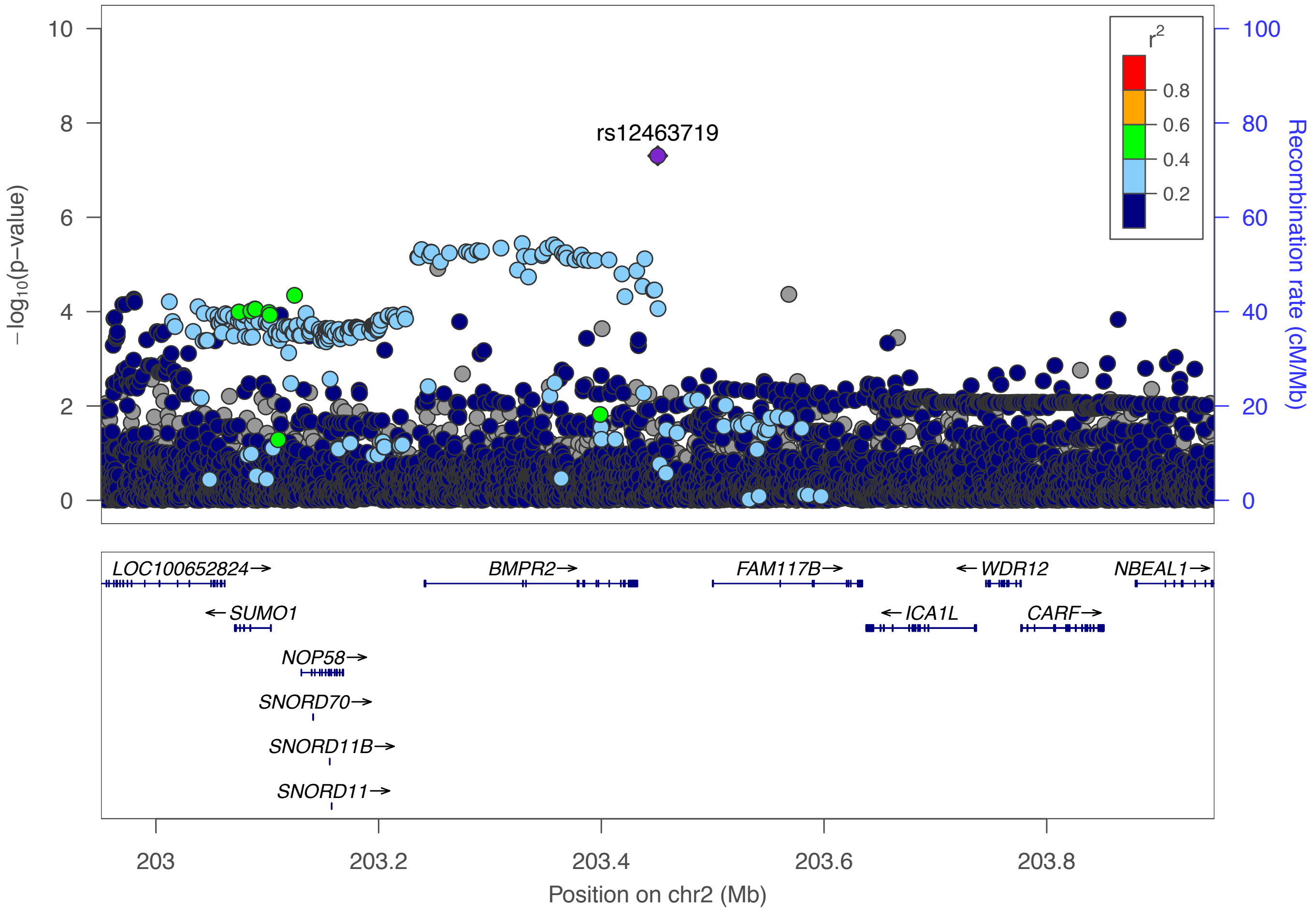

Supplementary Figure 26. Regional plots for rs12463719, showing association of SNPs with T2D for BMI adjusted model in South Asians only, and in South Asians and Europeans.

rs7432739 (chr3: 58364650)

South Asians only

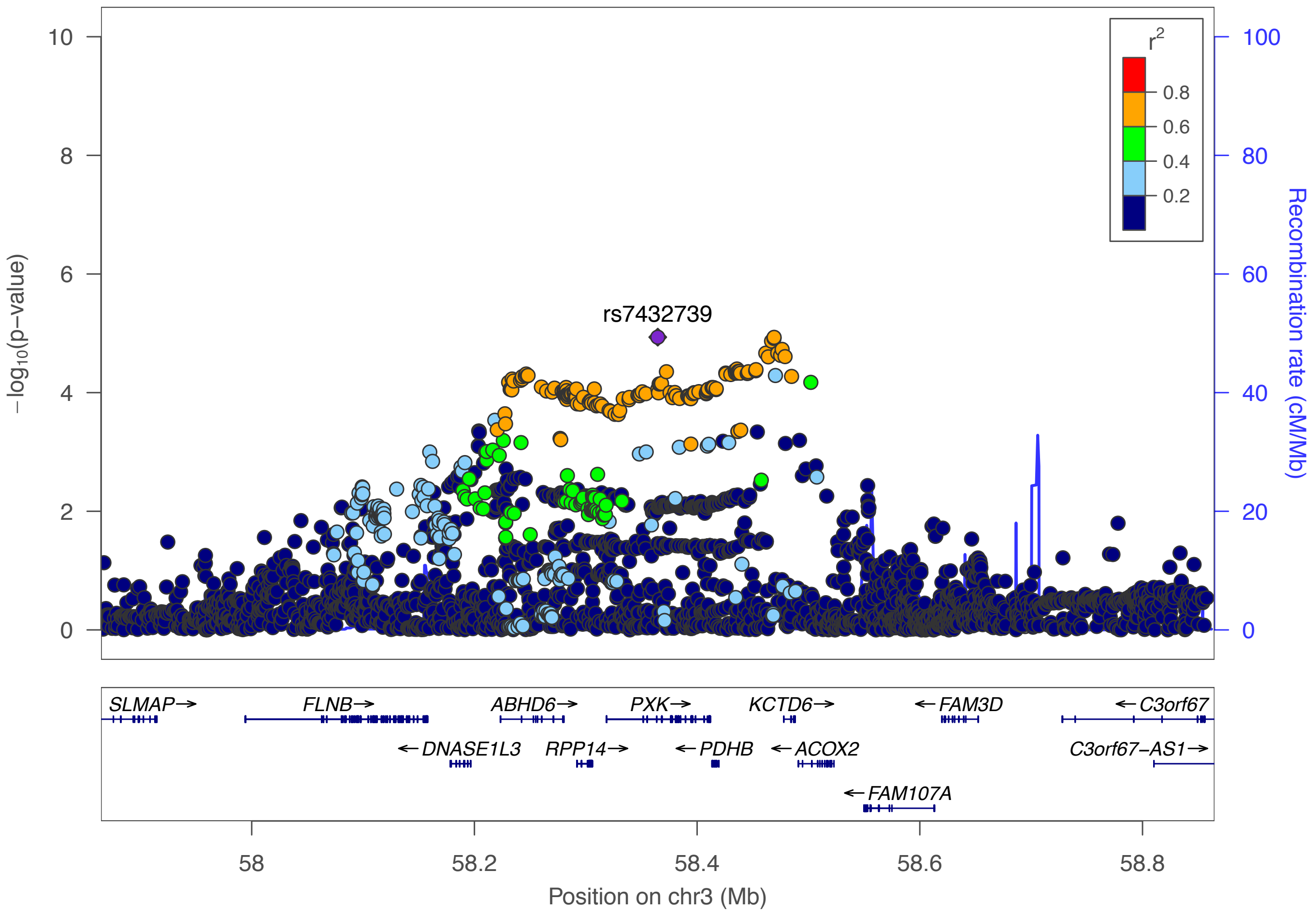

South Asians + Europeans

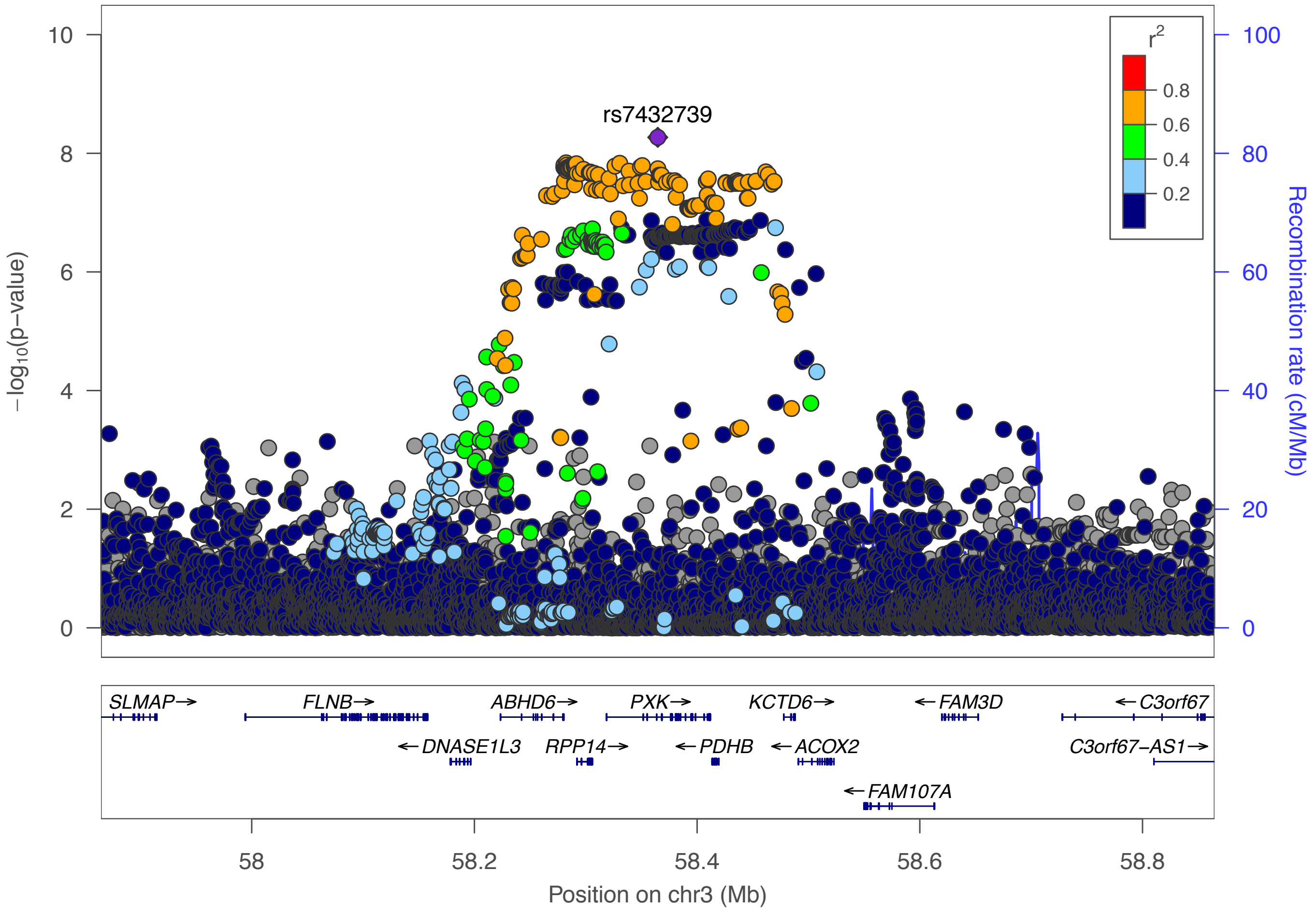

Supplementary Figure 27. Regional plots for rs7432739, showing association of SNPs with T2D for BMI adjusted model in South Asians only, and in South Asians and Europeans.

rs7626079 (chr3: 66427259)

South Asians only

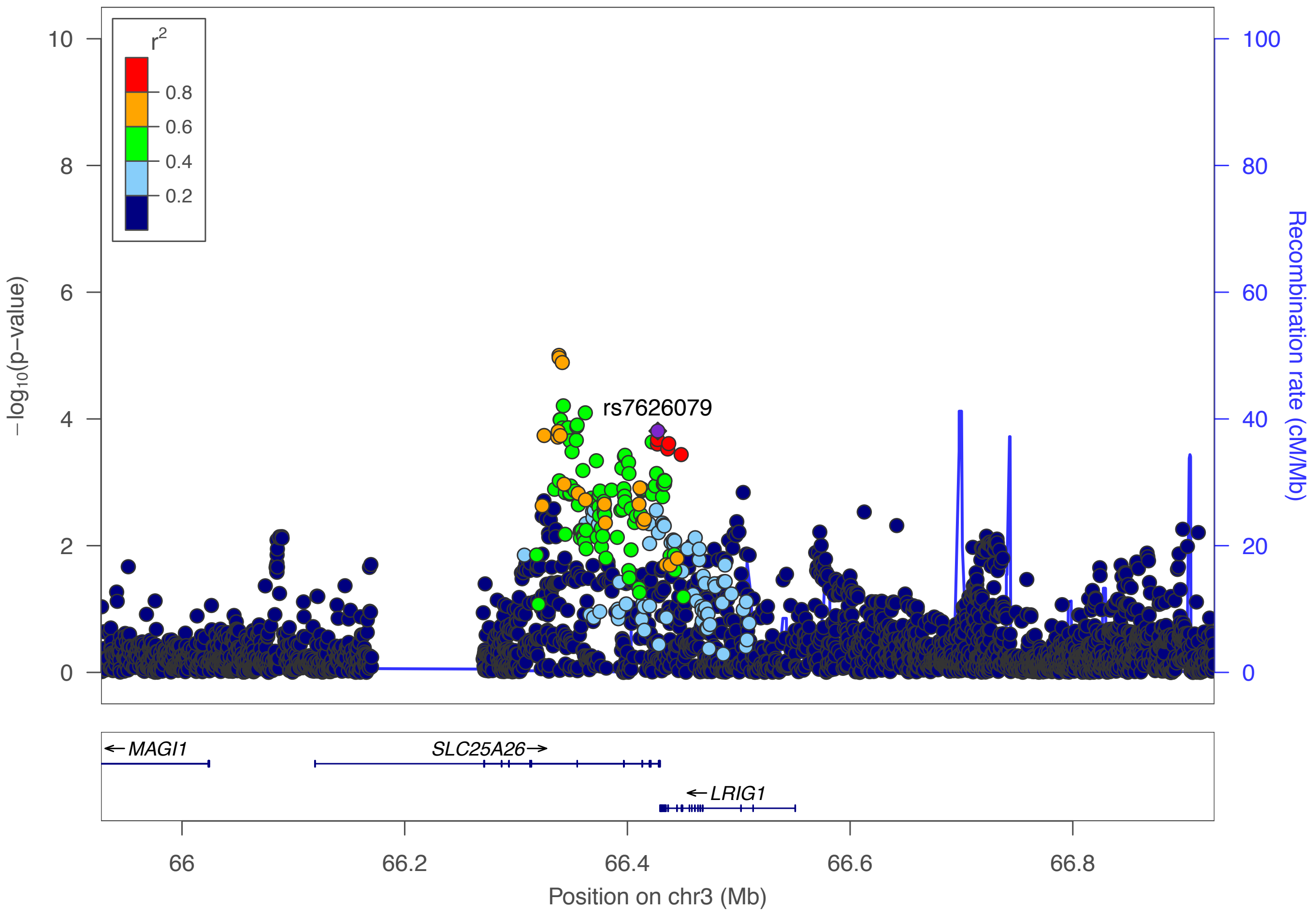

South Asians + Europeans

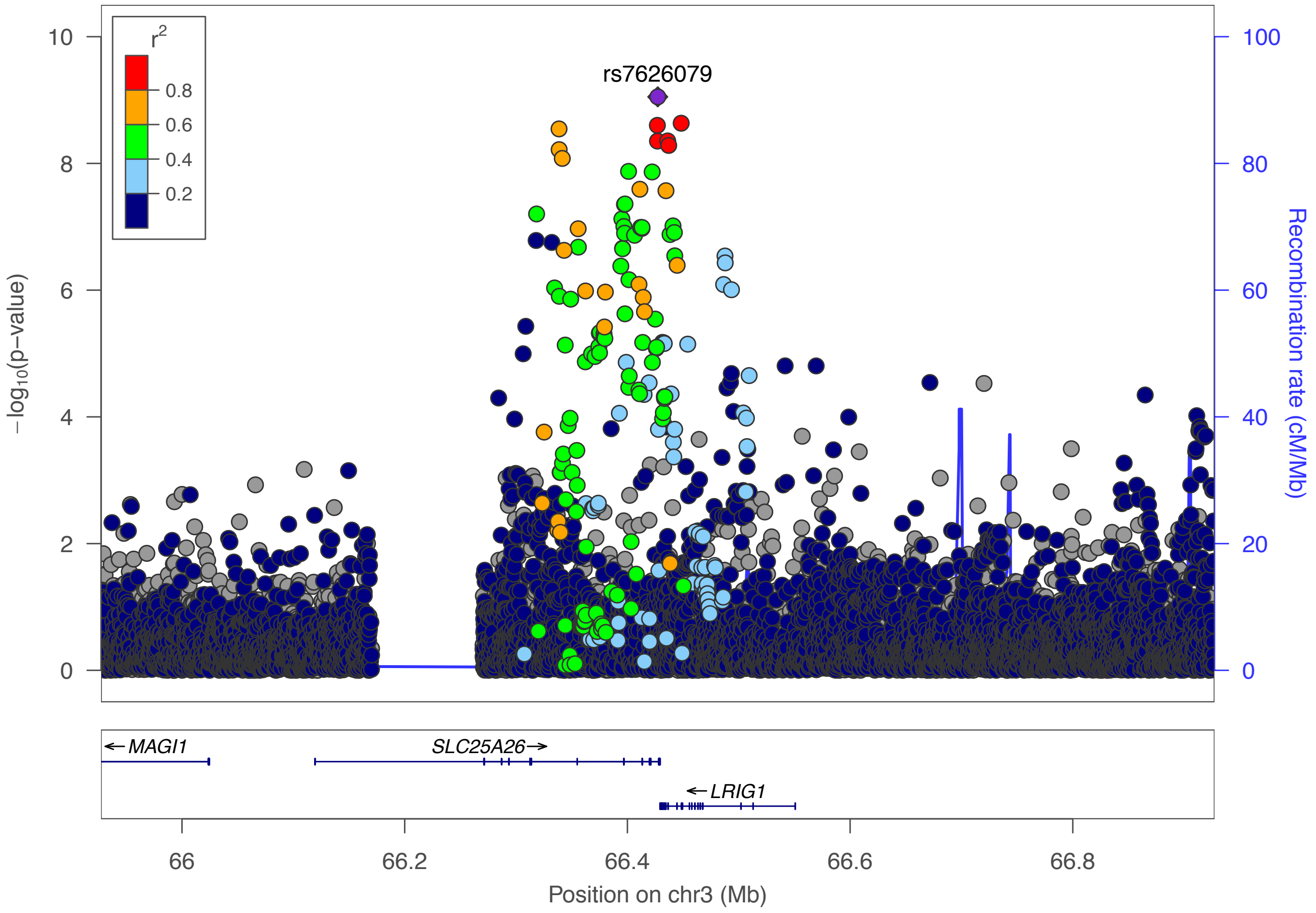

Supplementary Figure 28. Regional plots for rs7626079, showing association of SNPs with T2D for BMI adjusted model in South Asians only, and in South Asians and Europeans.

rs62366901 (chr5: 49930286)

South Asians only

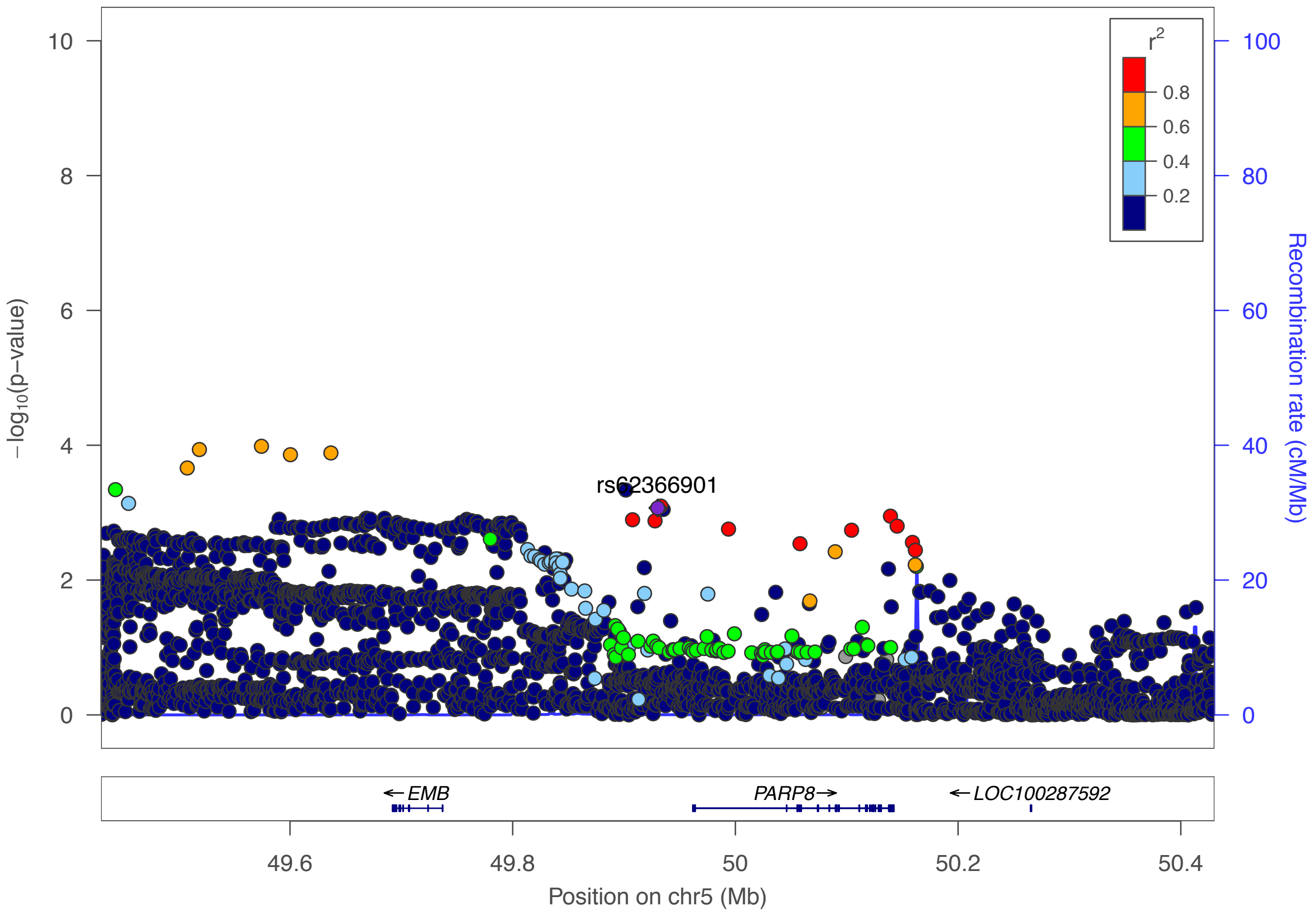

South Asians + Europeans

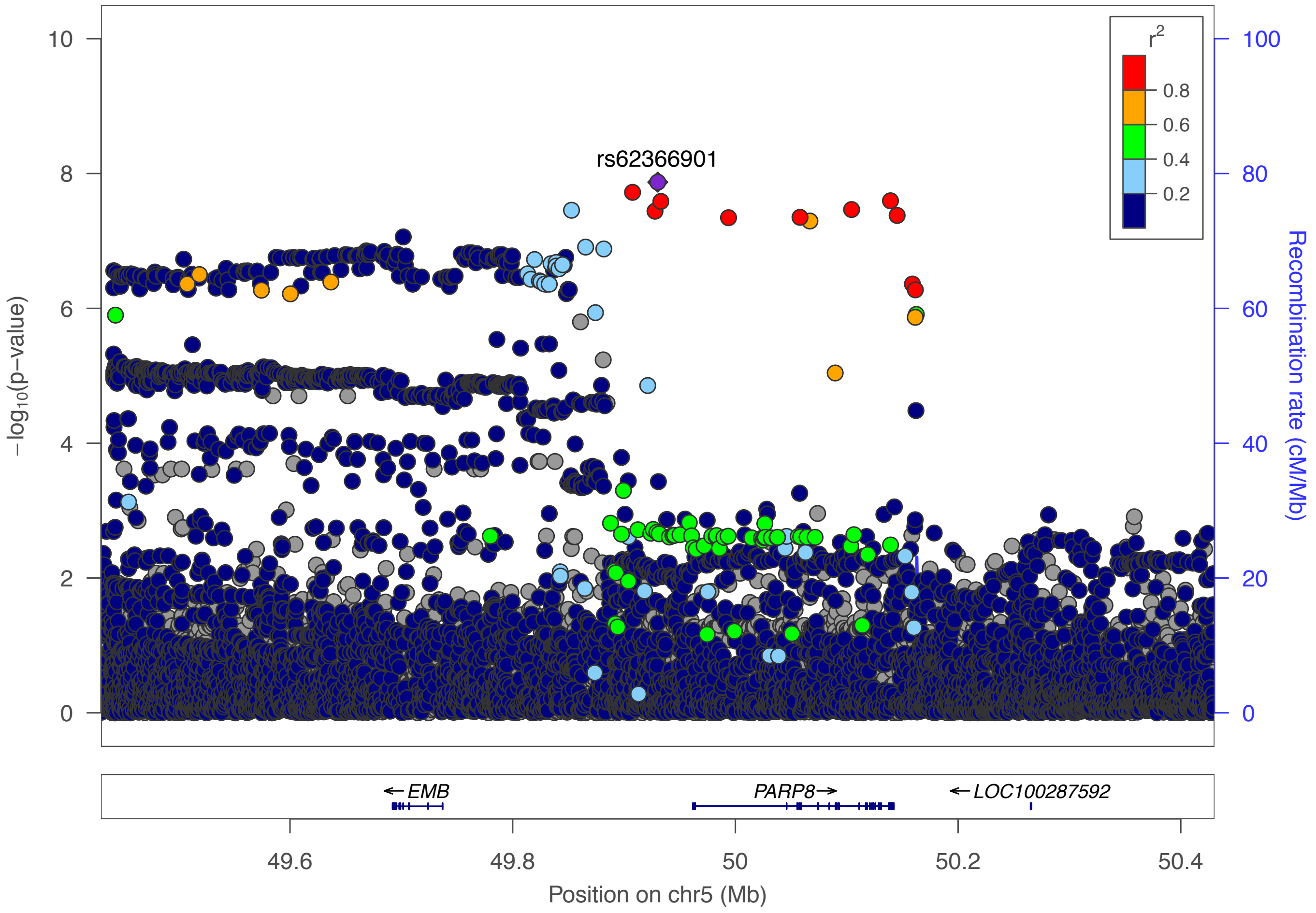

Supplementary Figure 29. Regional plots for rs62366901 showing association of SNPs with T2D for BMI adjusted model in South Asians only, and in South Asians and Europeans.

rs74790763 (chr5: 122675214)

South Asians only

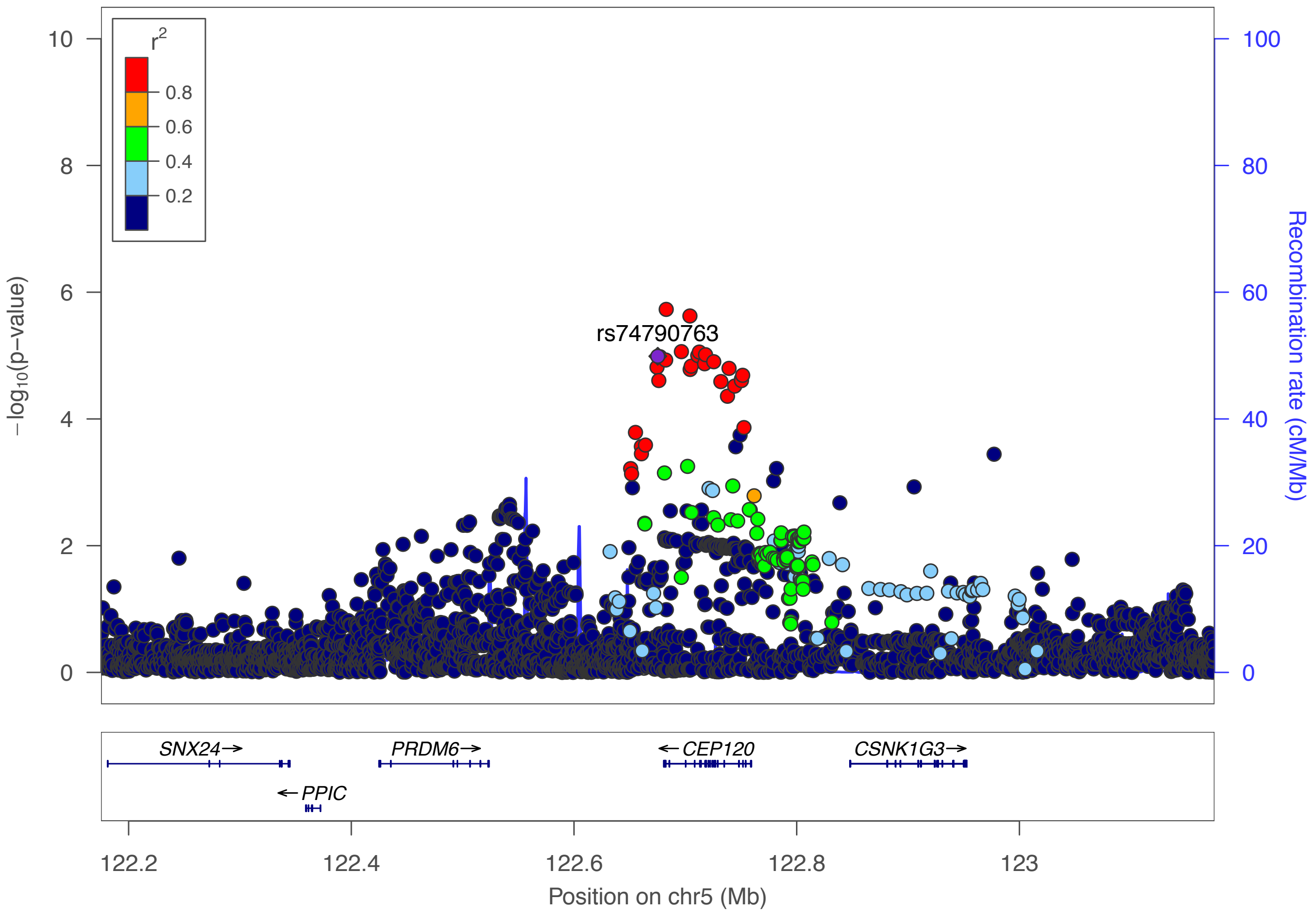

South Asians + Europeans

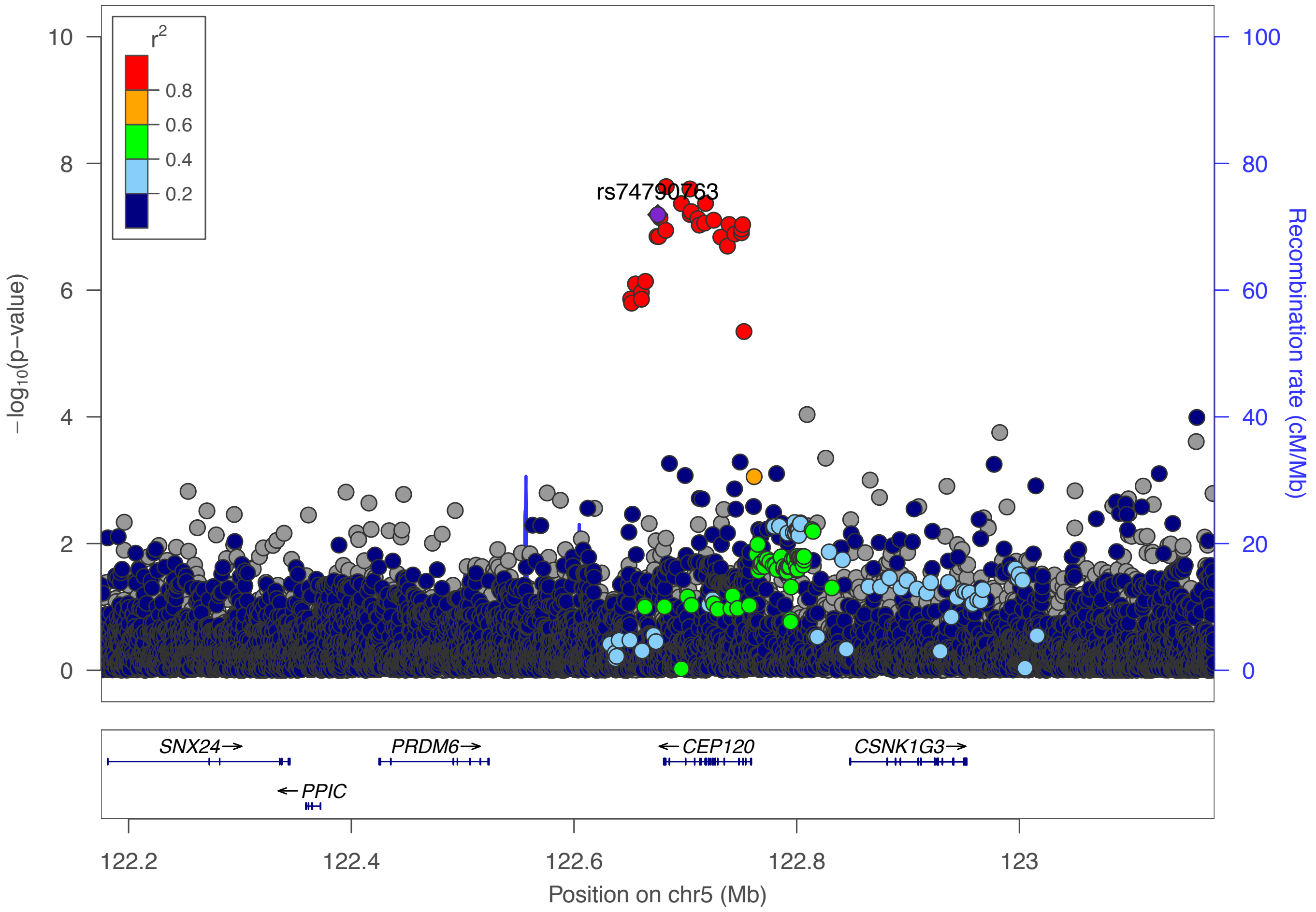

Supplementary Figure 30. Regional plots for rs74790763 showing association of SNPs with T2D for BMI adjusted model in South Asians only, and in South Asians and Europeans.

rs7765207 (chr6: 138858054)

South Asians only

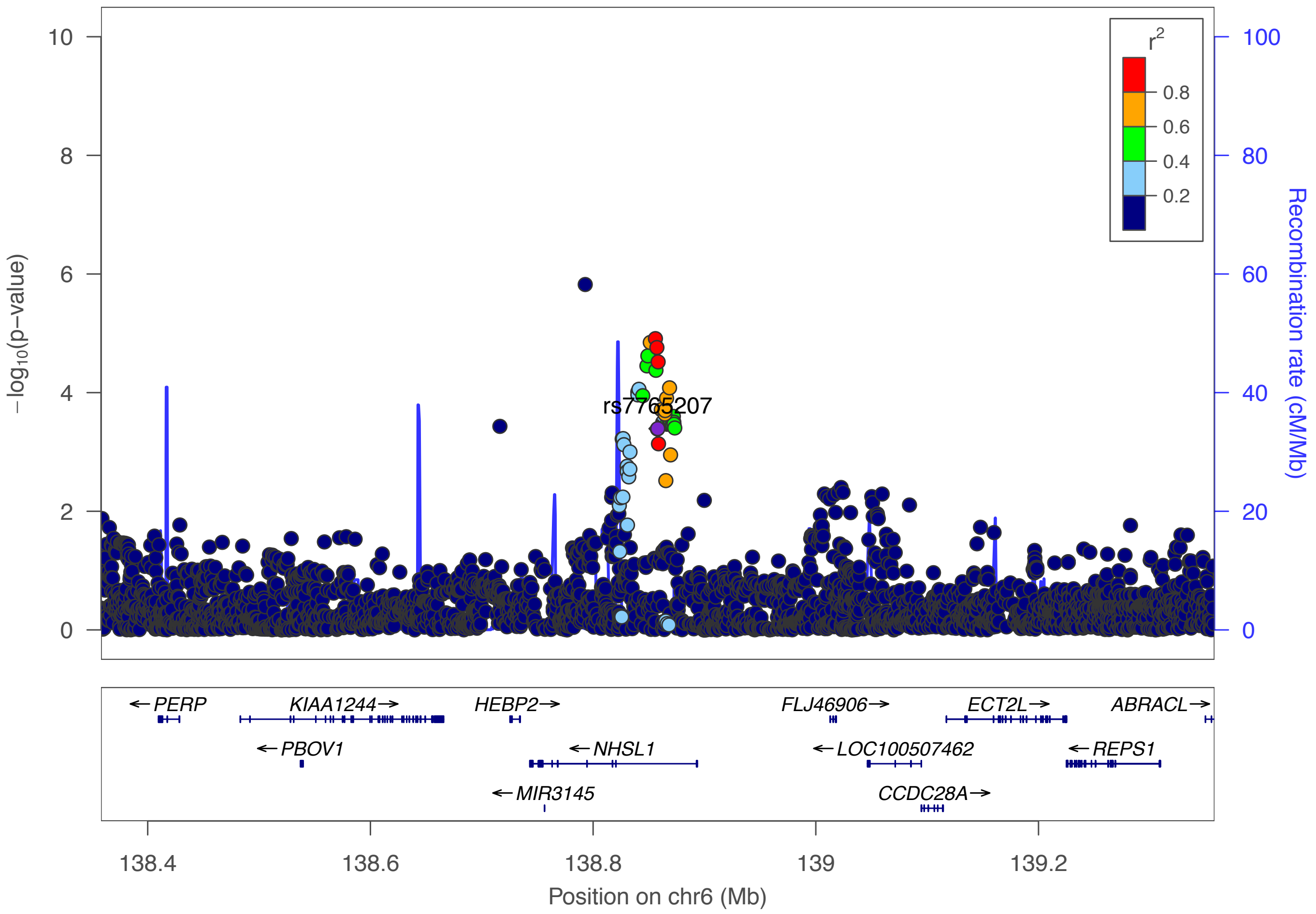

South Asians + Europeans

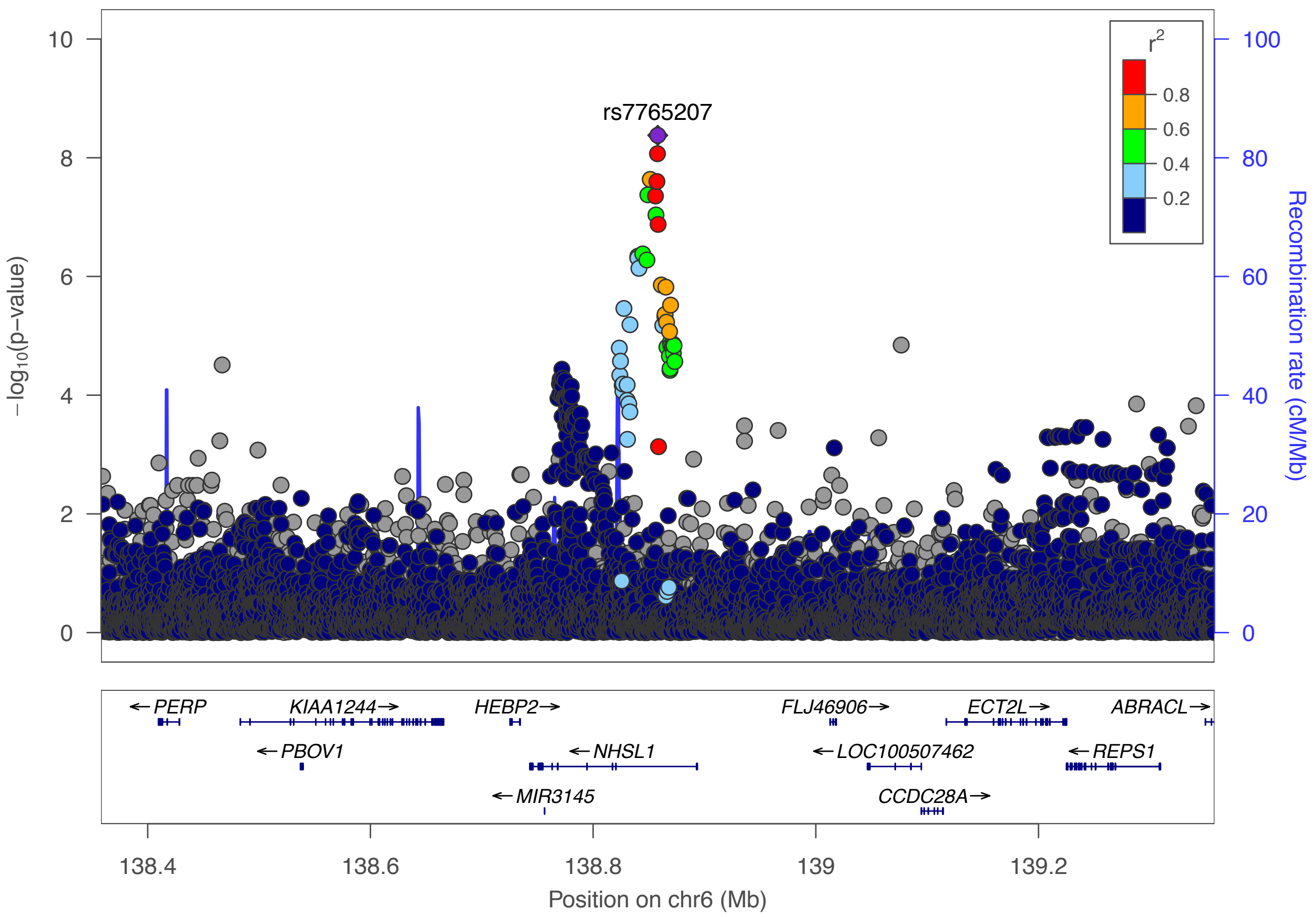

Supplementary Figure 31. Regional plots for rs7765207 showing association of SNPs with T2D for BMI adjusted model in South Asians only, and in South Asians and Europeans.

rs73689877 (chr7: 40905274)

South Asians only

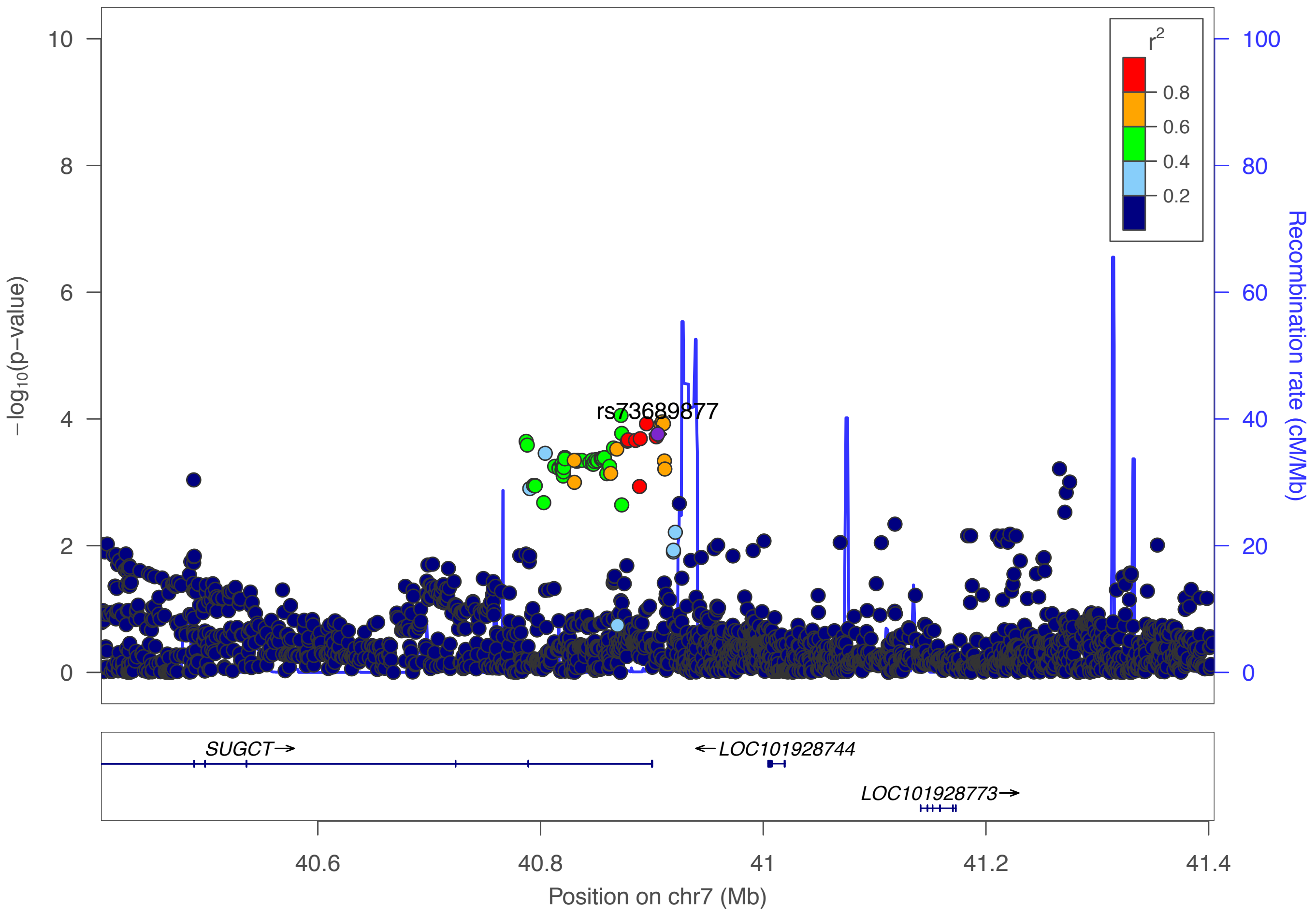

South Asians + Europeans

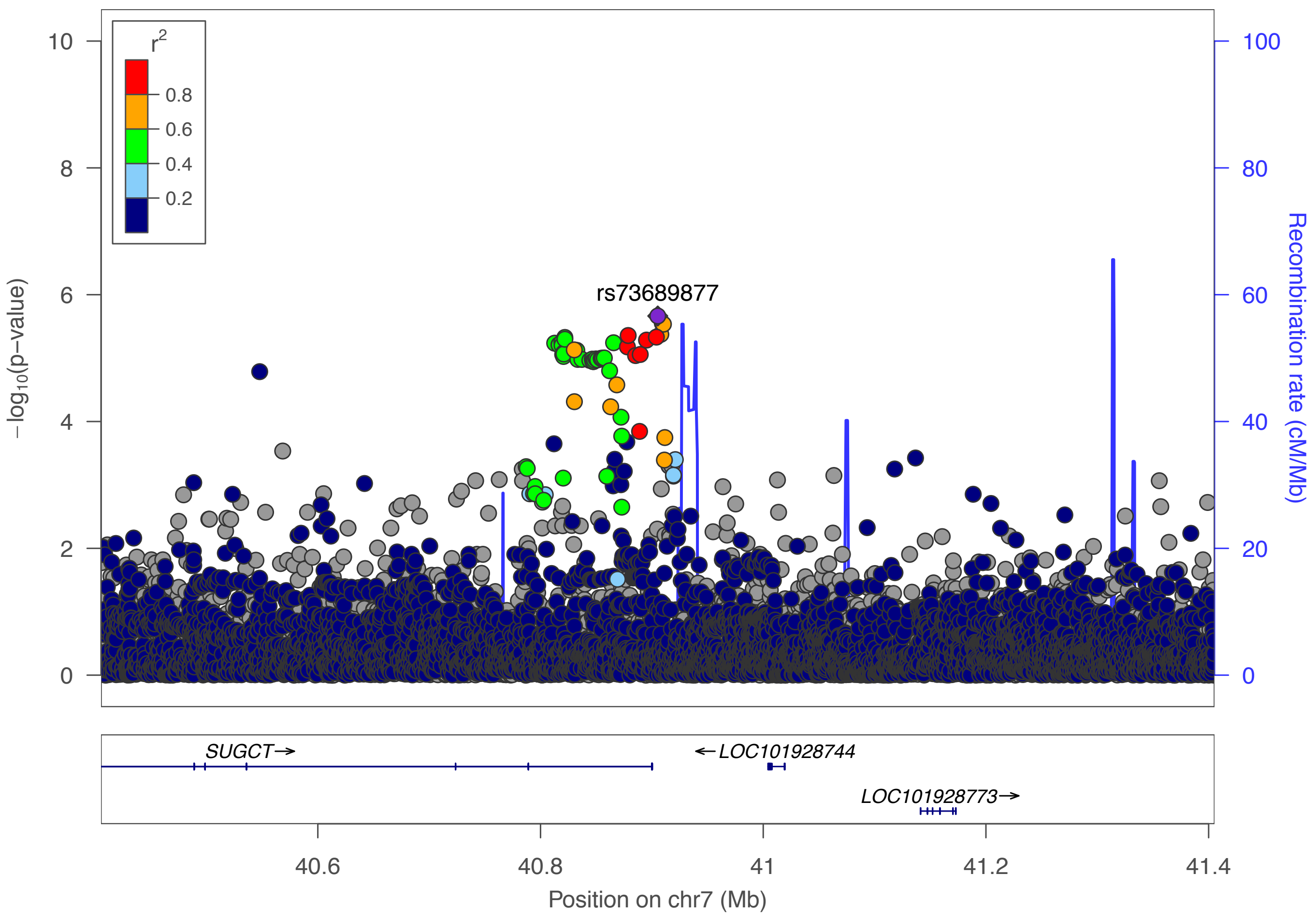

Supplementary Figure 32. Regional plots for rs73689877 showing association of SNPs with T2D for BMI adjusted model in South Asians only, and in South Asians and Europeans.

rs2980766 (chr8: 8318095)

South Asians only

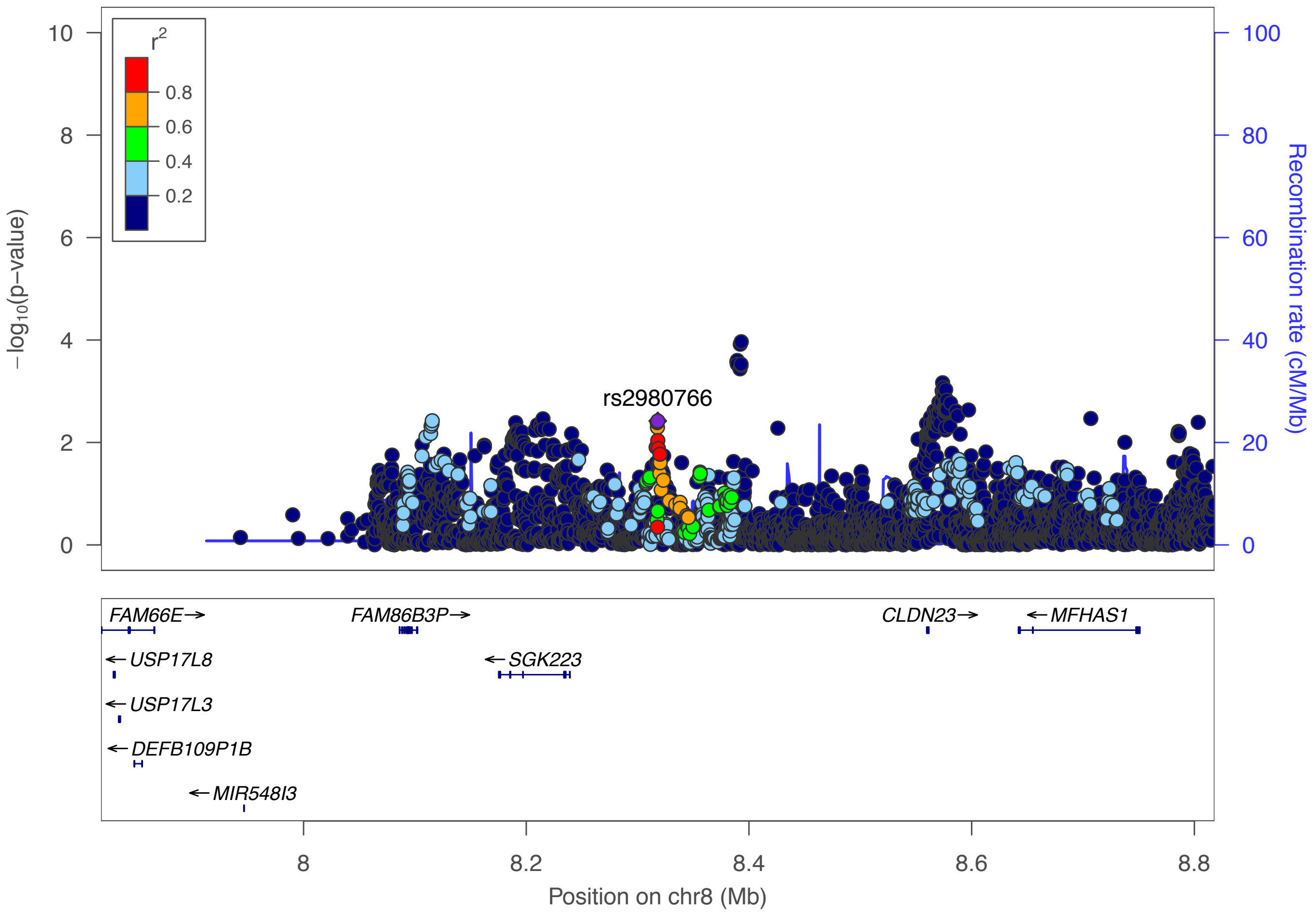

South Asians + Europeans

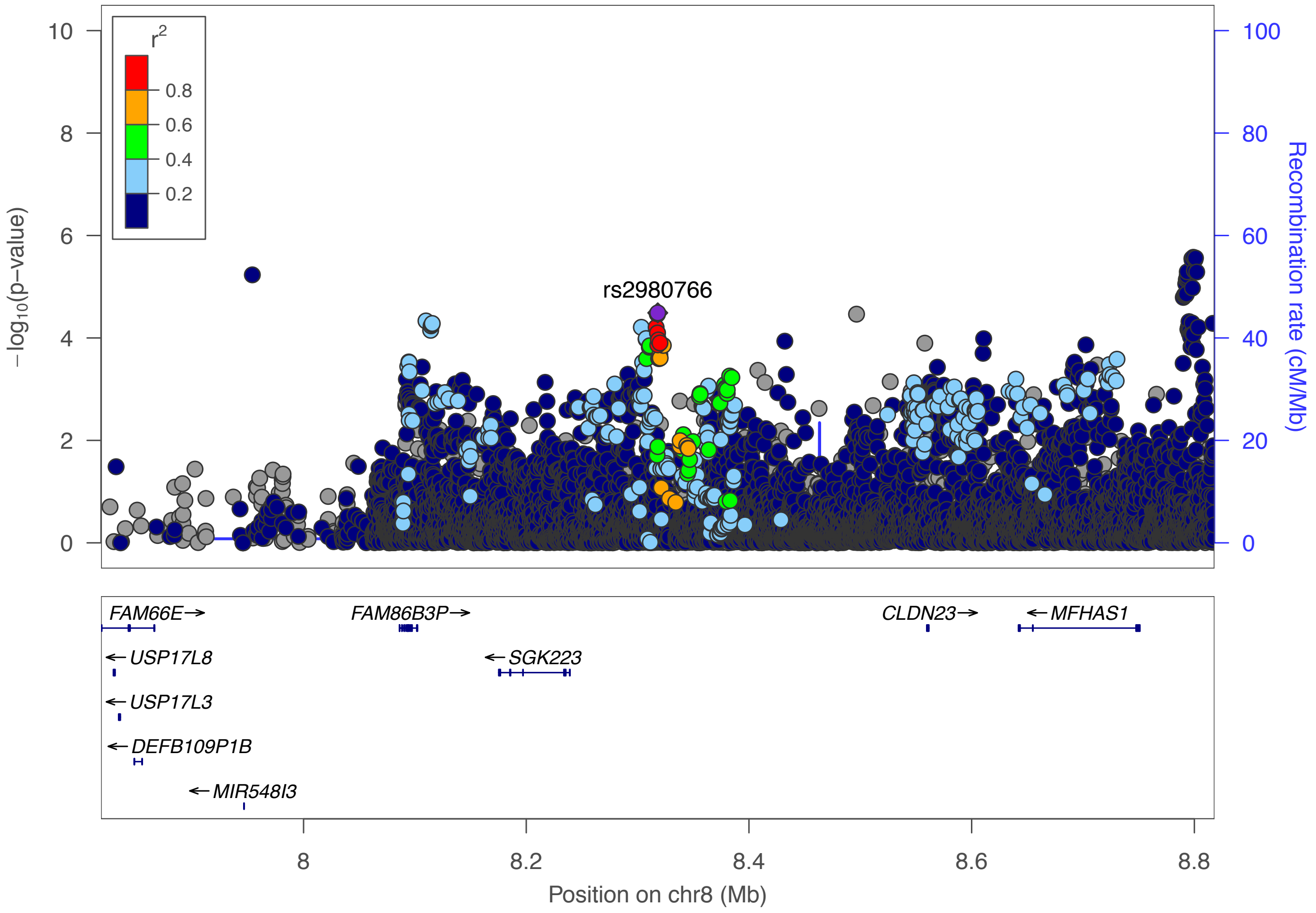

Supplementary Figure 33. Regional plots for rs2980766 showing association of SNPs with T2D for BMI adjusted model in South Asians only, and in South Asians and Europeans.

rs62486442 (chr8: 12623463)

South Asians only

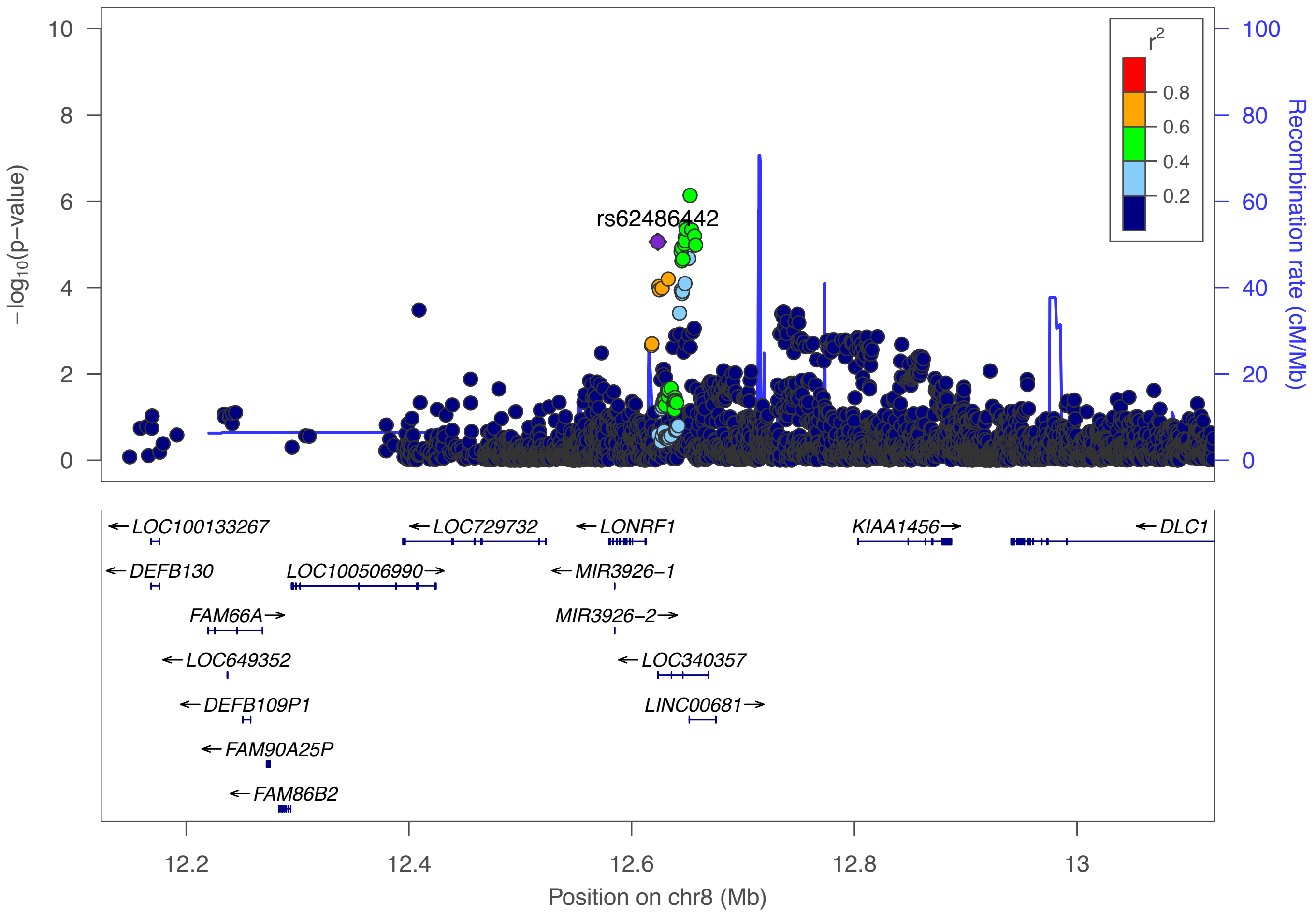

South Asians + Europeans

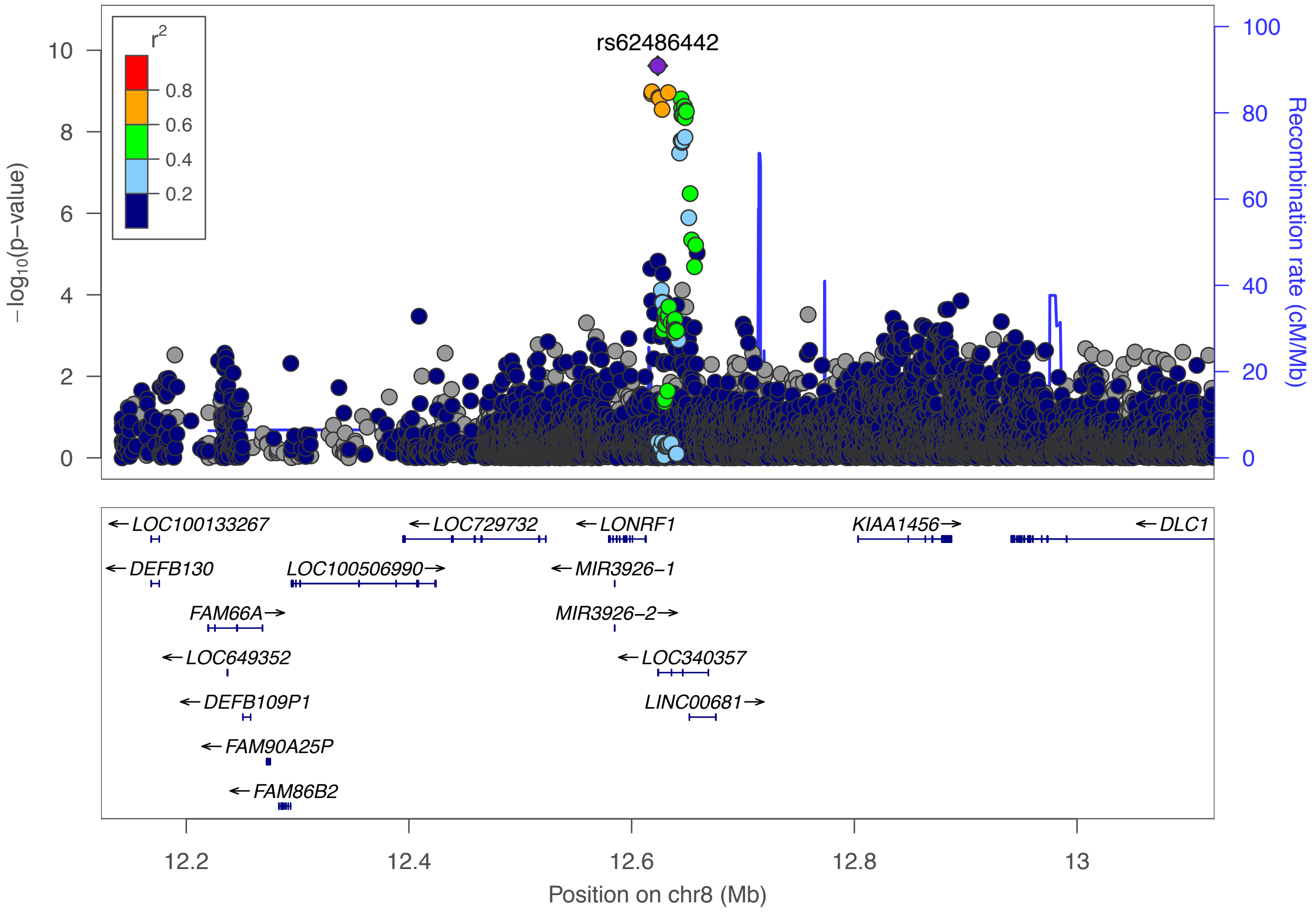

Supplementary Figure 34. Regional plots for rs62486442 showing association of SNPs with T2D for BMI adjusted model in South Asians only, and in South Asians and Europeans.

rs13257283 (chr8: 105608497)

South Asians only

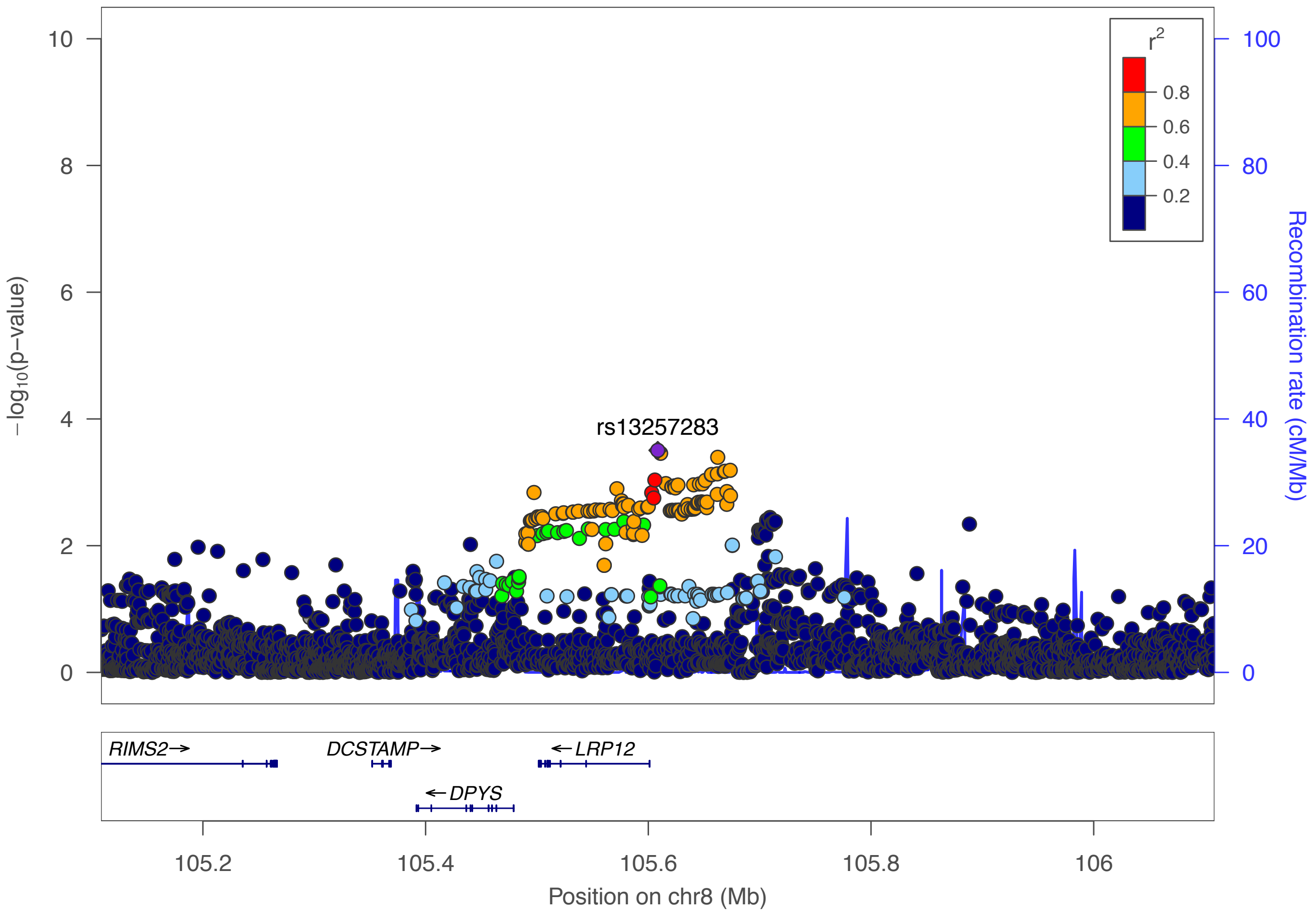

South Asians + Europeans

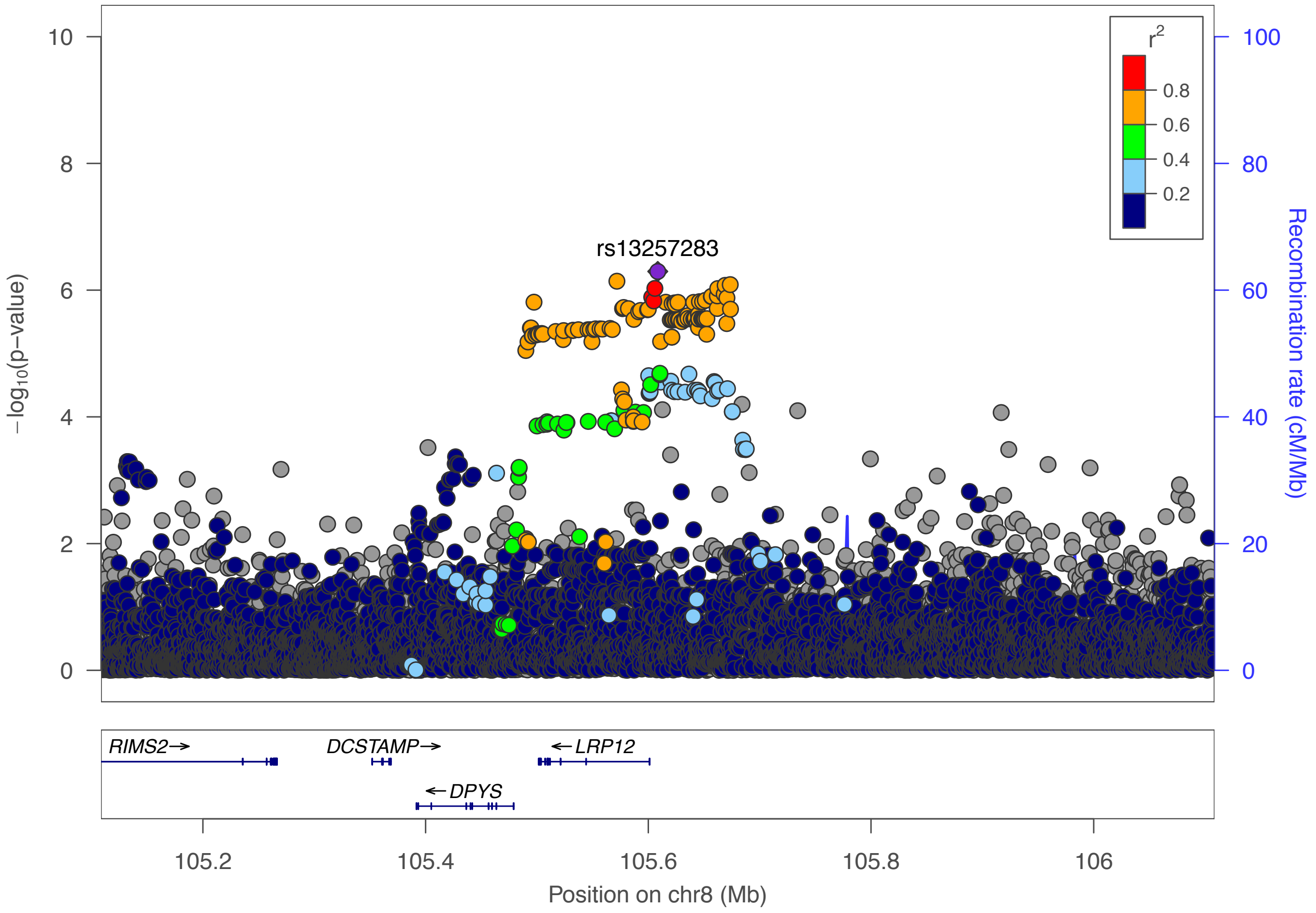

Supplementary Figure 35. Regional plots for rs13257283 showing association of SNPs with T2D for BMI adjusted model in South Asians only, and in South Asians and Europeans.

rs2488597 (chr9: 125992073)

South Asians only

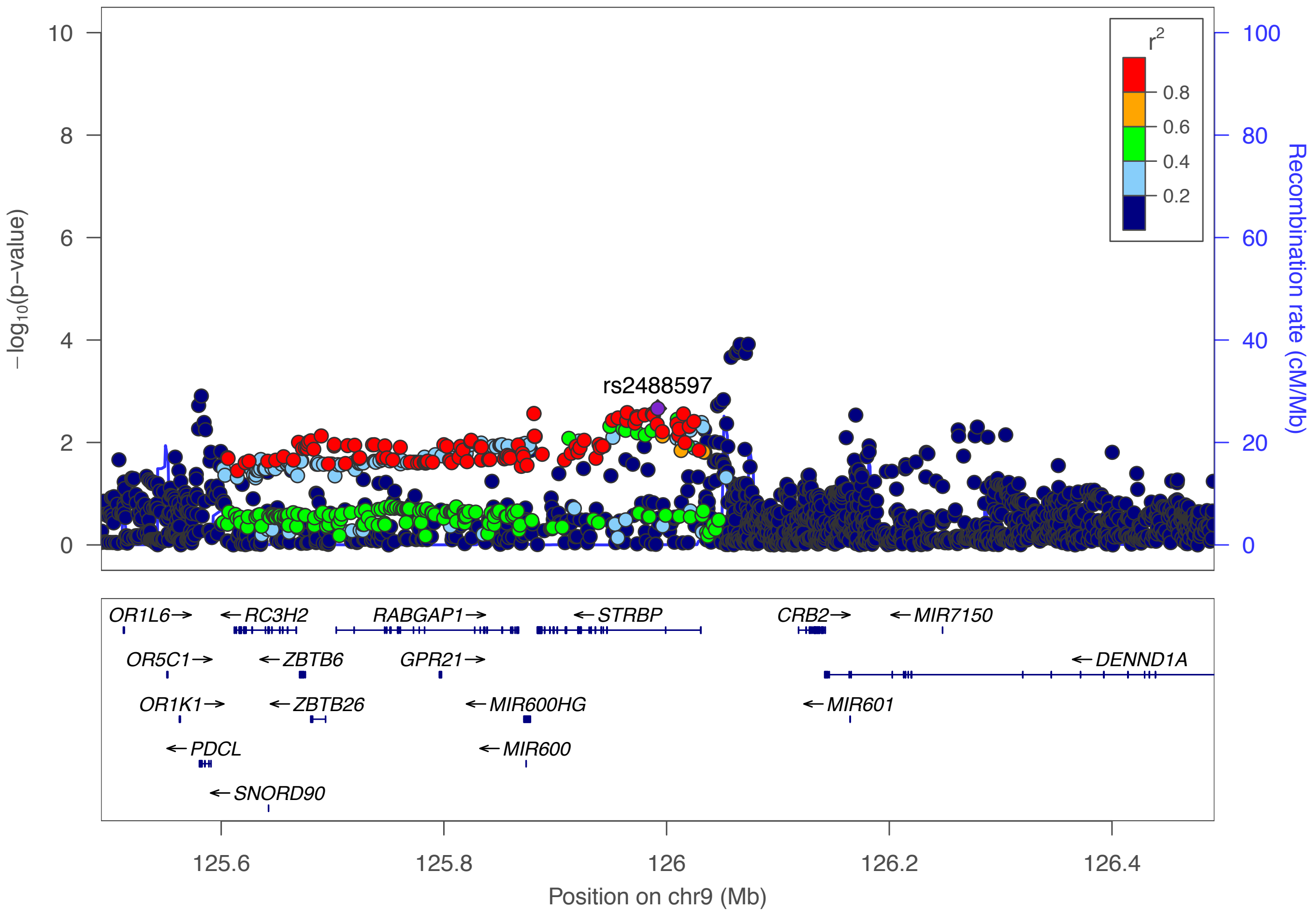

South Asians + Europeans

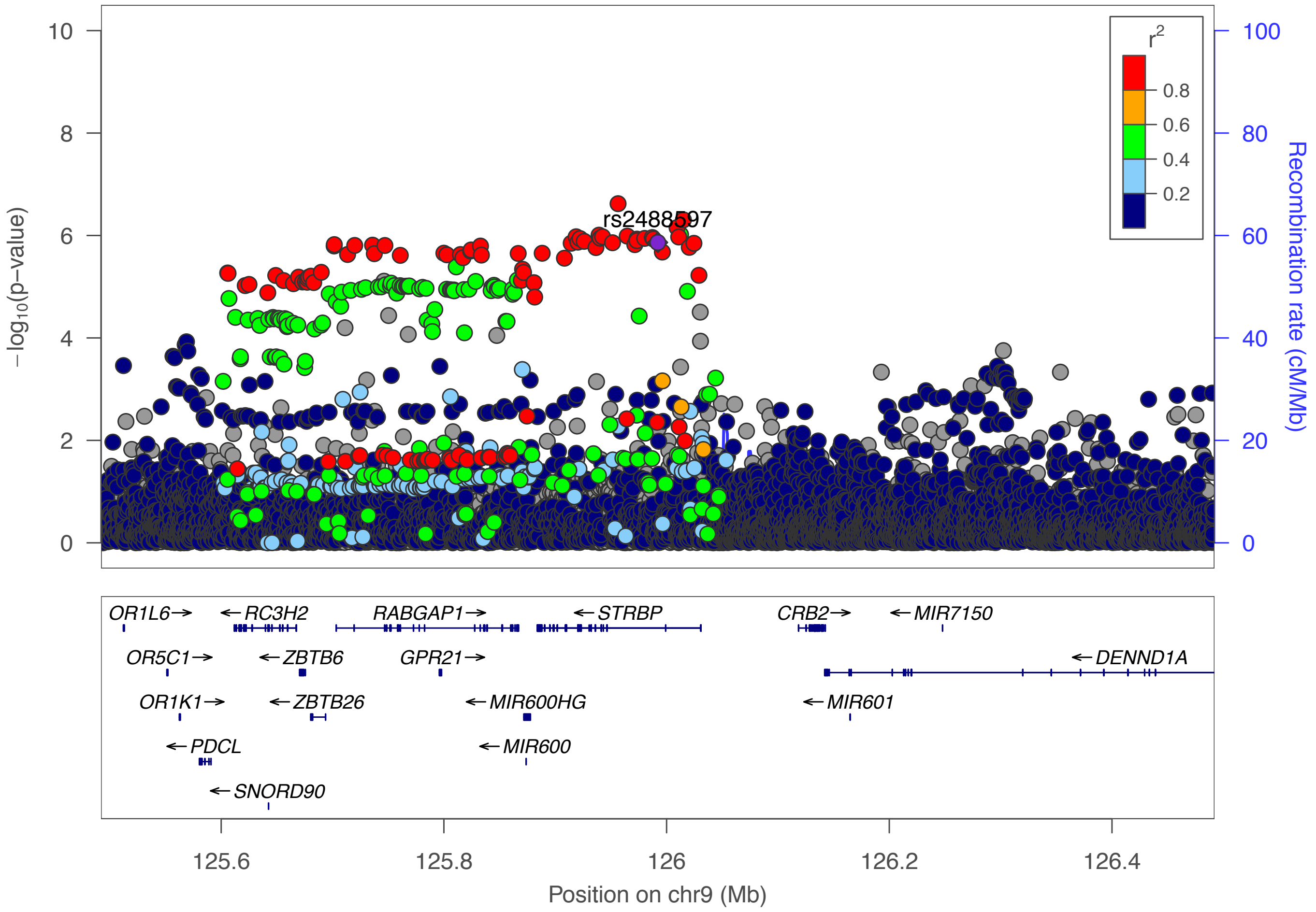

Supplementary Figure 36. Regional plots for rs2488597 showing association of SNPs with T2D for BMI adjusted model in South Asians only, and in South Asians and Europeans.

rs2114824 (chr10: 88119015)

South Asians only

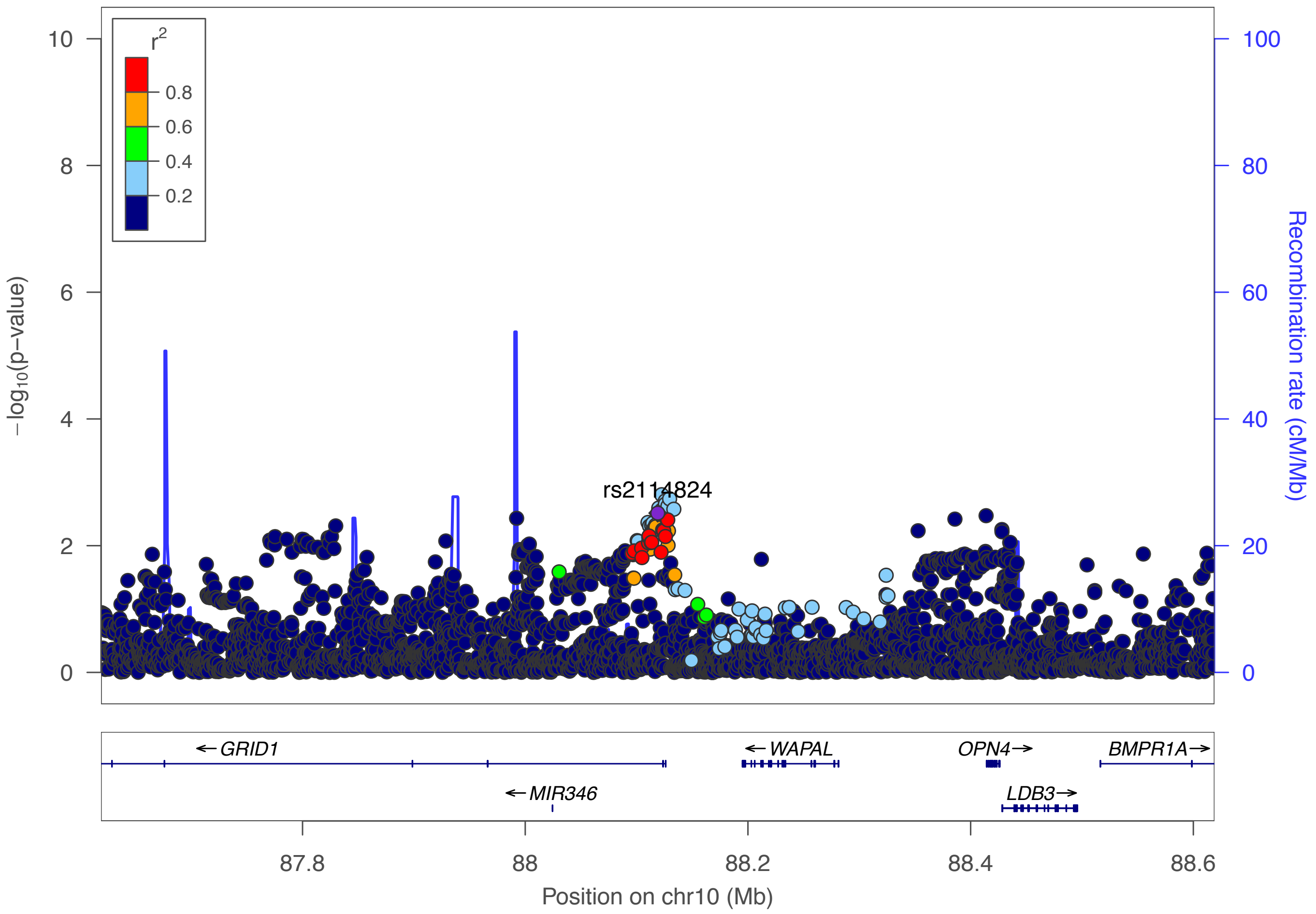

South Asians + Europeans

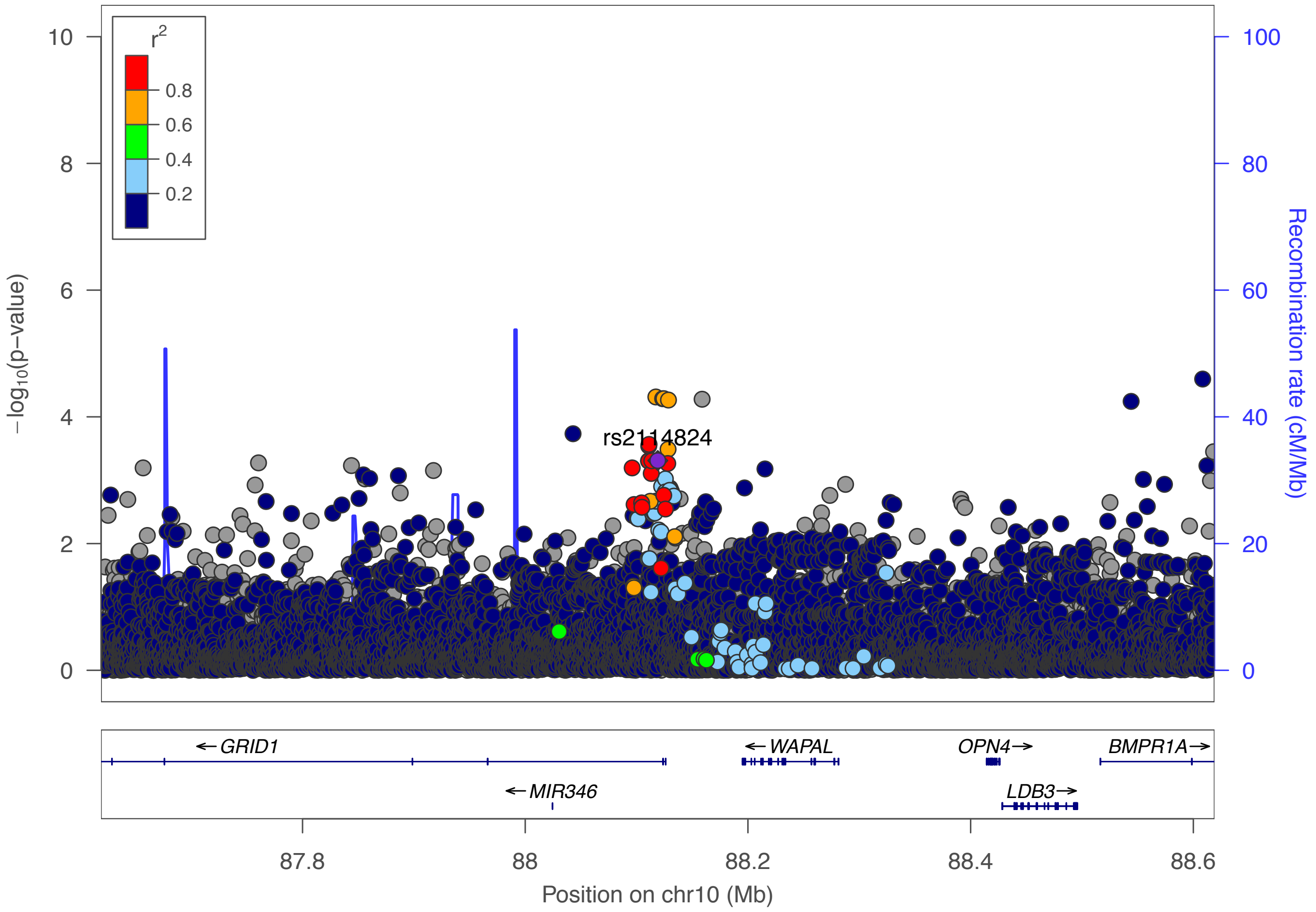

Supplementary Figure 37. Regional plots for rs2114824 showing association of SNPs with T2D for BMI adjusted model in South Asians only, and in South Asians and Europeans.

rs10748694 (chr10:99056190)

South Asians only

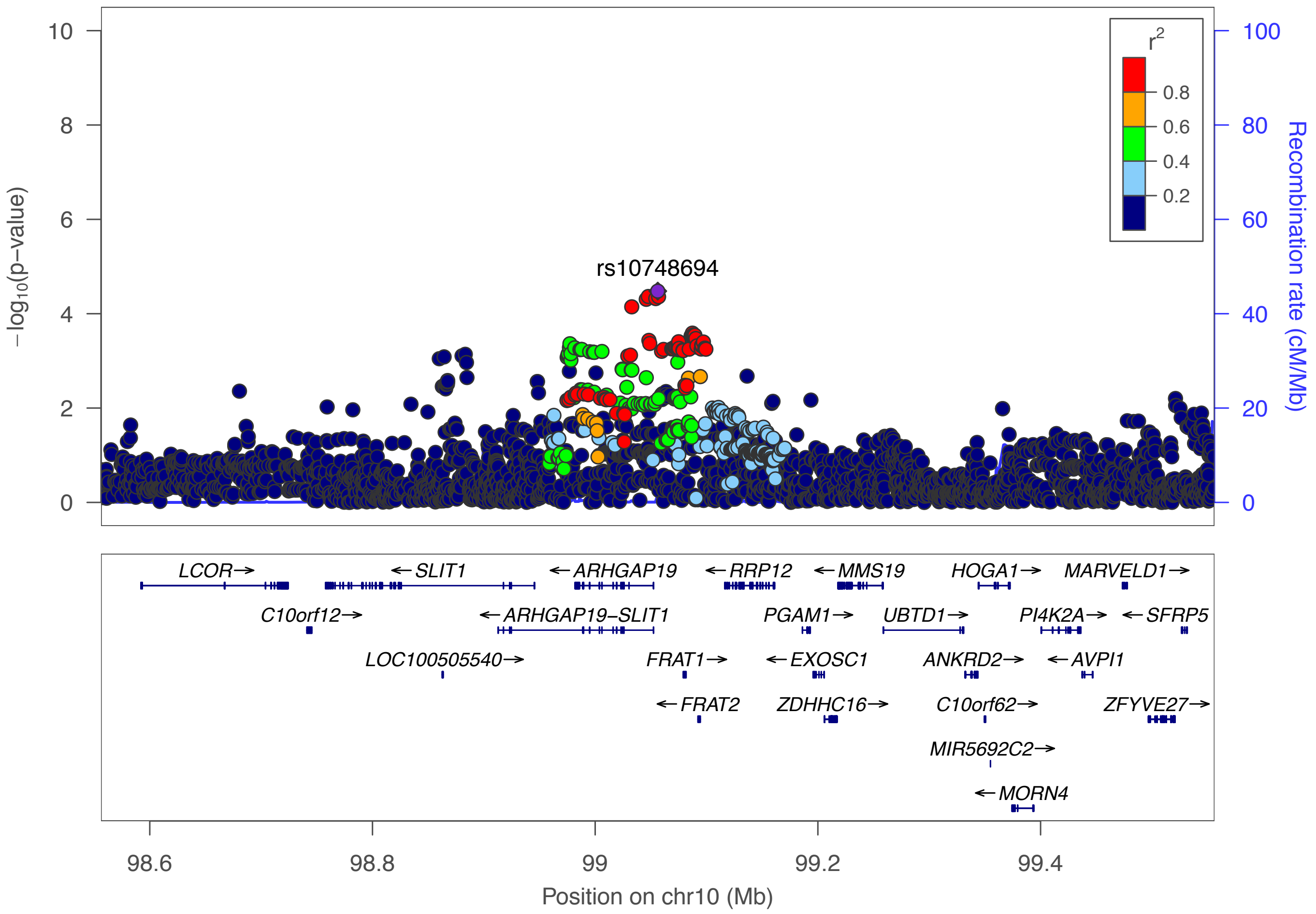

South Asians + Europeans

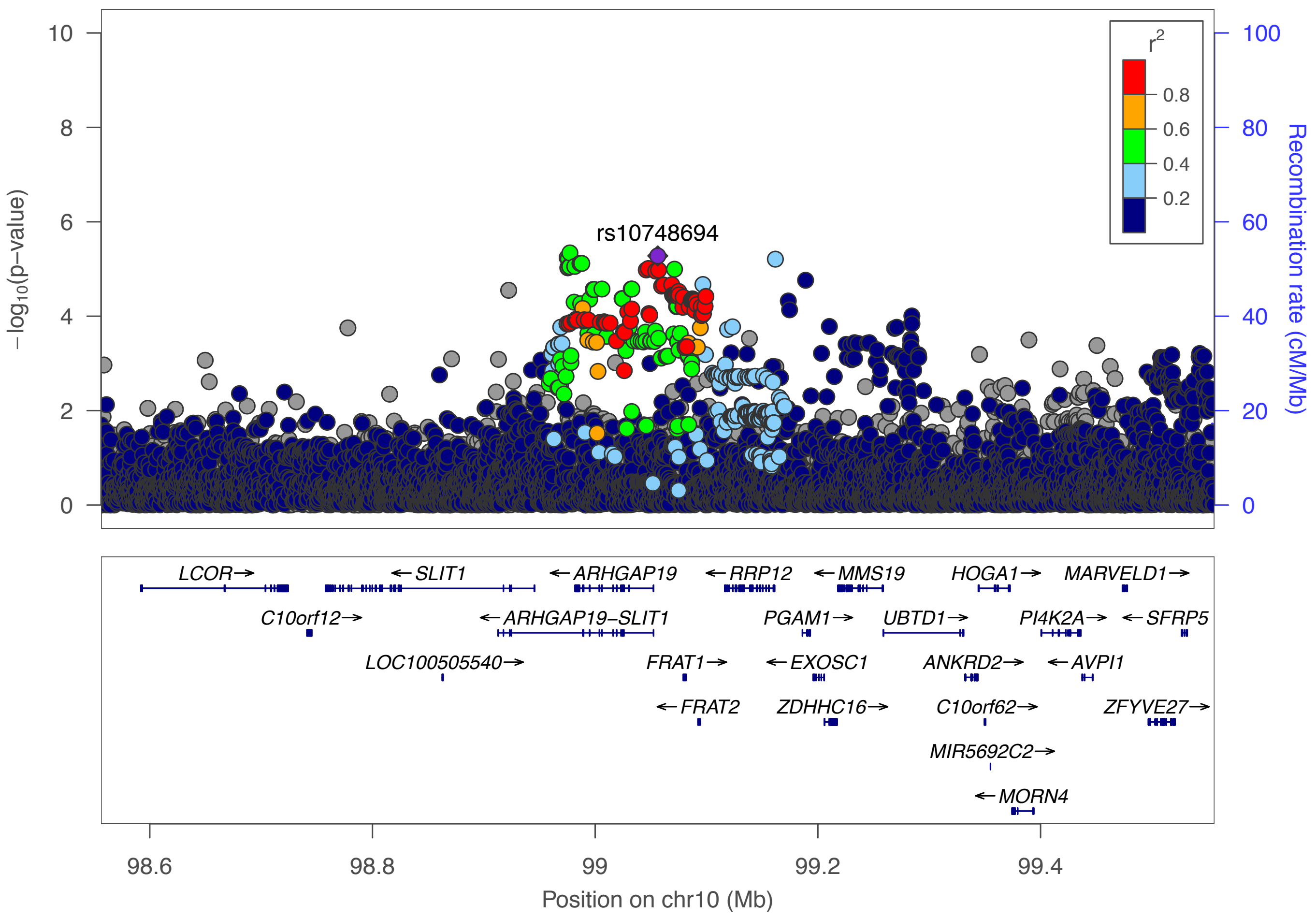

Supplementary Figure 38. Regional plots for rs10748694 showing association of SNPs with T2D for BMI adjusted model in South Asians only, and in South Asians and Europeans.

rs7123361 (chr11:76161389)

South Asians only

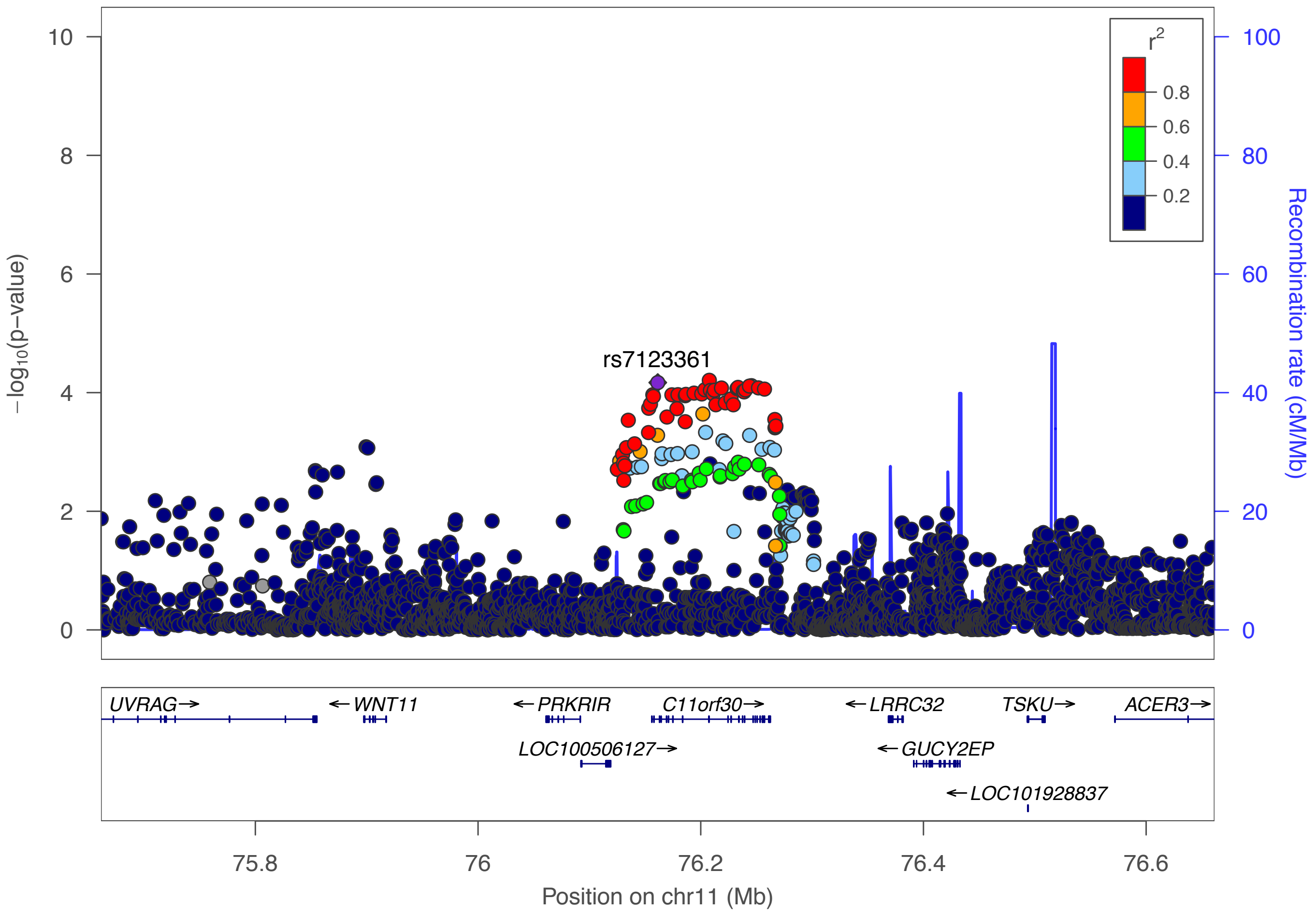

South Asians + Europeans

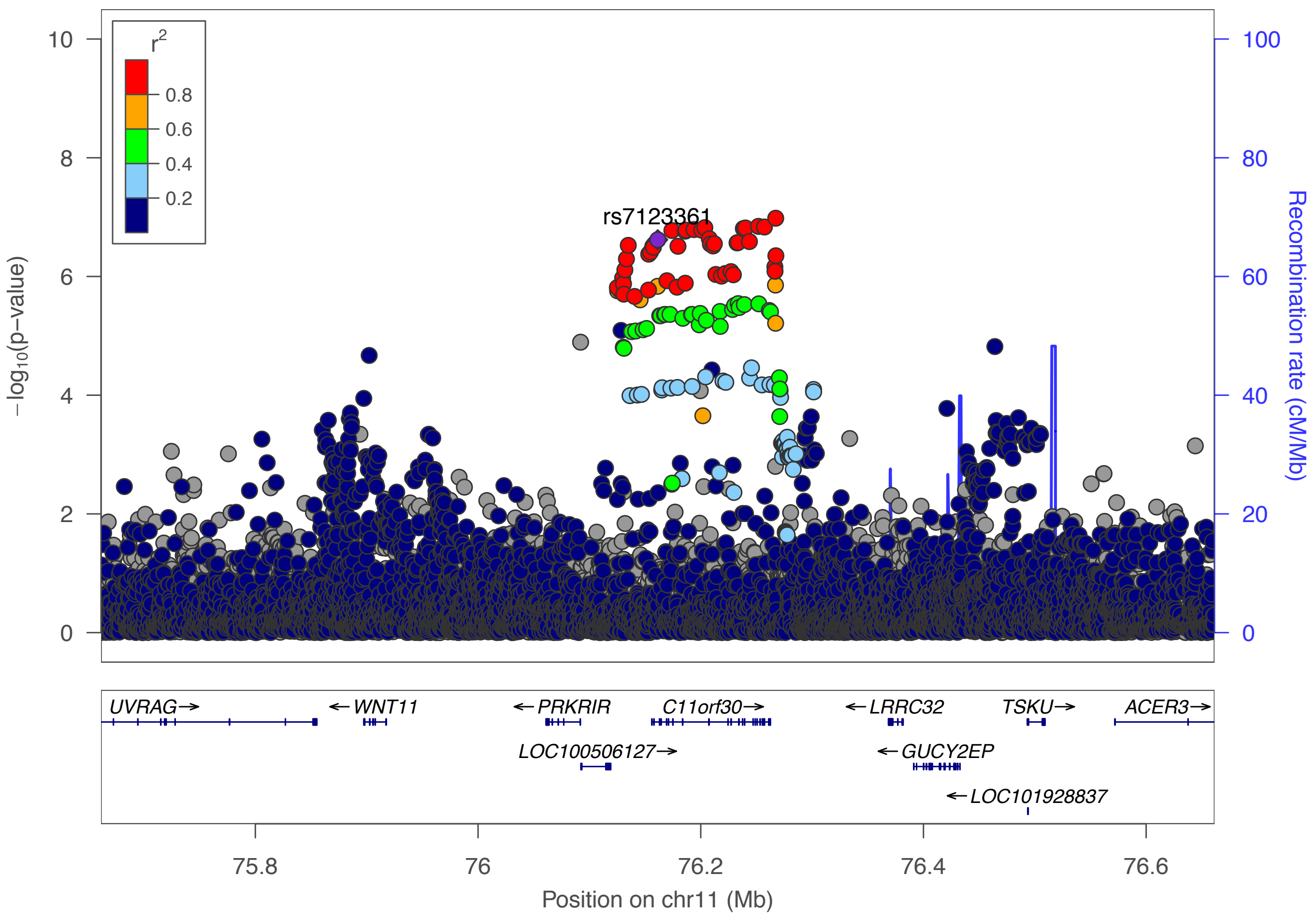

Supplementary Figure 39. Regional plots for rs7123361 showing association of SNPs with T2D for BMI adjusted model in South Asians only, and in South Asians and Europeans.

rs9568861 (chr13:54079446)

South Asians only

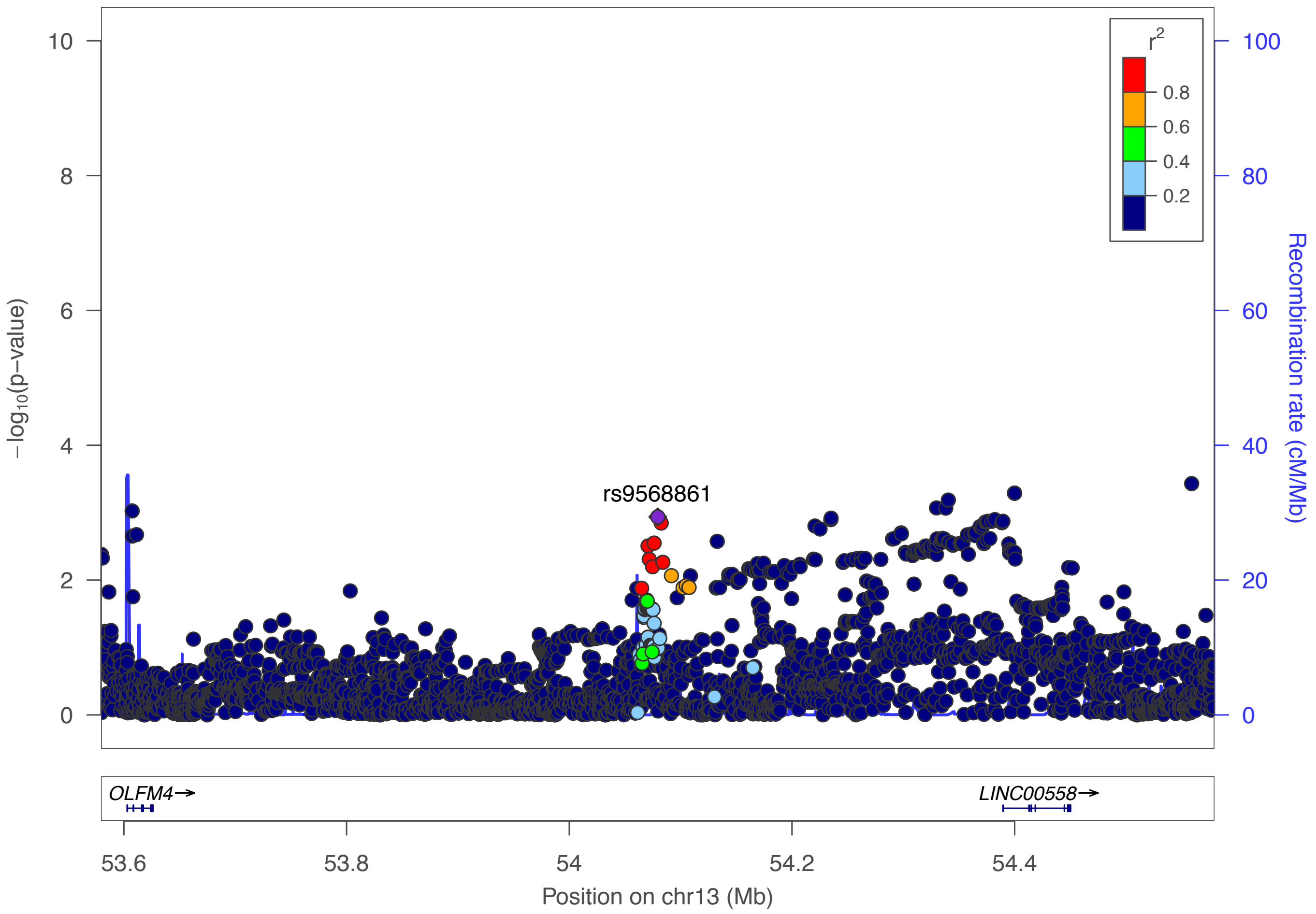

South Asians + Europeans

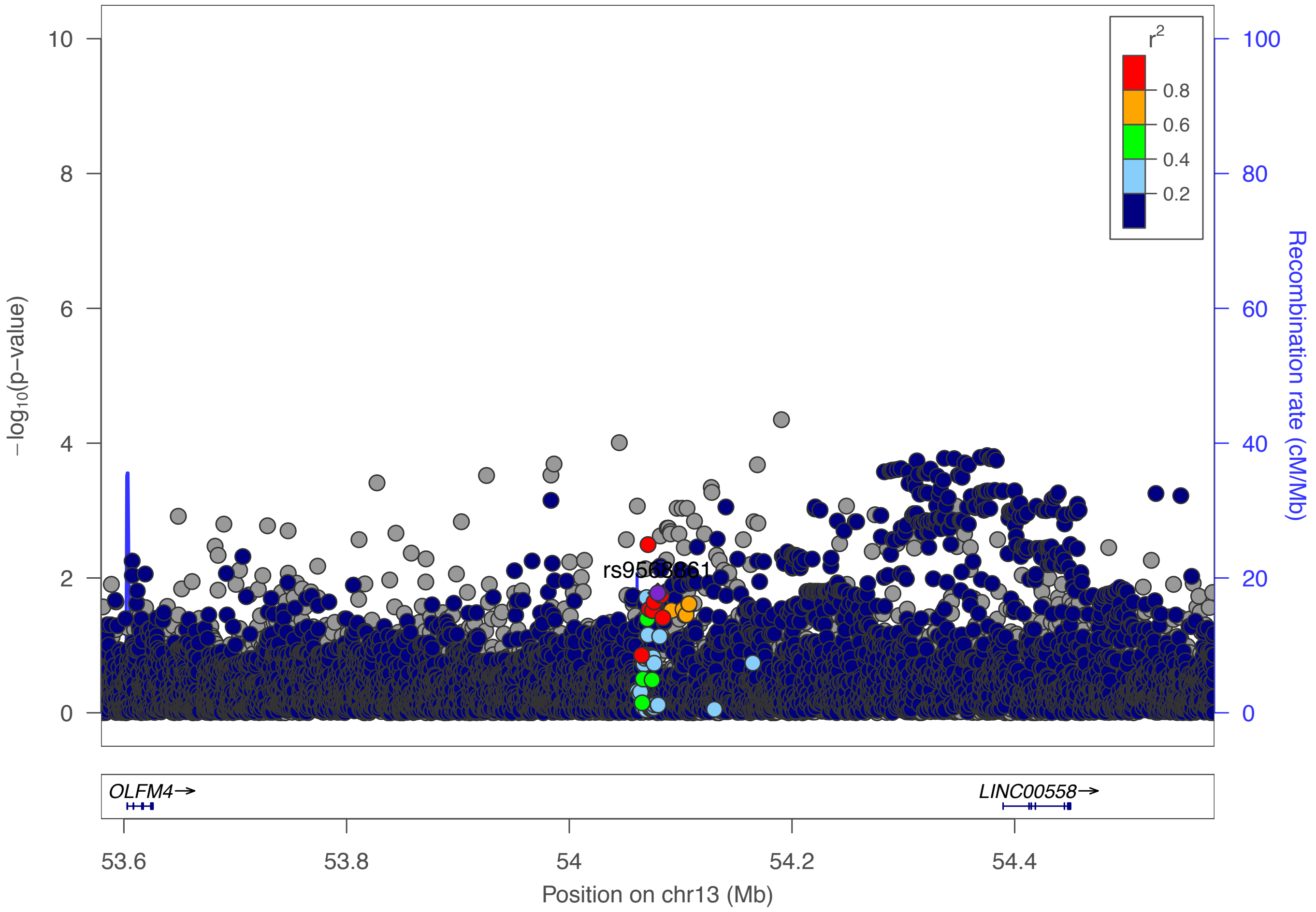

Supplementary Figure 40. Regional plots for rs9568861 showing association of SNPs with T2D for BMI adjusted model in South Asians only, and in South Asians and Europeans.

rs76141923 (chr13:81646630)

South Asians only

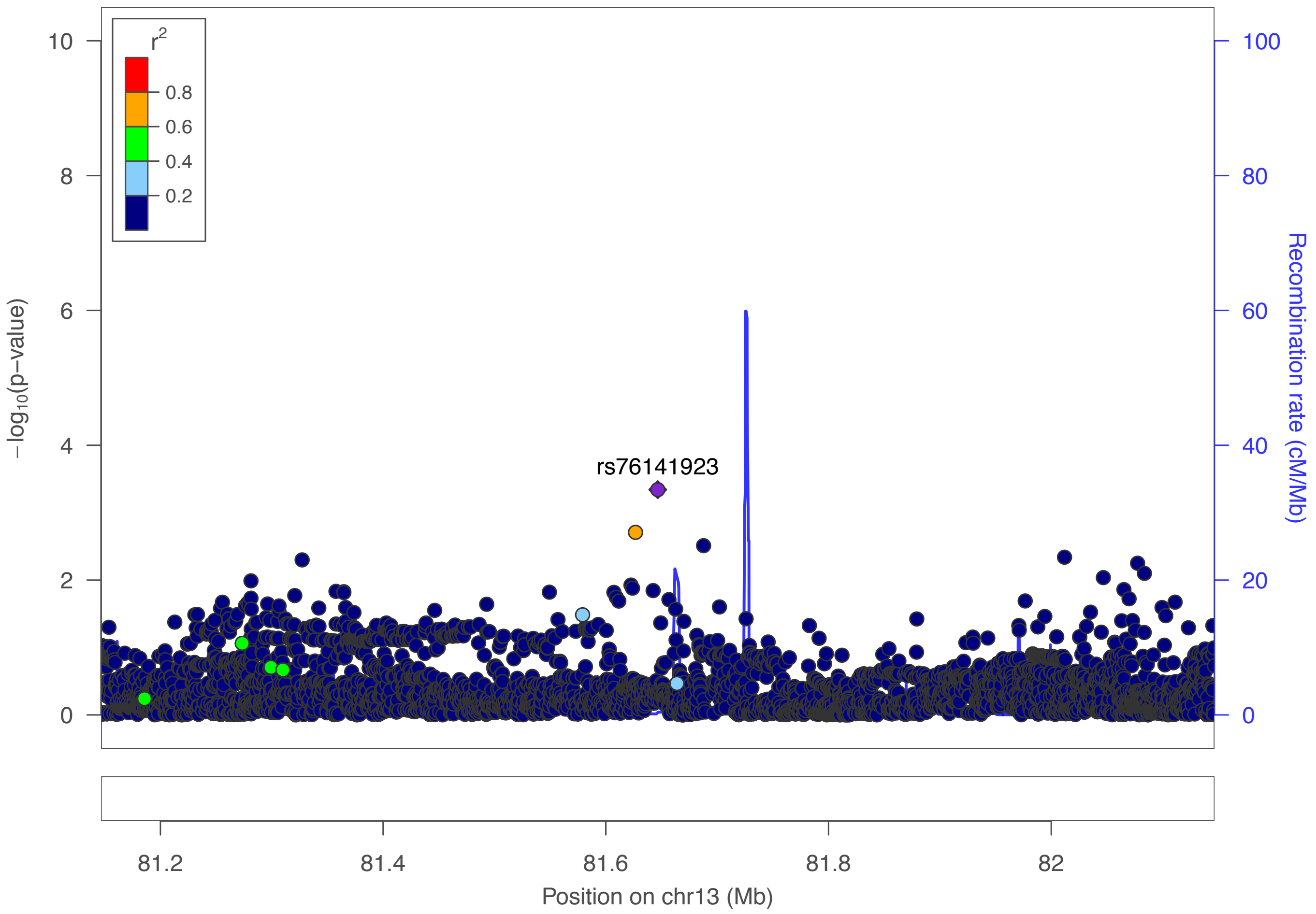

South Asians + Europeans

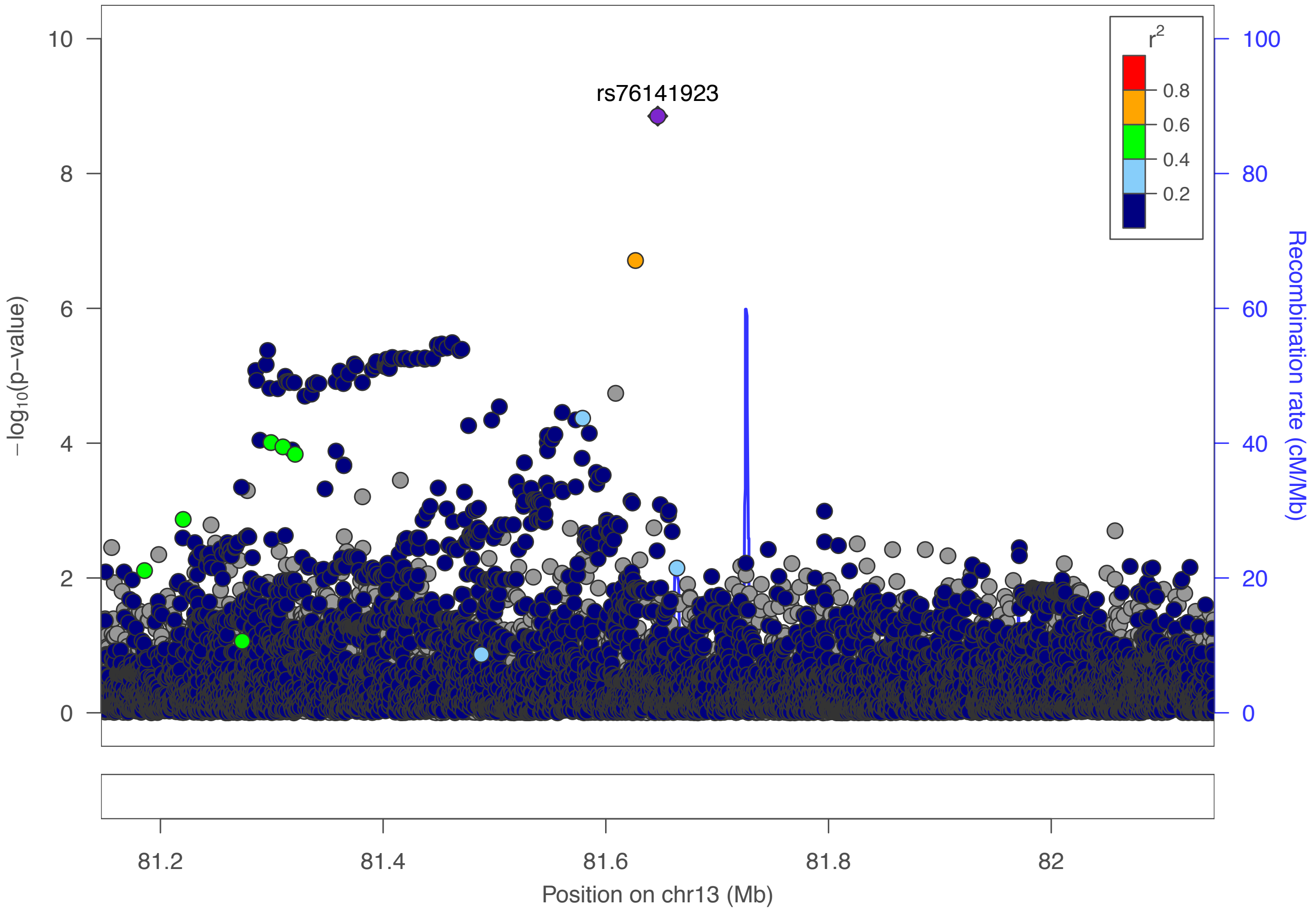

Supplementary Figure 41. Regional plots for rs76141923 showing association of SNPs with T2D for BMI adjusted model in South Asians only, and in South Asians and Europeans.

rs28790585 (chr15:39668870)

South Asians only

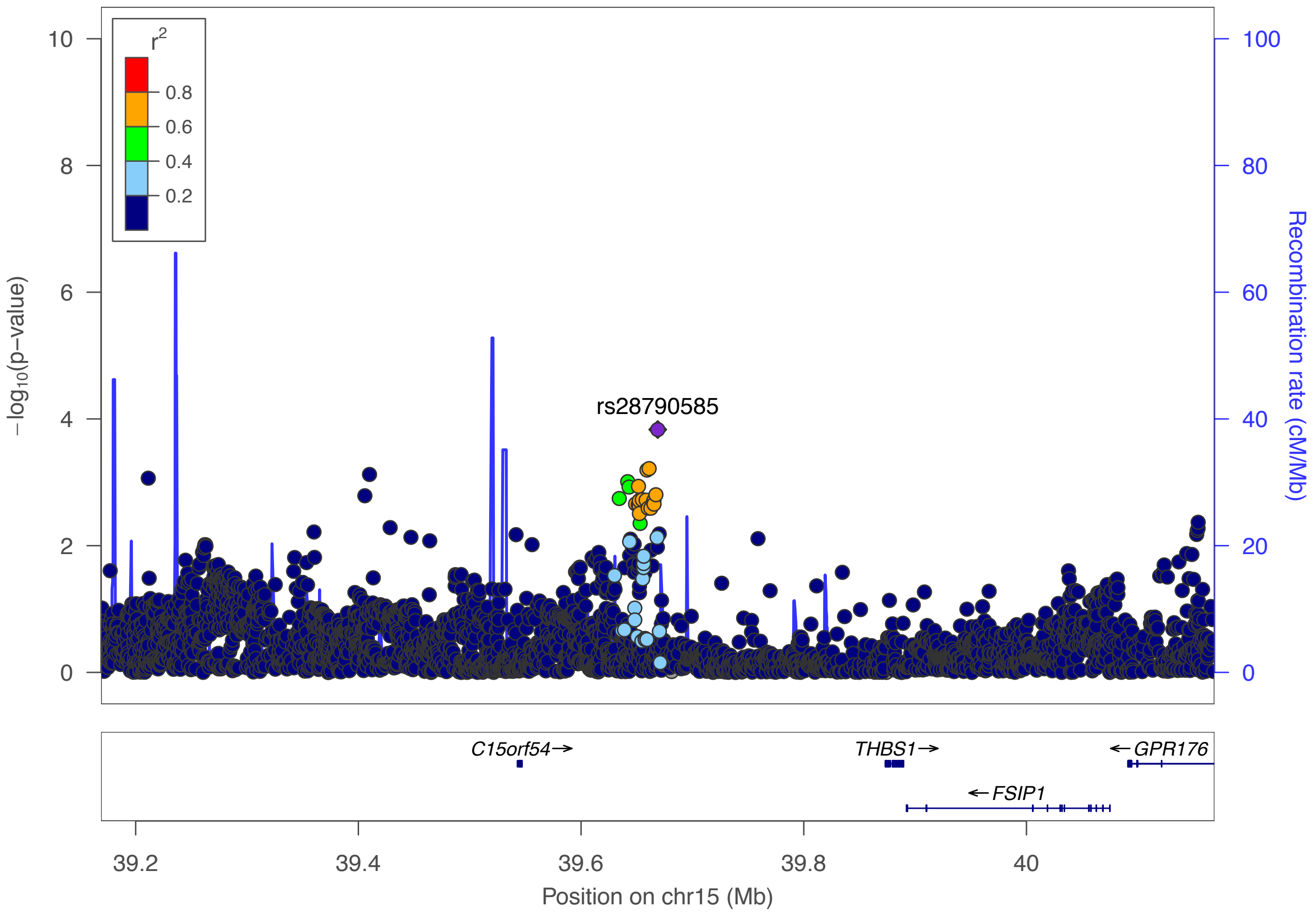

South Asians + Europeans

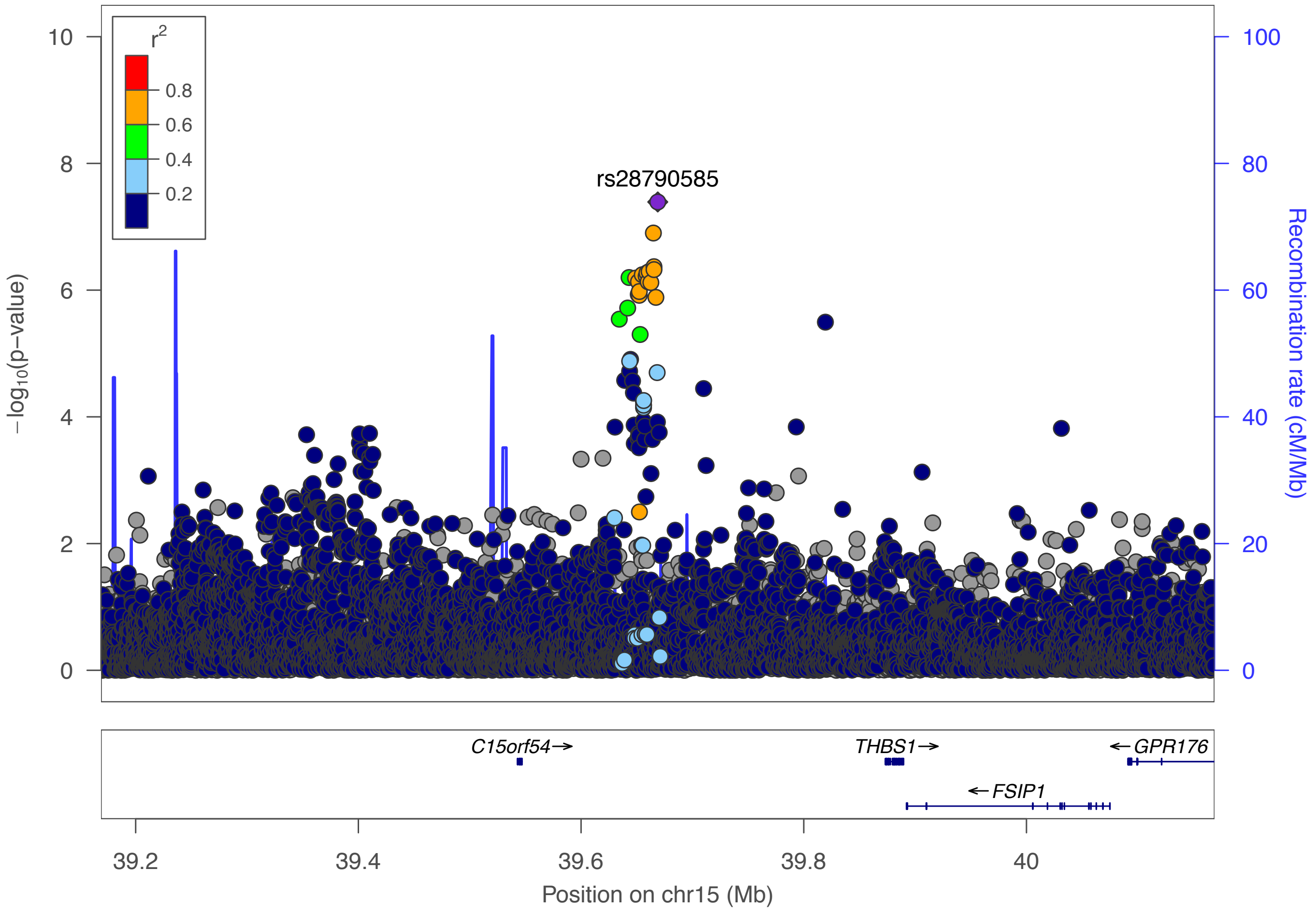

Supplementary Figure 42. Regional plots for rs28790585 showing association of SNPs with T2D for BMI adjusted model in South Asians only, and in South Asians and Europeans.

rs7261425 (chr20:20068635)

South Asians only

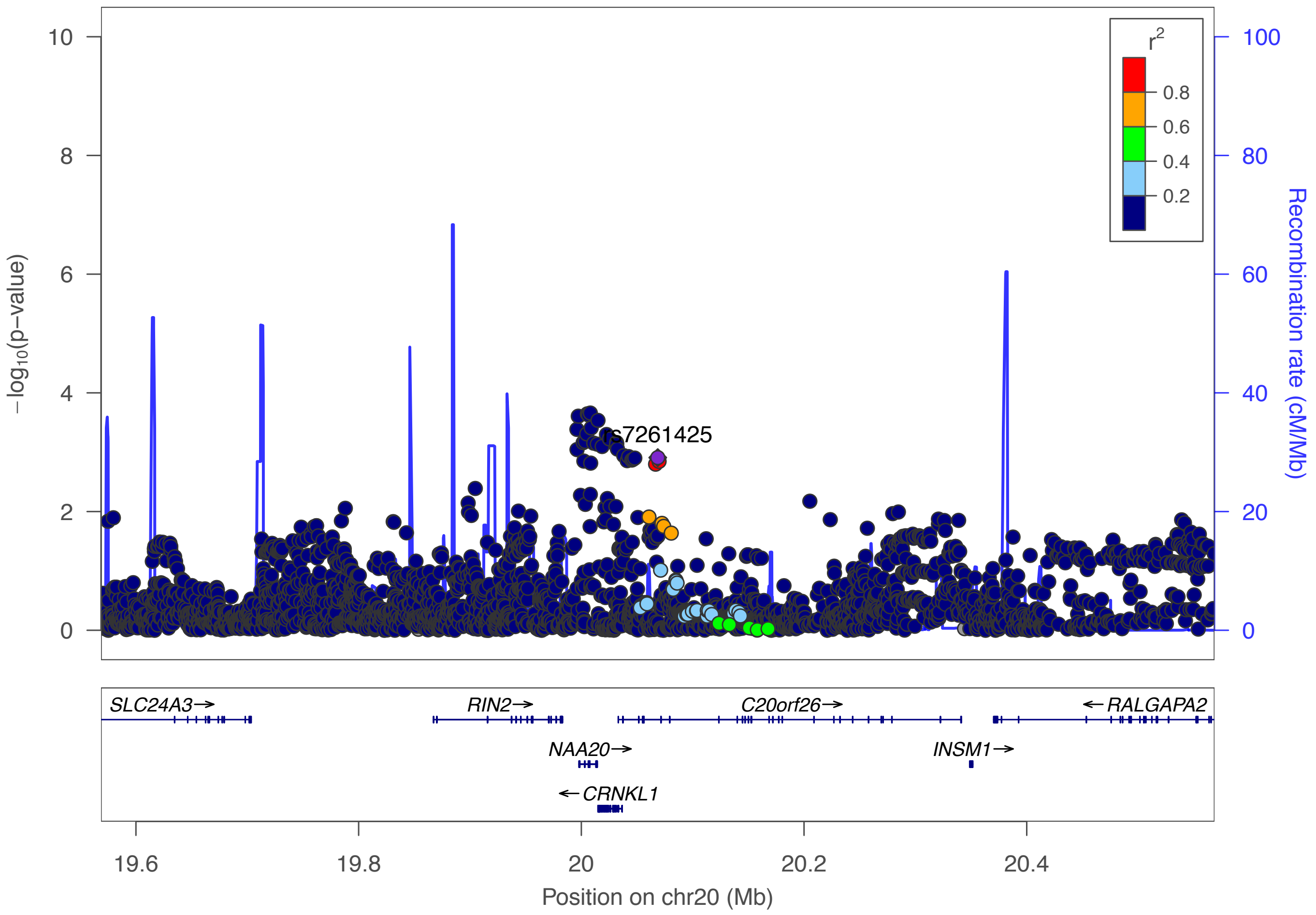

South Asians + Europeans

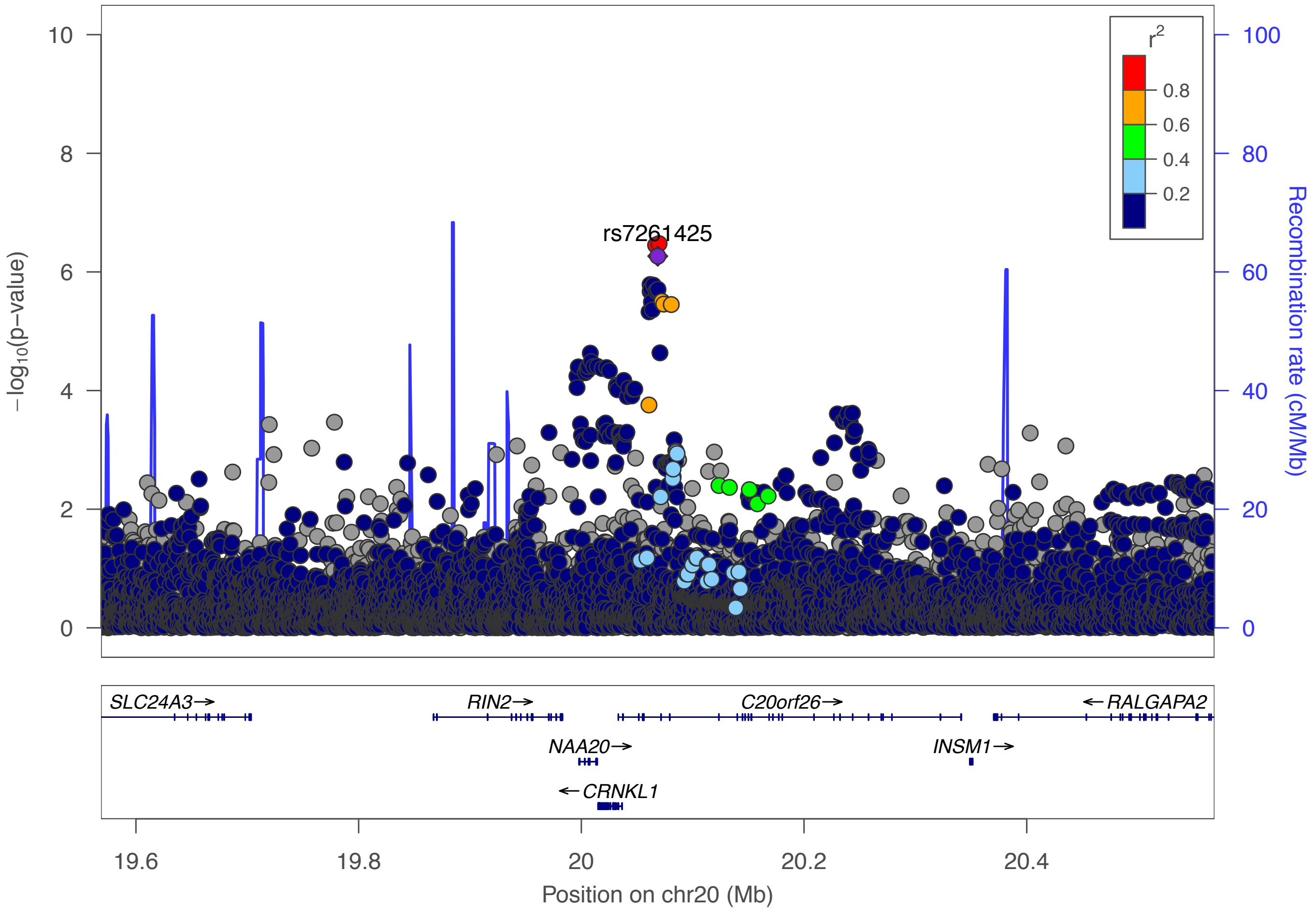

Supplementary Figure 43. Regional plots for rs7261425 showing association of SNPs with T2D for BMI adjusted model in South Asians only, and in South Asians and Europeans.

rs2065703 (chr20:31966698)

South Asians only

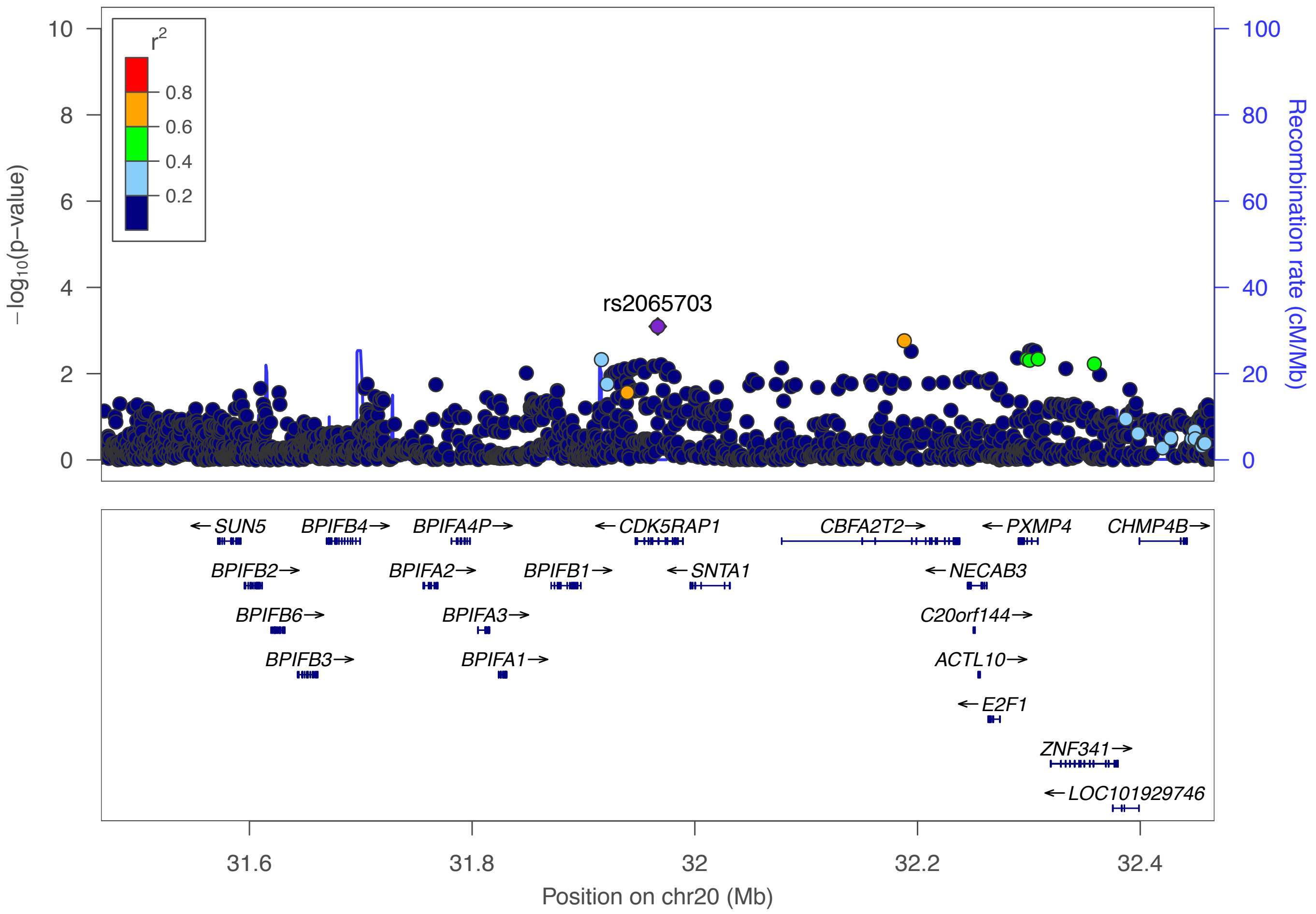

South Asians + Europeans

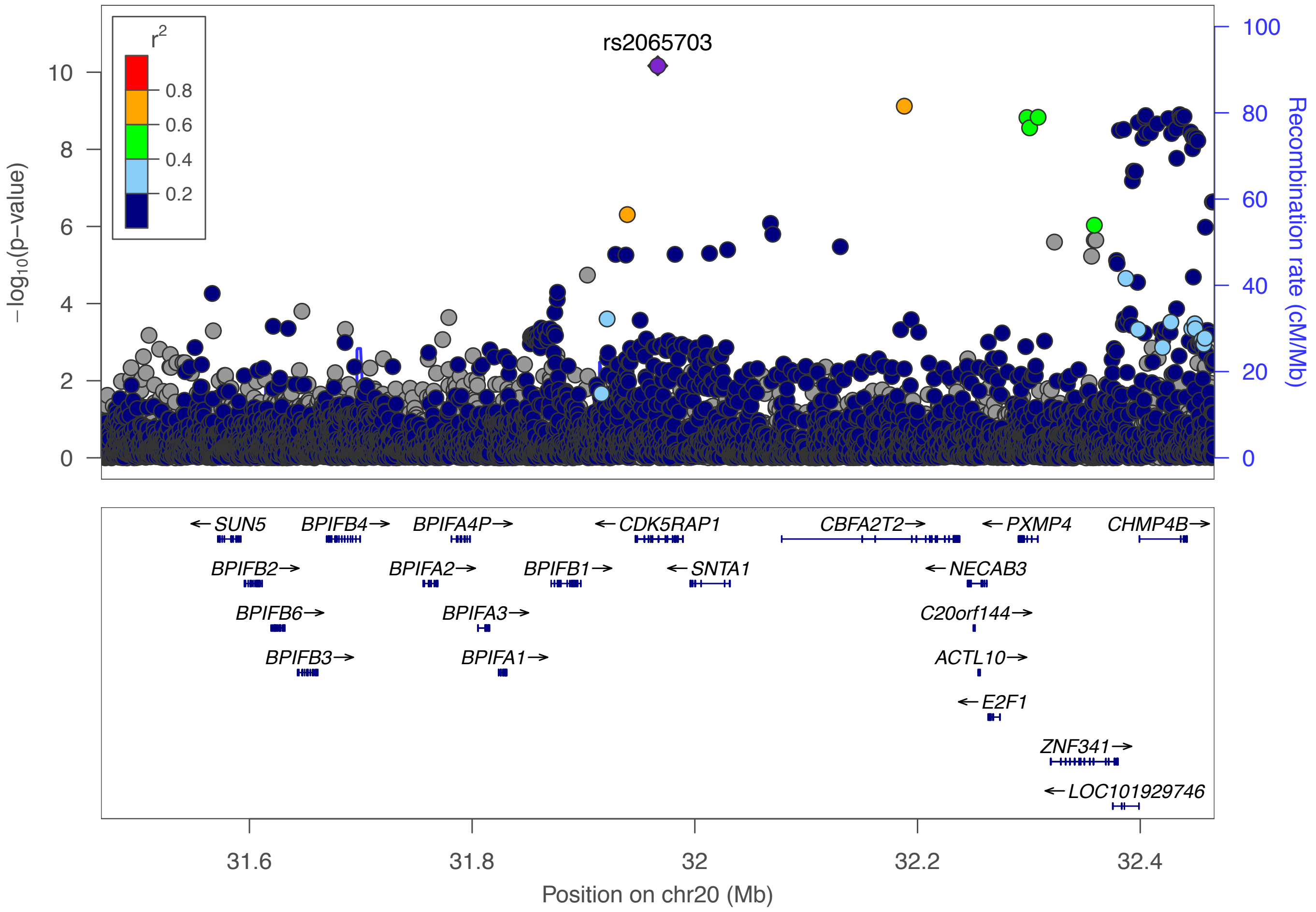

Supplementary Figure 44. Regional plots for rs2065703 showing association of SNPs with T2D for BMI adjusted model in South Asians only, and in South Asians and Europeans.

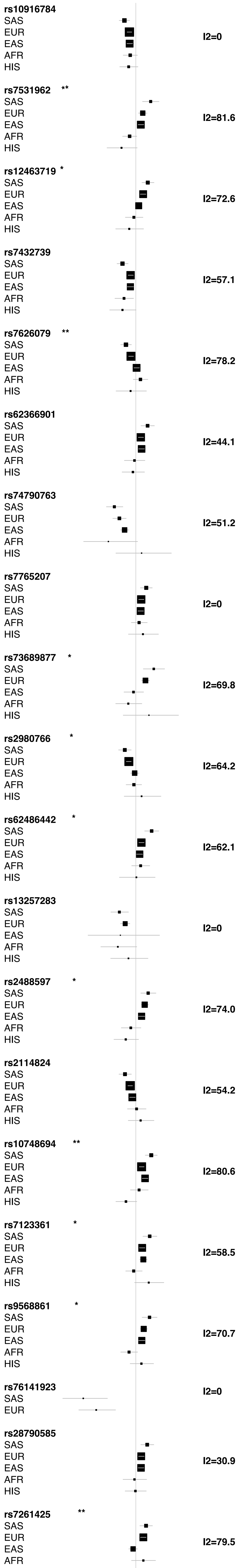

Supplementary Figure 45. Forest plots showing the associations with T2D for the 21 novel sentinel SNPs in South Asians, Europeans, East Asians, Africans and Hispanics in BMI unadjusted model. Size of square is proportional to sample size of the respective ethnic populations, with error bars indicative of 95% confidence intervals. Populations displaying significant heterogeneity in effect sizes relative to the South Asian population (P-het <0.05) are marked by asterisks.

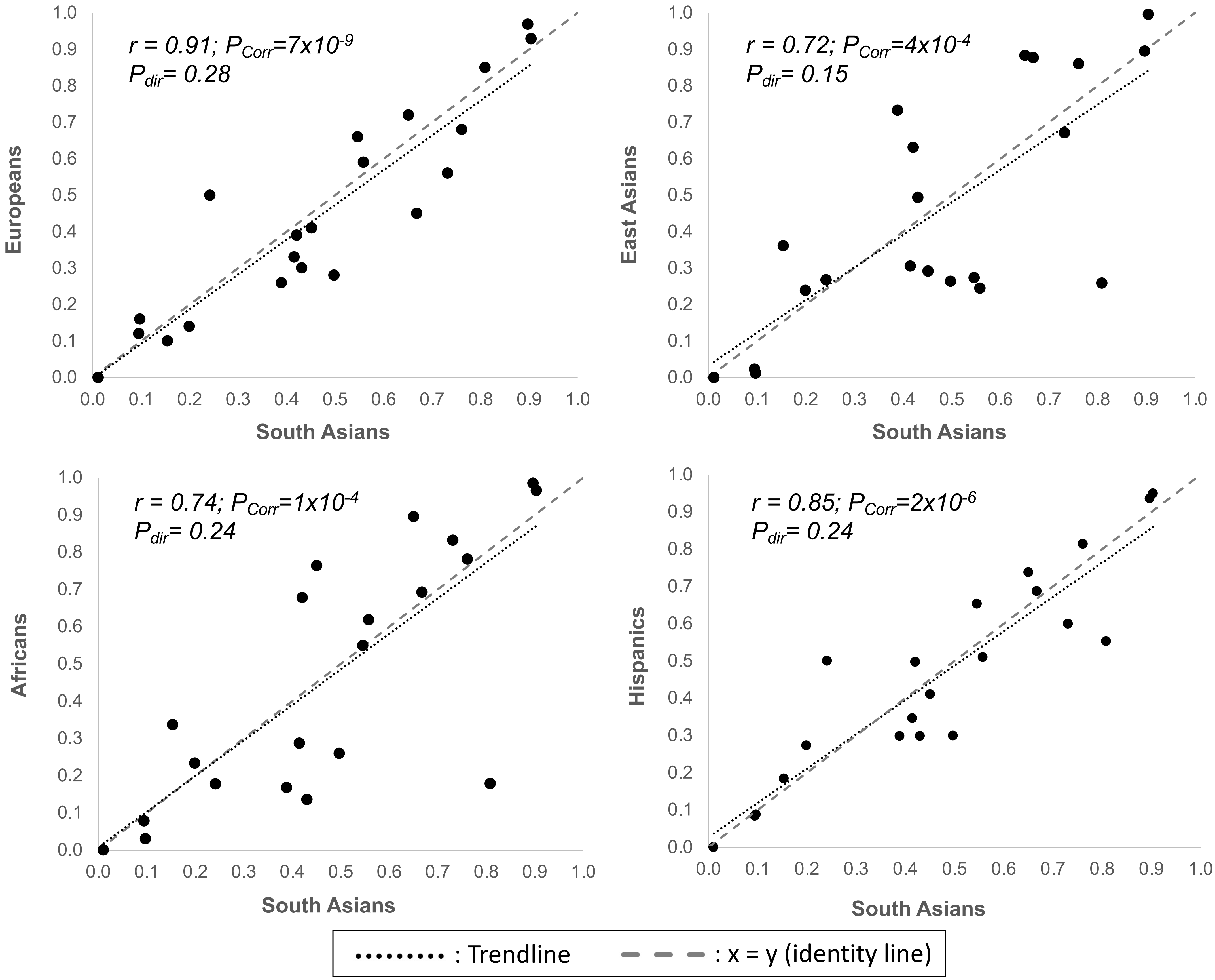

Supplementary Figure 46. Scatterplots showing effect allele frequencies (EAFs) for the 21 novel sentinel SNPs in South Asians, Europeans, East Asians, Africans and Hispanics. Pearson correlation coefficients and corresponding P-values (PCorr), as well as P-values for consistent in directionality of allele frequencies assessed by sign test (Pdir) were expressed.

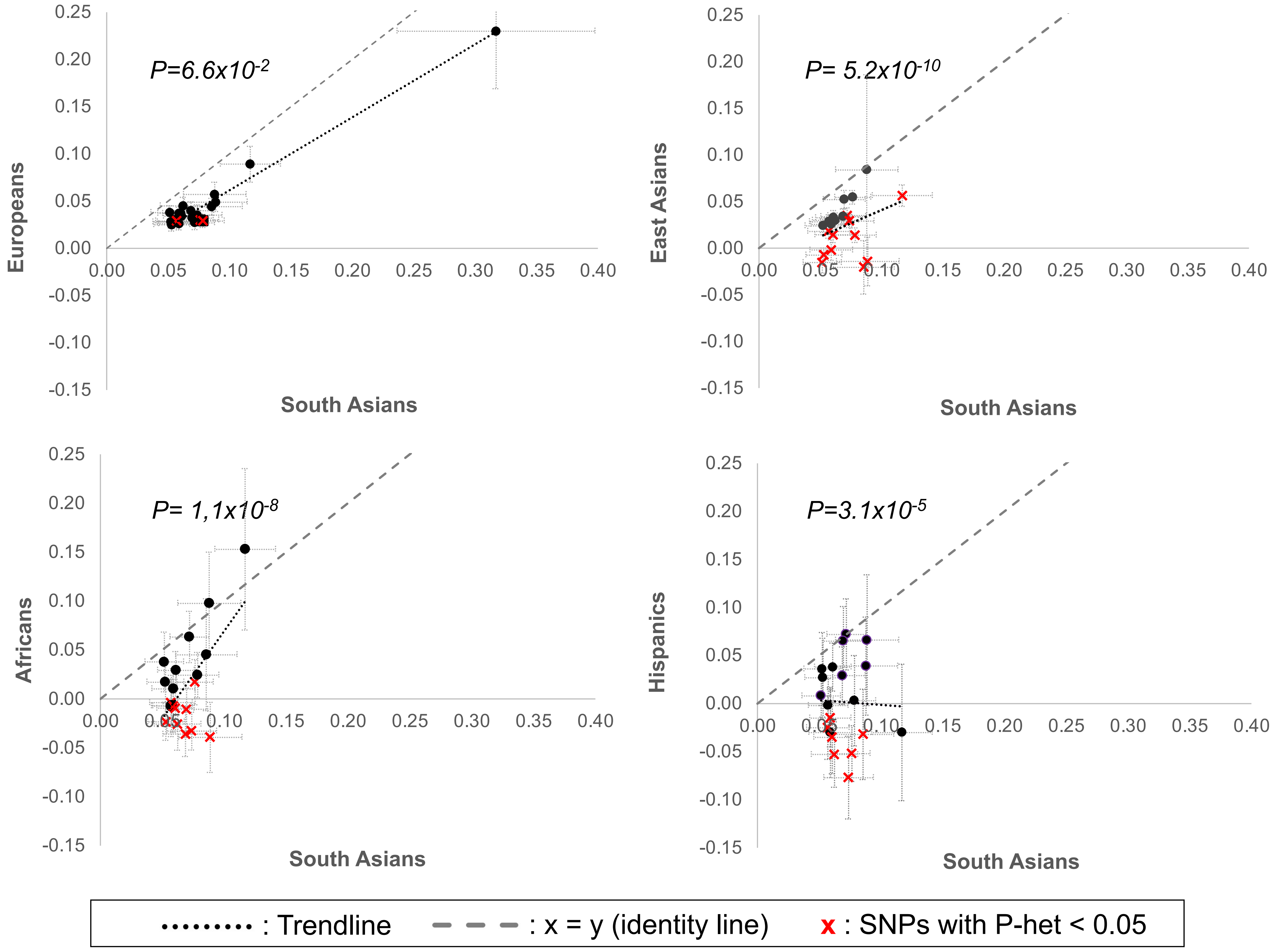

Supplementary Figure 47. Scatterplots showing effect sizes for the 21 novel sentinel SNPs in South Asians, Europeans, East Asians, Africans and Hispanics in BMI unadjusted model. Standard error (SE) for effect sizes were indicated by error bars, with SNPs displaying significant heterogeneity in effect sizes between the respective populations relative to South Asians represented by red crosses (P-het<0.05), with P-values for enrichment of SNPs with heterogeneous effect sizes indicated.

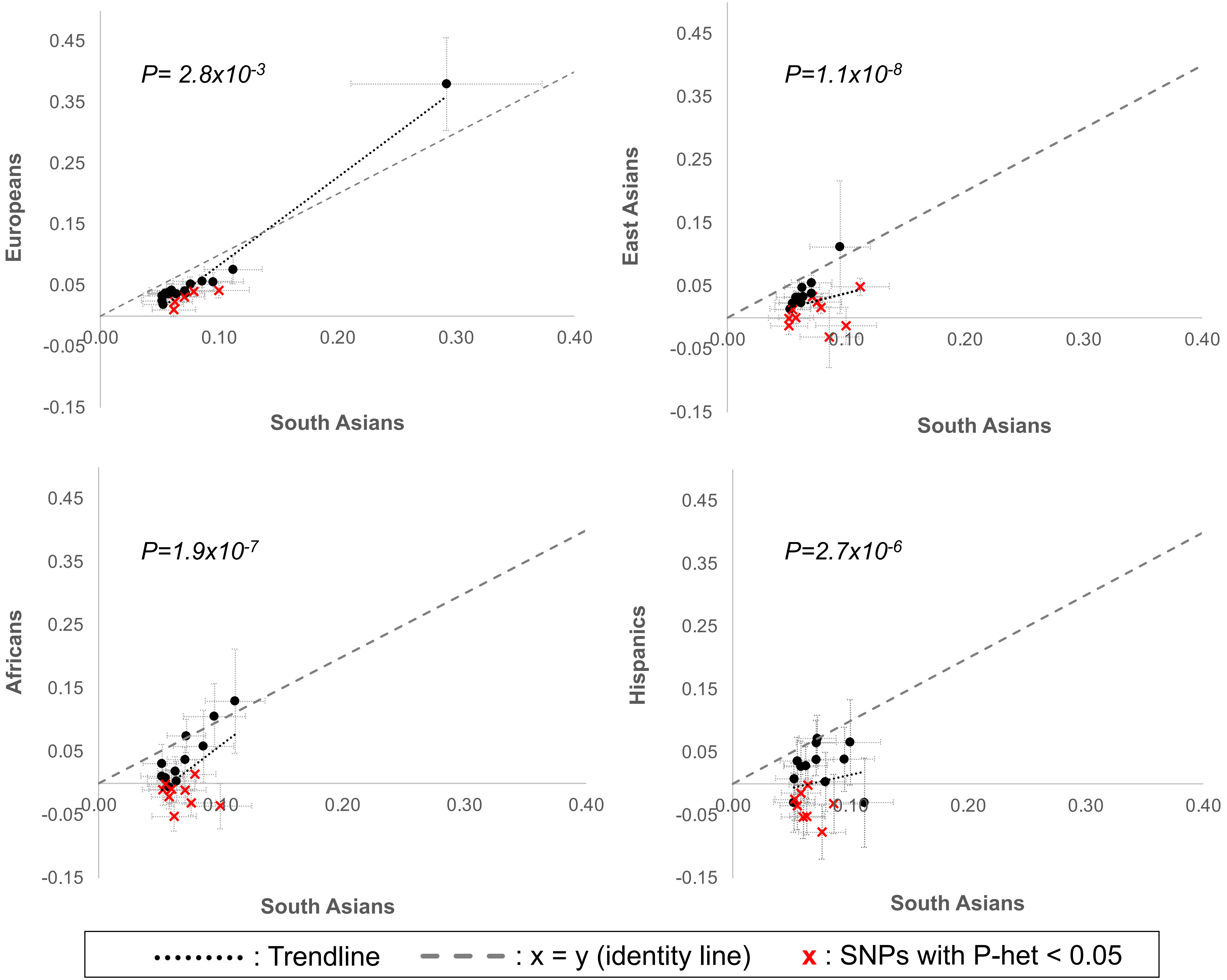

Supplementary Figure 48. Scatterplots showing effect sizes for the 21 novel sentinel SNPs in South Asians, Europeans, East Asians, Africans and Hispanics in BMI adjusted model. Standard error (SE) for effect sizes were indicated by error bars, with SNPs displaying significant heterogeneity in effect sizes between the respective populations relative to South Asians represented by red crosses (P-het<0.05), with P-values for enrichment of SNPs with heterogeneous effect sizes indicated.

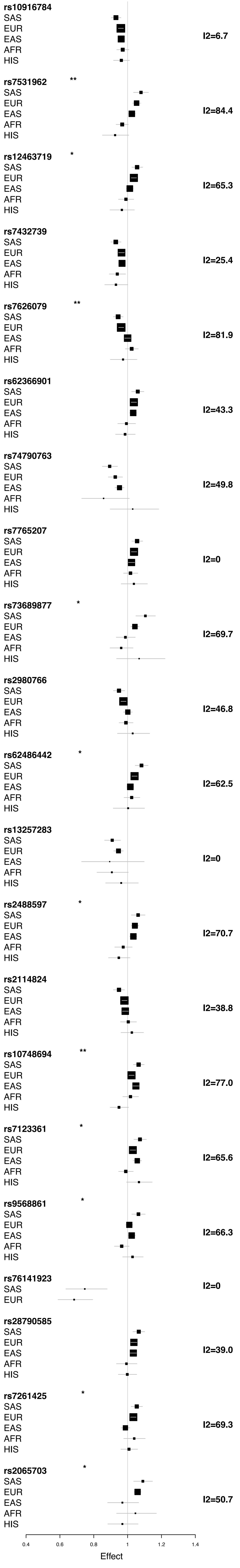

Supplementary Figure 49. Forest plots showing the associations with T2D for the 21 novel sentinel SNPs in South Asians, Europeans, East Asians, Africans and Hispanics in BMI adjusted model. Size of square is proportional to sample size of the respective ethnic populations, with error bars indicative of 95% confidence intervals. Populations displaying significant heterogeneity in effect sizes relative to the South Asian population (P-het <0.05) are marked by asterisks.

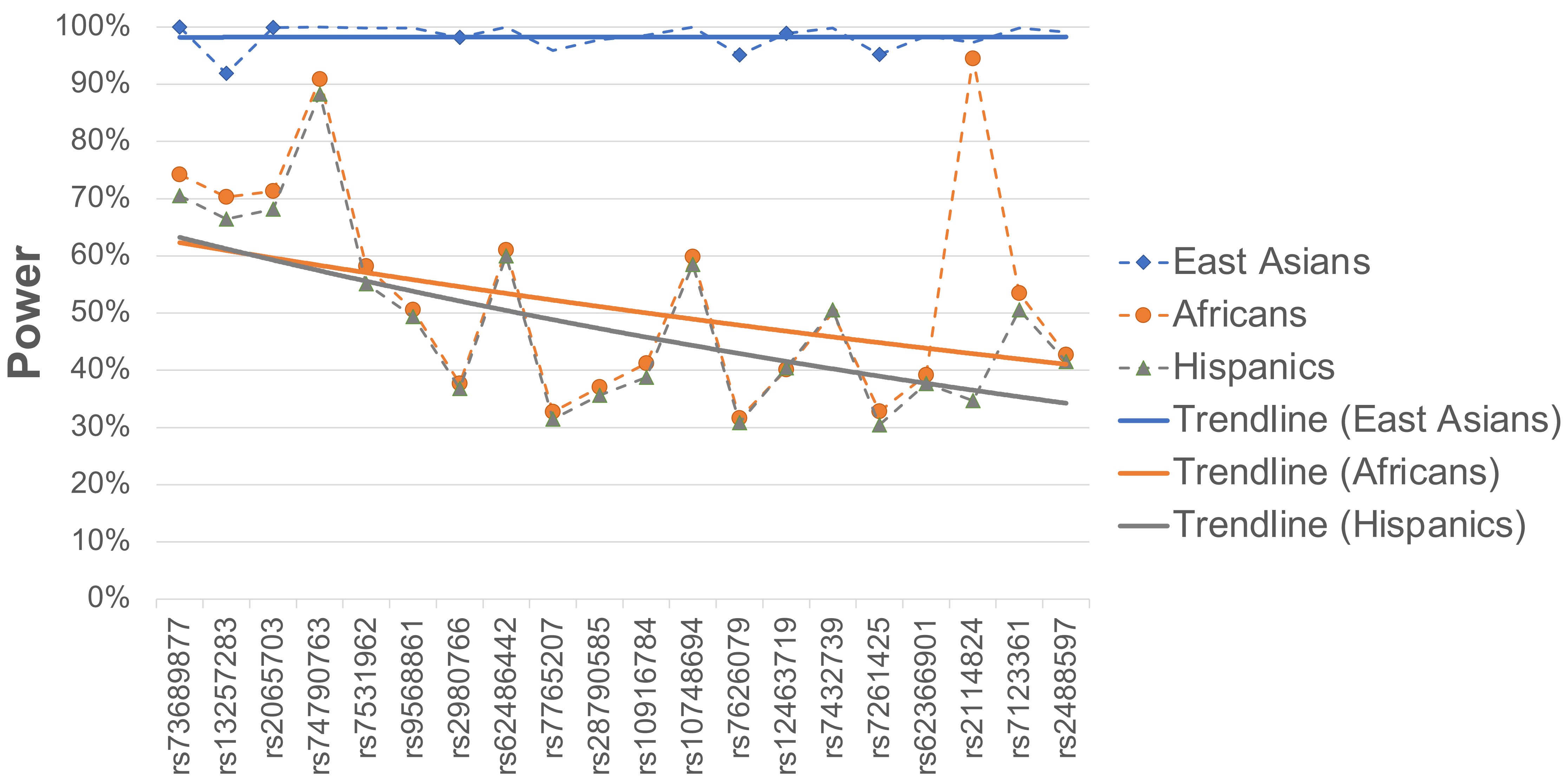

Supplementary Figure 50. Power to identify effect sizes as observed in South Asians at the 21 novel sentinel SNPs in East Asians, Africans and Hispanics based on BMI unadjusted model. The dashed lines indicate the statistical power achievable with the sample sizes available in East Asians (blue diamond), Africans (orange circle) and Hispanics (grey triangle) at P=0.05, while the solid lines represent the respective trendlines. SNPs that did not replicate in the respective populations were marked with diamonds. rs76141923 was not included in the plot as it was monomorphic in all three populations.

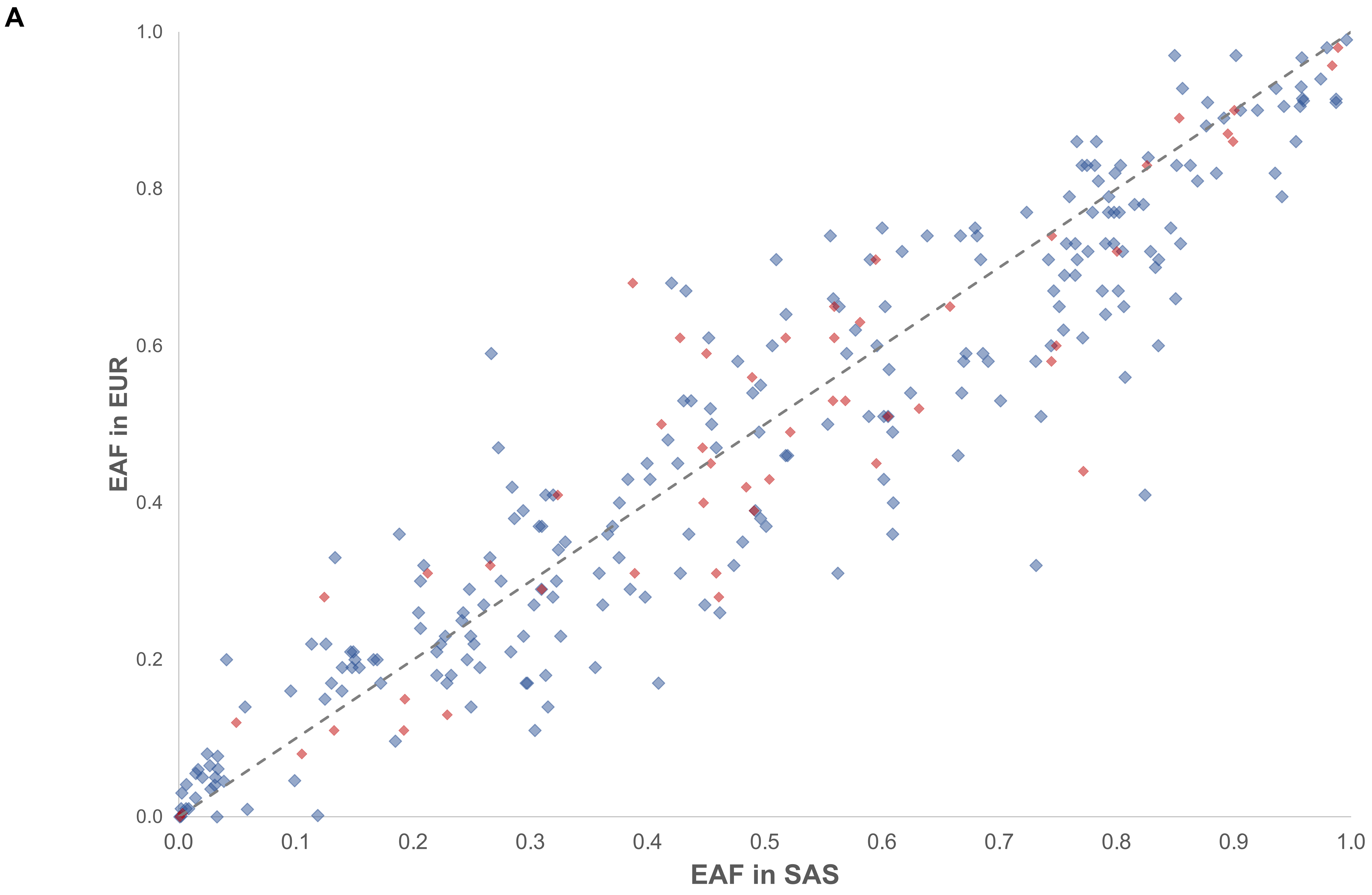

Supplementary Figure 51. Scatterplots showing effect allele frequencies (EAFs) for sentinel SNPs at known T2D loci in South Asian and European populations for BMI unadjusted model. P-values were indicated according to sign test, whereby 'success' was defined as SNP with higher EAF in South Asians compared to Europeans. Overall, we observed weak evidence for enrichment of known T2D variants with higher EAF in South Asian (P=2.4x10-2). SNPs displaying significant heterogeneity in effect sizes between the two populations were represented by red diamonds (P-het<0.05), while those with no significant heterogeneity detected were coloured in blue. SNPs with no effect sizes reported in either of the populations (due to quality or minor allele frequency filters) were indicated by grey diamonds, while those with EAF unavailable in either of the two populations were removed from the plot (n=12).

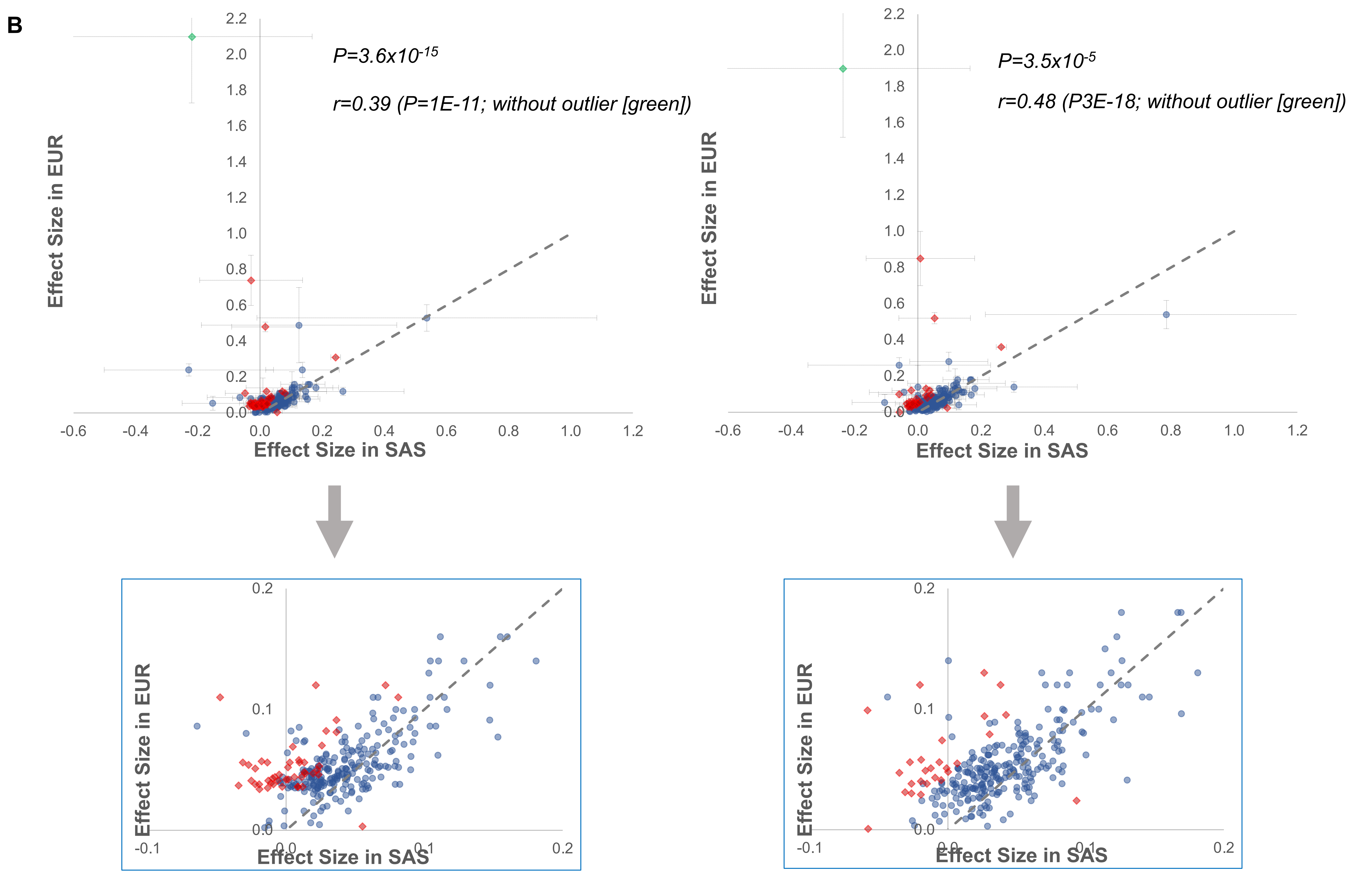

Supplementary Figure 52. Effect sizes for sentinel SNPs at known T2D loci in South Asian and European populations for a) BMI unadjusted and b) BMI adjusted model, with corresponding SEs indicated by error bars, and EAF distribution below each plot. P-values for enrichment of SNPs with heterogeneous effect sizes between the two populations were indicated. SNPs displaying significant heterogeneity in effect sizes between the two populations were represented by red diamonds (P-het<0.05), while those with no significant heterogeneity detected were coloured in blue.

a

PRS	Controls	Cases	P
Q1	1102	325	
Q2	957	473	2.39E-11
Q3	900	529	1.57E-19
Q4	737	692	4.51E-50

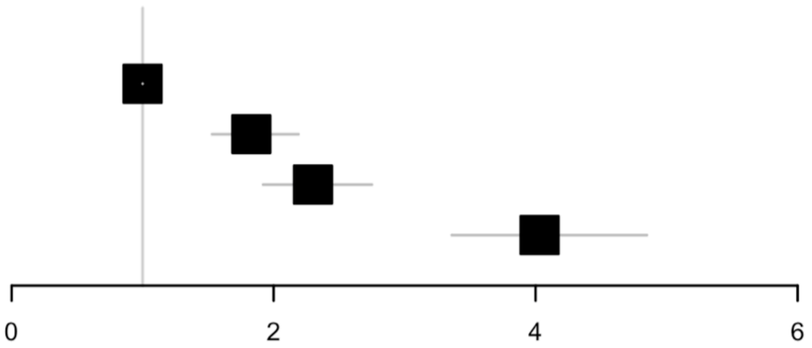

b

High PRS Definition	Reference Group	P
Top 20% of distribution	Remaining 80%	4.01E-33
Top 10% of distribution	Remaining 90%	2.90E-22
Top 5% of distribution	Remaining 95%	4.15E-19
Top 1% of distribution	Remaining 99%	4.86E-06

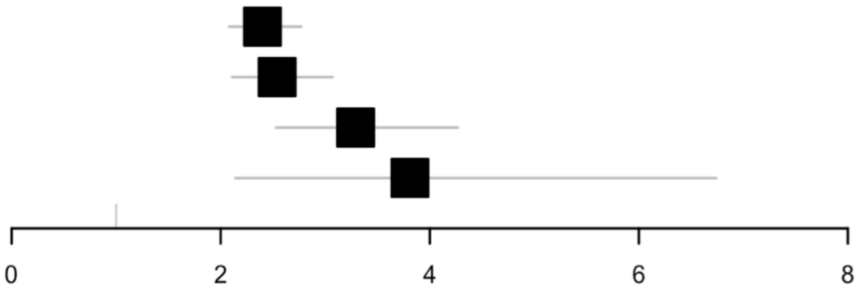

Supplementary Figure 53. Relative risk for T2D by a) quartile of polygenic risk score (PRS), compared to the lowest quartile (Q1) and b) comparing the individuals with high PRS compared to the remainder of the population. Error bars are indicative of 95% confidence intervals.

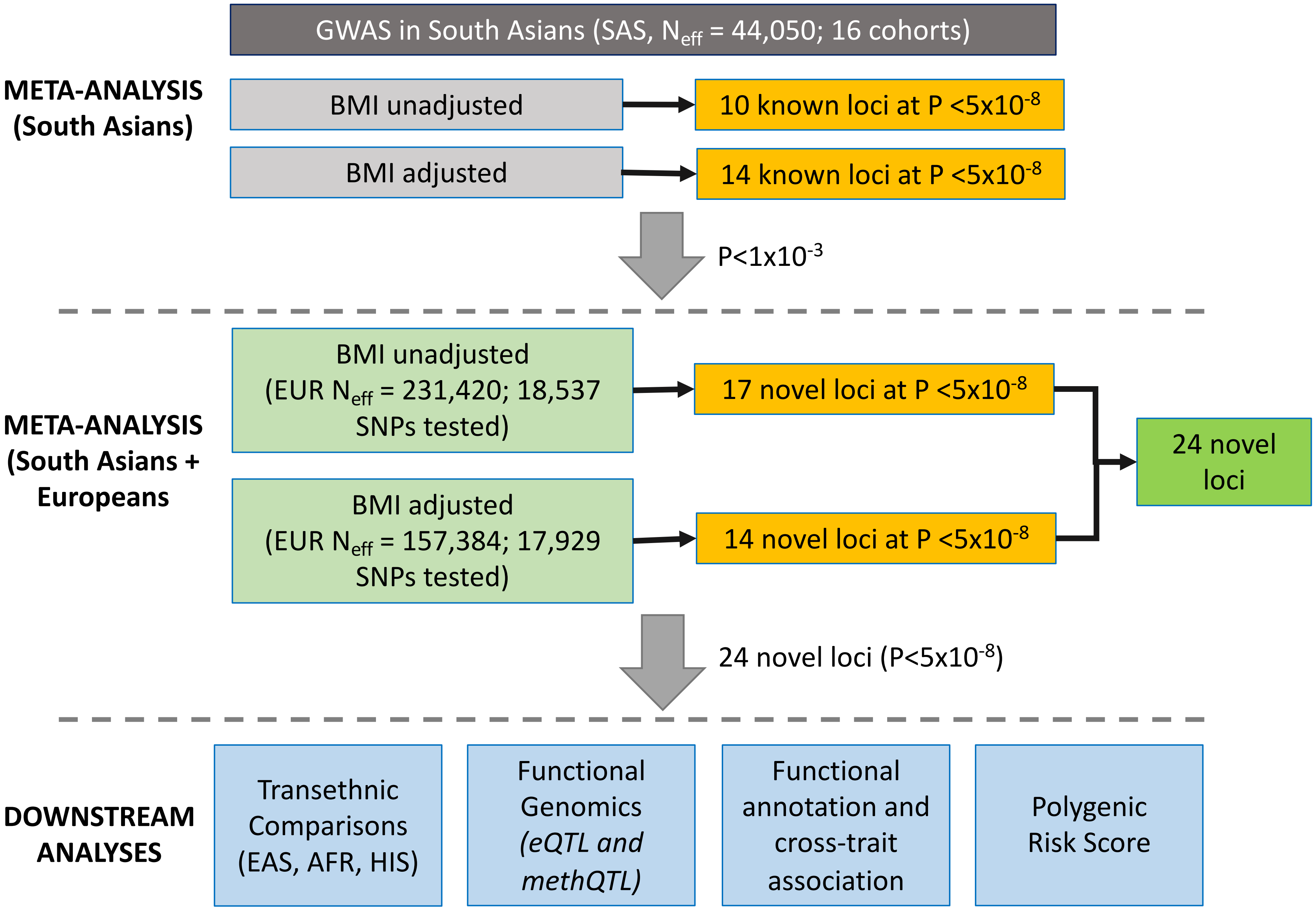

Supplementary Figure 54. Study design.
