# Supplementary material for: Identification of genetic effects underlying type 2 diabetes in South Asian and European populations
Source: Commun Biol. 2022 Apr 7;5:329. doi: 10.1038/s42003-022-03248-5 (PMC8991226; doi:10.1038/s42003-022-03248-5)
Supplement: Supplementary file 2 — Description of Additional Supplementary Files [file 42003_2022_3248_MOESM2_ESM.pdf]

Description of Additional Supplementary Files

Supplementary Data 1. Participating South Asian cohorts and sample characteristics.

Supplementary Data 2. Cohort-level quality control, pre-phasing, imputation and association analysis.

Supplementary Data 3. Number of SNPs reaching significance at different P-value thresholds in genome-wide meta-analyses of South Asians only.

Supplementary Data 4. Association results for sentinel SNPs of 14 known loci (P-values < $5x10^{-8}$) identified in BMI unadjusted and adjusted models from genome-wide meta-analysis of South Asians only.

Supplementary Data 5. Association results for 218 SNPs across 14 novel loci reaching genome-wide significance ($P<5x10^{-8}$) in combined analysis of South Asians and Europeans for BMI unadjusted model.

Supplementary Data 6. Association results for 185 SNPs across 11 novel loci reaching genome-wide significance (P<5E-8) in combined analysis of South Asians and Europeans for BMI adjusted model.

Supplementary Data 7. Results for conditional analysis for novel sentinel SNPs, adjusted for known SNPs in the same loci (up to +/-1Mb). IA610 was used as the LD reference population.

Supplementary Data 8. Effect of BMI adjustment on association of novel loci with T2D in South Asians.

Supplementary Data 9. Gender-specific analyses of novel loci in South Asians for BMI unadjusted and adjusted models.

Supplementary Data 10. Transethnic comparison of association with T2D for 21 novel sentinel SNPs in genome-wide meta-analysis for BMI unadjusted model.

Supplementary Data 11. Transethnic comparison of association with T2D for 21 novel sentinel SNPs in genome-wide meta-analysis for BMI adjusted model.

Supplementary Data 12. Comparison of association with T2D for known T2D loci in South Asians and Europeans for BMI unadjusted model. SNPs with no effect sizes reported in either of the populations (due to quality or minor allele frequency filters) were indicated by NAs. SNPs with unavailable allele frequencies in either of the two populations were removed from the table (n=12).

Supplementary Data 13. Comparison of association with T2D for known T2D loci in South Asians and Europeans for BMI adjusted model. SNPs with no effect sizes reported in either of the populations (due to quality or minor allele frequency filters) were indicated by NAs. SNPs with unavailable allele frequencies in either of the two populations were removed from the table (n=12).

Supplementary Data 14. Association results in South Asians and Europeans for 17,944 SNPs reaching P<1E-3 for BMI unadjusted model in South Asians only.

Supplementary Data 15. Association results in South Asians and Europeans for 17,215 SNPs reaching P<1E-3 for BMI adjusted model in South Asians only.

Supplementary Data 16. Significant expression quantitative trait loci (eQTLs) at the 21 loci via eQTLGen, with replication results in LOLIPOP (n=693).

Supplementary Data 17. Significant cis expression quantitative trait loci (eQTLs) in pancreatic islets.

Supplementary Data 18. Colocalization analysis between expression (eQTL) and T2D GWAS signals for BMI unadjusted model.

Supplementary Data 19. Colocalization analysis between expression (eQTL) and T2D GWAS signals for BMI adjusted model.

Supplementary Data 20. Significant cis methylation quantitative trait loci (methQTLs) with the 21 novel sentinel SNPs in discovery analysis without pruning.

Supplementary Data 21. Replication testing results for pruned (sentinel) cis methylation quantitative trait loci (methQTLs).

Supplementary Data 22. Colocalization results between expression (eQTL) and methylation (meQTL) signals.

Supplementary Data 23. Functional annotation for the 21 novel sentinel SNPs and their proxies (r2>0.8).

Supplementary Data 24. Cross-trait association lookup for the 21 novel sentinel SNPs and proxies (r2>0.8) with PhenoScanner.

Supplementary Data 25. Performance indices of polygenic risk score (PRS) for T2D in validation based on South Asian summary statistics.

Supplementary Data 26. Performance indices of polygenic risk score (PRS) for T2D in models based on South Asian, European and South Asian-European combined summary statistics.
