# Supplementary material for: Identification of genetic effects underlying type 2 diabetes in South Asian and European populations
Source: Commun Biol. 2022 Apr 7;5:329. doi: 10.1038/s42003-022-03248-5 (PMC8991226; doi:10.1038/s42003-022-03248-5)
Supplement: Supplementary file 4 — Reporting Summary [file 42003_2022_3248_MOESM4_ESM.pdf]

Corresponding author(s): John Chambers

Last updated by author(s): Dec 29, 2021

Reporting Summary

Nature Research wishes to improve the reproducibility of the work that we publish. This form provides structure for consistency and transparency in reporting. For further information on Nature Research policies, see our Editorial Policies and the Editorial Policy Checklist.

Statistics

For all statistical analyses, confirm that the following items are present in the figure legend, table legend, main text, or Methods section.

n/a	Confirmed	
☐	☒	The exact sample size (*n*) for each experimental group/condition, given as a discrete number and unit of measurement
☐	☒	A statement on whether measurements were taken from distinct samples or whether the same sample was measured repeatedly
☐	☒	The statistical test(s) used AND whether they are one- or two-sided<br>*Only common tests should be described solely by name; describe more complex techniques in the Methods section.*
☐	☒	A description of all covariates tested
☐	☒	A description of any assumptions or corrections, such as tests of normality and adjustment for multiple comparisons
☐	☒	A full description of the statistical parameters including central tendency (e.g. means) or other basic estimates (e.g. regression coefficient) AND variation (e.g. standard deviation) or associated estimates of uncertainty (e.g. confidence intervals)
☐	☒	For null hypothesis testing, the test statistic (e.g. *F*, *t*, *r*) with confidence intervals, effect sizes, degrees of freedom and *P* value noted<br>*Give P values as exact values whenever suitable.*
☒	☐	For Bayesian analysis, information on the choice of priors and Markov chain Monte Carlo settings
☒	☐	For hierarchical and complex designs, identification of the appropriate level for tests and full reporting of outcomes
☐	☒	Estimates of effect sizes (e.g. Cohen's *d*, Pearson's *r*), indicating how they were calculated

Our web collection on statistics for biologists contains articles on many of the points above.

Software and code

Policy information about availability of computer code

Data collection	Cohort-level (n = 16 cohorts) quality control, pre-phasing, imputation and association analysis details, including software/tools and versions are available in ST2.
Data analysis	METAL (generic-metal-2011-03-25)<br>GCTA (version 1.91.0 beta)<br>GWAMA (2.2.2)<br>Matrix eQTL<br>coloc (2.3-1)<br>HaploRegv4.1<br>haploR package in R (version 3.6.0)<br>popcorn (https://github.com/brielin/Popcorn)<br>LDpred (v1.0.11)

For manuscripts utilizing custom algorithms or software that are central to the research but not yet described in published literature, software must be made available to editors and reviewers. We strongly encourage code deposition in a community repository (e.g. GitHub). See the Nature Research guidelines for submitting code & software for further information.

Data

Policy information about availability of data

All manuscripts must include a data availability statement. This statement should provide the following information, where applicable:

- Accession codes, unique identifiers, or web links for publicly available datasets
- A list of figures that have associated raw data
- A description of any restrictions on data availability

Summary statistics data is available via GWAS catalogue (GCP000269).

Field-specific reporting

Please select the one below that is the best fit for your research. If you are not sure, read the appropriate sections before making your selection.

☒ Life sciences ☐ Behavioural & social sciences ☐ Ecological, evolutionary & environmental sciences

For a reference copy of the document with all sections, see nature.com/documents/nr-reporting-summary-flat.pdf

Life sciences study design

All studies must disclose on these points even when the disclosure is negative.

Sample size	Sixteen South Asian cohorts with 50,533 participants were included in the meta-analysis (16,677 cases and 33,856 non-prediabetic normal controls). This sample size allows us to detect significant associations at P-value<5x10-8 for SNPs with MAF≥0.05 and odds ratios (OR) ≥1.21, or MAF ≥0.20 and OR ≥1.11 at 80% power, taking an additive disease model.
Data exclusions	Pre-diabetic participants who are neither T2D cases nor normoglycaemic controls were removed from 12 out of 16 cohorts analysed in this study.<br>Standard QC for samples included removing samples with low call rate (e.g. <95%), extreme heterozygosity, mismatch of sex, and those of duplicates, relatedness, and population outliers. QC for variants included removing variants with low call rate (<95%), Hardy-Weinberg equilibrium P-value<1x10-6, or minor allele frequency (MAF) <1%.<br>Association summary statistics were filtered for QC before meta-analysis. Criteria for inclusion of a variant in association meta-analysis included imputation info≥0.4 for IMPUTE2 and 0.3 for MACH/minimac,64-66 minor allele count (MAC)≥6, P-values for Hardy-Weinberg equilibrium≥1x10-6, standard error (SE)>0 and <10, and P-values >0 and ≤1.
Replication	For our methQTL analysis, we measured DNA methylation in 1,841 South Asians using peripheral blood sample collected at baseline from the London Life Sciences Prospective Population Study (LOLIPOP) at discovery; for the replication phase we studied 1,354 South Asians using blood sample collected at the follow-up visit.<br>For eQTL analysis, to investigate the robustness of the identified eQTLs from eQTLgen in South Asians, we performed lookups in eQTL dataset derived from 693 South Asians, and also by interrogating islet-specific eQTLs.<br>For PRS analyses, we also replicated model performance of the SA-PRS and EUR-PRS in two independent testing sets (SINDI; n=974 cases and 1,168 controls; LOLIPOP-GSA: n=1,000 cases and controls each).
Randomization	NA
Blinding	NA

Reporting for specific materials, systems and methods

We require information from authors about some types of materials, experimental systems and methods used in many studies. Here, indicate whether each material, system or method listed is relevant to your study. If you are not sure if a list item applies to your research, read the appropriate section before selecting a response.

Materials & experimental systems

n/a	Involved in the study
☒	☐ Antibodies
☒	☐ Eukaryotic cell lines
☒	☐ Palaeontology and archaeology
☒	☐ Animals and other organisms
☐	☒ Human research participants
☒	☐ Clinical data
☒	☐ Dual use research of concern

Methods

n/a	Involved in the study
☒	☐ ChIP-seq
☒	☐ Flow cytometry
☒	☐ MRI-based neuroimaging

Human research participants

Policy information about studies involving human research participants

Population characteristics	South Asians, T2D cases and controls (across 16 cohorts)<br>T2D cases were defined as having any one of the following: medical history of T2D or T2D treatment, fasting plasma glucose concentration ≥7.0 mmol/L, plasma glucose concentration at 2 hours of OGTT ≥11.1 mmol/L, or HbA1c ≥6.5%.<br>Normoglycaemic controls were defined as meeting all of the following criteria (where data are available): no history of T2D or T2D treatment, fasting plasma glucose <6.1 mmol/L, plasma glucose concentration at 2 hours of OGTT <7.8 mmol/L, and HbA1c <6.0%.<br>Supplementary text and ST1 also provides further details for individual cohorts.
Recruitment	Please see Supplementary for description of recruitment for individual cohorts
Ethics oversight	Please see Supplementary for description on ethics for individual cohorts

Note that full information on the approval of the study protocol must also be provided in the manuscript.
